# DCAF7 recruits USP2 to facilitate hepatocellular carcinoma progression by suppressing clockophagy-induced ferroptosis

**DOI:** 10.1038/s41419-025-07977-3

**Published:** 2025-08-28

**Authors:** Honglv Jiang, Xiaohui Wang, Zhenhua Zhu, Cheng Song, Dan Li, Yixuan Yun, Li Hui, Leilei Bao, Darran P. O’Connor, Jingjing Ma, Guoqiang Xu

**Affiliations:** 1 Jiangsu Key Laboratory of Drug Discovery and Translational Research for Brain Diseases and College of Pharmaceutical Sciences, The Fourth Affiliated Hospital of Soochow University, Jiangsu Province Engineering Research Center of Precision Diagnostics and Therapeutics Development, Jiangsu Key Laboratory of Preventive and Translational Medicine for Major Chronic Non-communicable Diseases, Suzhou Key Laboratory of Drug Research for Prevention and Treatment of Hyperlipidemic Diseases, Soochow University, Suzhou, Jiangsu China; 2 Research Center of Biological Psychiatry, Suzhou Guangji Hospital, Suzhou Medical College of Soochow University, Suzhou, Jiangsu China; 3 Department of Pharmacy, Shanghai Eastern Hepatobiliary Surgery Hospital, Shanghai, China; 4 Department of Pharmacy and Biomolecular Sciences, Royal College of Surgeons in Ireland, Dublin, Ireland; 5 Department of Pharmacy, The Fourth Affiliated Hospital of Soochow University, Suzhou Dushu Lake Hospital, Medical Center of Soochow University, Suzhou, Jiangsu China; 6 Suzhou International Joint Laboratory for Diagnosis and Treatment of Brain Diseases, College of Pharmaceutical Sciences, Soochow University, Suzhou, Jiangsu China; 7 MOE Key Laboratory of Geriatric Diseases and Immunology, Suzhou Medical College of Soochow University, Suzhou, Jiangsu Province China; 8 Suzhou Key Laboratory of Geriatric Neurological Disorders, the First People’s Hospital of Taicang, Taicang Affiliated Hospital of Soochow University, Suzhou, Jiangsu China

**Keywords:** Tumour biomarkers, Autophagy

## Abstract

DDB1- and CUL4-associated factor 7 (DCAF7) has recently been identified as a critical regulator of tumorigenesis and a potential modulator of ferroptosis. However, the precise function of DCAF7 in regulating the progression of hepatocellular carcinoma (HCC) ferroptosis remains elusive. In this study, we demonstrate that DCAF7 and the deubiquitinase USP2 are highly expressed in HCC. Genetic ablation of *DCAF7* or pharmacological inhibition of USP2 sensitizes HCC to ferroptosis and inhibits HCC progression both in vitro and in vivo. Mechanistically, DCAF7 recruits USP2 to inhibit clockophagy (the selective autophagic degradation of core clock protein BMAL1 mediated through p62/SQSTM1) by reducing BMAL1 K63-linked polyubiquitination. Targeting either DCAF7 or USP2 triggers clockophagy-induced ferroptosis through the HIF1α-SLC7A11 axis in HCC cells. Collectively, our study establishes DCAF7 and USP2 as novel suppressors of clockophagy-induced ferroptosis and reveals the potential therapeutic targets for HCC treatment.

## Introduction

DCAF7 (also known as WDR68 or HAN11) is a substrate receptor of the cullin 4 (CUL4)-RING E3 ligase (CRL4) complex, which can recruit the specific substrates for their ubiquitination [1]. Interestingly, DCAF7 could also function as a scaffold protein to promote protein-protein interaction [2–4]. Recently, DCAF7 was identified as a critical modulator of drug sensitivity and plays important roles in pancreatic neuroendocrine tumors and the metastasis of nasopharyngeal carcinoma [1, 2]. Moreover, bioinformatic analyses suggest that DCAF7 may execute a pivotal function in ferroptosis, especially in HCC [5–7]. However, the function of DCAF7 in HCC progression, particularly its regulation of ferroptosis, remains unclear.

As an iron-dependent form of cell death driven by lipid peroxidation, ferroptosis has emerged as a critical process in cancer biology. Recent advances have highlighted the role of selective autophagy, including ferritinophagy, lipophagy, and clockophagy in regulating ferroptosis. Notably, clockophagy, the selective autophagic degradation of Brain and muscle ARNT-like 1 (BMAL1 or ARNTL), the core circadian clock protein, by the autophagy cargo receptor sequestosome-1 (SQSTM1 or p62) in response to the ferroptosis inducer RSL3, which is crucial for ferroptosis [8, 9]. In that work, the authors disclosed that the ferroptosis inducer RSL3 enhanced the interaction between BMAL1 and p62 and mediated the autophagic degradation of BMAL1 to induce ferroptosis [9]. However, the detailed regulatory mechanism and the role of clockophagy in HCC progression remain to be elucidated.

Ubiquitin-specific protease 2 (USP2) is a deubiquitinating enzyme that contains 4 isoforms, including USP2-1 (USP2a), USP2-2 (USP2b), USP2-3, and USP2-4. While USP2-3 and USP2-4 remain poorly characterized, USP2b has been primarily implicated in regulating the stability and turnover of BMAL1 [10]. Interestingly, substantial evidence supports the pro-tumorigenic role of USP2a. For instance, USP2a stabilized Twist and promoted the progression of triple-negative breast cancer [11]. Moreover, the USP2 inhibitor ML364 exhibited the capability to enhance ErbB2 ubiquitination and accelerate its turnover, thus inhibiting the growth of ErbB2-positive breast cancer [12]. Despite these insights, the role of USP2 in HCC remains largely unexplored, particularly its involvement in HCC ferroptosis.

Here, we uncover that DCAF7 and USP2 expression are significantly elevated in HCC tissues and provide compelling evidence that genetic depletion of *DCAF7* or pharmacological inhibition of USP2 sensitizes HCC to sorafenib and inhibits HCC progression both in vitro and in vivo. Mechanistically, DCAF7 recruits USP2 to stabilize BMAL1 by reducing its K63-linked polyubiquitination, thereby preventing p62-mediated autophagic degradation of BMAL1 (clockophagy). Targeting either DCAF7 or USP2 elevates clockophagy-induced ferroptosis through the HIF1α-SLC7A11 axis in HCC cells. Collectively, our findings identify DCAF7 and USP2 as novel suppressors of clockophagy-mediated ferroptosis and propose an innovative combination therapy for HCC.

## Methods

### Chemicals

RSL3 (HY-100218A, RRID: SCR_023060), erastin (HY-15763, RRID: SCR_023067), ferrostatin-1 (Fer-1, HY-100579, RRID: SCR_023061), sorafenib (HY-10201, RRID: SCR_023062), ML364 (HY-100900, RRID: SCR_023063), MG132 (HY-13259, RRID: SCR_023064), bafilomycin A1 (BafA1, HY-100558, RRID: SCR_023065), chloroquine (CQ, HY-17589A, RRID: SCR_023066), MLN4924 (HY-70062), and protease inhibitor cocktail mini-tablet (EDTA-free, HY-K0011, RRID: SCR_022150) were purchased from MedChemExpress (MCE). CoCl_2_ (409332) was ordered from Sigma–Aldrich.

### Cell lines and cell culture

HEK293T (RRID: CVCL_0063) cells were obtained from the American Type Culture Collection (ATCC). Huh7 (RRID: CVCL_0336) and SNU-449 (RRID: CVCL_0454) cells were purchased from CELLCOOK (Guangzhou, China). HepG2 (RRID: CVCL_0027, BNCC338070) and SMMC-7721 (BNCC338089) cells were ordered from BeNa Culture Collection (Beijing, China). HEK293T, Huh7, HepG2, and SMMC-7721 cells were cultured in DMEM (C11995500BT, high glucose, Gibco, RRID: SCR_018045) containing 10% FBS (F0193, Sigma–Aldrich, RRID: SCR_018046), 100 μg/mL streptomycin, and 100 units/mL penicillin (C100C5, NCM Biotech, RRID: SCR_023059). SNU-449 cells were maintained in RPMI 1640 medium (C11875500BT, Gibco, RRID: SCR_018044) supplemented with 10% FBS and penicillin/streptomycin. All cell lines were cultured at 37 °C under a humidified environment containing 5% CO_2_.

### Immunoblotting analysis

Cell lysates were mixed with 5× SDS loading buffer and heated at 95 °C for 10 min For SLC7A11 immunoblotting, samples were denatured at 37 °C for 30 min. Proteins were resolved by SDS-PAGE and transferred onto PVDF membranes (Millipore, IPVH00010, RRID: SCR_018043). Membranes were blocked with 10% non-fat milk in TBST for 1 h at room temperature, followed by incubation with primary antibodies (see Table S1) overnight at 4 °C and HRP-conjugated secondary antibodies for 1 h at room temperature, respectively. The signals were visualized with a Super ECL chemiluminescent substrate kit (P10300, NCM Biotech, RRID: SCR_023058) and imaged on a Tanon 5200 system. The signal intensity was analyzed using the ImageJ software (NIH, RRID: SCR_003070).

### RNA extraction and qRT-PCR

TRIzol reagent (R401-01, Vazyme) was used to extract and purify total RNA. cDNA library was synthesized using 5× All-In-One RT MasterMix (G490, ABM). Gene expression was quantified with qPCR using target-specific primers (Table S2, GENEWIZ) and ChamQ SYBR Master Mix (Q511-02, Vazyme). Reactions were performed on a CFX96 Touch Real-Time PCR System (Bio-Rad), and data were collected using CFX Manager software (Bio-Rad, version 3.1, RRID: SCR_017251). The relative gene expression level was obtained using the 2^−ΔΔCt^ method after normalization to *GAPDH* (loading control).

### siRNA and plasmid transfection

siRNAs against human *DCAF7* (Table S3) were purchased from Sangon Biotech (Shanghai) and introduced into cells utilizing RNATransMate (E607402, Sangon Biotech) transfection reagent. Plasmids were transfected into HEK293T and HCC cells using polyethyleneimine (PEI, 919012, Sigma–Aldrich) or lipofectamine 2000 (11668030, ThermoFisher Scientific, RRID: SCR_015663), respectively.

### Cell viability assay

A CCK-8 kit was utilized to analyze cell viability and obtain the growth curve. Briefly, cells transfected with the designated shRNA/siRNA or plasmids were seeded in 96-well plates and treated as indicated. CCK-8 (10 μL) and medium (90 μL) were added to each well, and the cells were cultured at 37 °C for an additional 1 h. The optical density (OD) at 450 nm was detected under a microplate reader (TECAN), and the relative cell viability was normalized by the OD_450_ for cells treated with DMSO (control samples).

### Colony formation assay

After being transfected with the siRNA/shRNA or plasmid, cells were plated (2000 cells/well) in 6-well plates and grew for 14 days. The cells were then fixed with 4% paraformaldehyde, washed with PBS, stained with 0.1% crystal violet, and washed extensively again with PBS. Images were captured and quantified using ImageJ.

### Cellular ROS analysis

The Reactive Oxygen Species Assay Kit (S0033S, Beyotime) was used to assess intracellular ROS. Cells were plated in 6-well plates, treated as designated, and then incubated with DCFH-DA (10 μM in 500 μL serum-free medium) for 30 min at 37 °C. Subsequently, cells were collected, washed twice with PBS, resuspended in 500 μL PBS, and passed through a 70 μm cell strainer (Falcon, 352235) to obtain a single-cell suspension. Flow cytometry analysis was performed using a CYTOPLEX cytometer (Beckman) with a 488 nm excitation laser, and 10,000 events were recorded per sample. Data were processed using FlowJo software (TreeStar, Woodburn, RRID: SCR_008520).

### MDA measurement

MDA was quantified using a thiobarbituric acid (TBA) assay kit (Nanjing Jiancheng, A003-1-2). Cell pellets or tumor tissues were homogenized in lysis buffer with ultrasonication. Equal volumes of lysates were mixed with the working reagent and heated at 95 °C for 1 h. Samples were rapidly cooled and centrifuged at 1500 × *g* for 10 min. Absorbance at 530 nm was measured in quadruplicate in a TECAN microplate reader. Protein concentrations were determined in parallel using the BCA assay (ThermoFisher Scientific, 23225). All experiments were performed in triplicate.

### GSH assay

Intracellular GSH levels were determined by the CheKine Micro Reduced Glutathione Assay Kit (KTB1600, Abbkine). Cells (2 × 10⁶) were washed with PBS, lysed in extraction buffer (3× pellet volume), and subjected to three freeze-thaw cycles in liquid nitrogen and a 37 °C water bath. The samples were then centrifuged at 8000 × *g* for 10 min to obtain clear cell lysates, which were mixed with assay solution alongside standard and blank controls. After 2-min incubation at room temperature, absorbance was measured at 412 nm in quadruplicate using a TECAN microplate reader. GSH concentrations (μg/mL) were determined from a standard curve. Experiments were performed in triplicate for statistical analysis.

### Tissue microarray and immunohistochemical (IHC) analysis

A tissue microarray was generated using 75 paired HCC and adjacent non-tumorous formalin-fixed, paraffin-embedded tissues. IHC was performed with anti-DCAF7 (1:500), USP2 (1:200), and BMAL1 (1:1000) antibodies, followed by hematoxylin counterstaining. Digital images were acquired using Aperio Versa (Leica Biosystems) and quantified using the ImageJ software “IHC Profiler” plugin [13, 14].

### Mass spectrometry (MS) analysis and data processing

The DCAF7-interacting proteins were identified through immunoprecipitation and MS analysis according to a method described previously [15]. The detailed sample preparation, MS analysis, and data processing were provided in the supplementary methods.

### Bioinformatic analysis

The HCC datasets were downloaded from the NCBI GEO repository (RRID: SCR_005012). The dataset GSE214846 contains 65 pairs of HCC and adjacent non-HCC tissue samples, and the dataset GSE105130 contains paired 25 HCC and adjacent non-HCC tissue samples. A paired sample *t*-test was utilized to identify differentially expressed genes between HCC tissue samples and paired adjacent non-HCC tissue samples.

Clinical information and RNA-seq data from the TCGA LIHC dataset were analyzed in UALCAN (https://ualcan.path.uab.edu/index.html, RRID: SCR_015827). Student’s *t*-test or one-way ANOVA with a Tukey’s multiple comparisons post hoc test was used to identify the differentially expressed genes.

### In vivo tumor xenograft experiments

Male BALB/c nude mice (3–5 weeks old) were purchased from Shanghai Jihui Laboratory Animal Care Co., Ltd. (China), fed with a standard chow diet, and familiarized for one week in a SPF facility at the Laboratory Animal Center of Soochow University.

To evaluate the role of DCAF7 on tumor growth in vivo, the negative control (*NC*) or *DCAF7* stable knockdown Huh7 cells (5 × 10^6^) were resuspended in 50 μL PBS and 50 μL Matrigel (BD Biosciences) and subcutaneously injected into the right flank of nude mice. After that, the mice were treated with vehicle, sorafenib (20 mg/kg, gavage), and Fer-1 (1 mg/kg, *i.p*.) once every other day as indicated. Tumor length and width were measured using a caliper every 2 days, and tumor volume was calculated using the formula of volume = (length × width^2^)/2. Body weight measurements were performed concurrently. Mice were sacrificed by cervical dislocation at 28 days, and the tumors were dissected and weighed. Subsequently, the relevant gene expression and the MDA level of the tumor tissues were analyzed by qPCR, Western blotting, and corresponding kits, respectively.

To evaluate the efficacy of the combination therapy with ML364 and sorafenib, Huh7 cells (5 × 10^6^) were implanted into the right flank of nude mice. When the tumor size reached 100–200 mm^3^, mice were randomly divided into 4 groups and treated with vehicle, sorafenib (20 mg/kg, gavage), ML364 (30 mg/kg, *i.p*.), or their combination once every other day as indicated. Tumor size and body weight were measured as described above. At the end of the experiments, mice were sacrificed, and the tumors were dissected and weighed. Similarly, the relevant gene expression and the MDA level of the tumor tissues were further analyzed. We confirmed that the maximal tumor size/burden did not reach 2000 mm^3^.

### Statistical analysis

Experiments were performed with at least three biological replicates, and data were presented as the mean ± standard deviation (SD). GraphPad Prism 10 (version 10.1.2, USA, RRID: SCR_002798) was used to calculate *P*-values using two-tailed, paired or unpaired Student’s *t*-test, one-way ANOVA followed by Tukey’s multiple comparisons post hoc test, or two-way ANOVA followed by Sidak’s multiple comparisons post hoc test as indicated in the figure legends. *P*-values of 0.05 or less were considered statistically significant.

## Results

### DCAF7 is highly expressed in HCC and promotes HCC progression

**Fig. 1:**
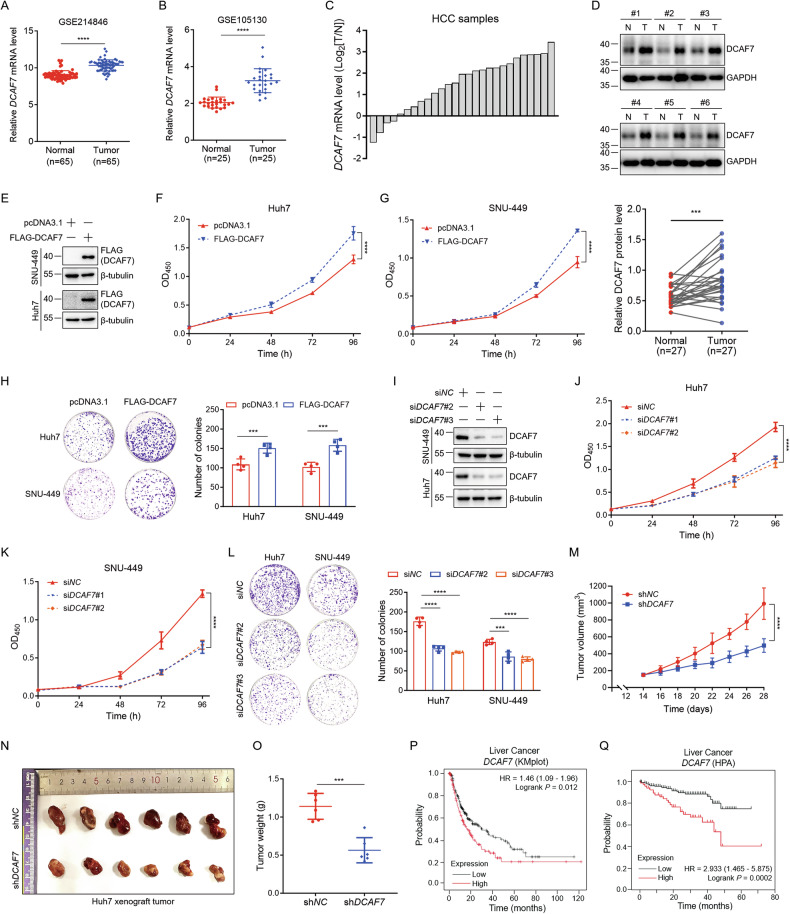
DCAF7 is highly expressed in HCC and promotes HCC progression. **A** and **B** The *DCAF7* mRNA level in normal and HCC tissues obtained from two GEO datasets, GSE214846 and GSE105130. **C** Waterfall plot of the relative *DCAF7* mRNA level measured by qPCR from 27 HCC and paired paratumor tissues. Each bar represents one case. **D** The DCAF7 protein level in HCC and matched paratumor tissues. Mean ± SD (n = 27). **E** Western blotting analysis of the overexpressed DCAF7 in HCC cells. **F** and **G** The OD_450_ of HCC cells transfected with either an empty vector or FLAG-DCAF7 plasmid. Mean ± SD (n = 3, biological replicates). **H** Colony formation assays of HCC cells transfected with either an empty vector or FLAG-DCAF7 plasmid. Mean ± SD (n = 4, biological replicates). **I** Western blotting analysis of cell lysates from the control and *DCAF7*-knockdown HCC cells. **J** and **K** The OD_450_ of HCC cells transfected with si*NC* or si*DCAF7*. Mean ± SD (n = 3, biological replicates). **L** Colony formation assays of HCC cells transfected with si*NC* or si*DCAF7*. Mean ± SD (n = 4, biological replicates). **M**–**O** Xenograft experiment. sh*NC* or sh*DCAF7* Huh7 cells (1 × 10^6^) were subcutaneously injected into nude mice (n = 6 for each group). Tumor volumes (**M**), images (**N**), and tumor weight (**O**) were depicted (mean ± SD). **P** and **Q** The survival analysis of HCC patients with different expression levels of *DCAF7* mRNA. The data were obtained from the Kaplan–Meier plotter database (https://kmplot.com) (**P**) and the Human Protein Atlas database (https://www.proteinatlas.org) (**Q**). The *P*-values were calculated using two-tailed, unpaired Student’s *t*-test (**A**, **B**, **H**, **L**, **O**) or paired Student’s *t*-test (**D**), two-way ANOVA analysis with a Sidak’s multiple comparisons post hoc test (**F**, **G**, **J**, **K**, and **M**), and Log-rank analysis (**P**, **Q**). ***P < 0.001, ****P < 0.0001.

First, we revealed that *DCAF7* mRNA was significantly highly expressed in liver cancer tissues compared to paired adjacent normal tissues, particularly in HCC through the UALCAN database (Fig. S1A, B). Second, analysis of the GEO database confirmed that *DCAF7* mRNA levels were elevated in HCC tissues (Fig. 1A, B). Third, DCAF7 protein levels were also higher in HCC tumor tissues than in normal tissues from the CPTAC proteomic database (Fig. S1C). Consistent with the database analyses, both the mRNA and protein levels of DCAF7 were significantly upregulated in resected HCC tissues compared to adjacent normal tissues obtained from HCC patients (Fig. 1C, D).

We further explored the role of DCAF7 in HCC progression. DCAF7 overexpression enhanced the cell viability and colony formation of HCC cells (Fig. 1E–H and Fig. S1D–G), whereas *DCAF7* knockdown either by siRNA or by shRNA produced the opposite effects (Fig. 1I–L and Fig. S1H–N). Moreover, *DCAF7* knockdown notably suppressed the growth of Huh7 xenografts in vivo (Fig. 1M–O). Additionally, survival analyses demonstrated that high *DCAF7* mRNA expression was positively correlated with the poor prognosis of HCC patients (Fig. 1P, Q and Fig. S1O, P). Altogether, these findings highlight that *DCAF7* is elevated in HCC tissues, and its positive impact on HCC cell growth contributes to its oncogenic effect, underscoring the therapeutic potential of DCAF7 for HCC.

### *DCAF7* deficiency induces ferroptosis to suppress HCC progression through the HIF1α-SLC7A11 axis

**Fig. 2:**
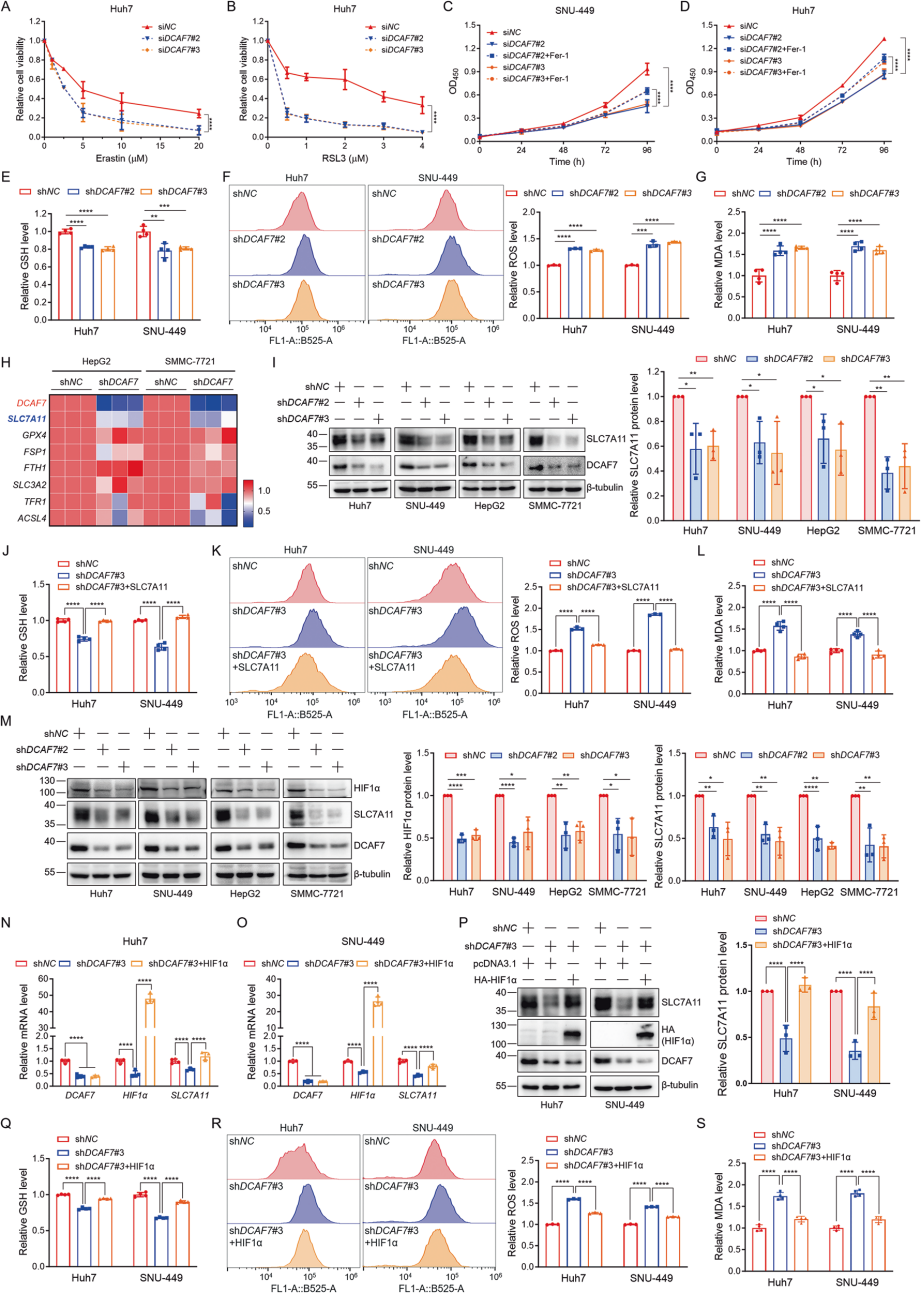
*DCAF7* deficiency induces ferroptosis to suppress HCC progression through the HIF1α-SLC7A11 axis. **A** and **B** The relative cell viability of si*NC* or si*DCAF7*-transfected Huh7 cells treated with different concentrations of the ferroptosis inducer Erastin (**A**) or RSL3 (**B**) for 24 h. Mean ± SD (n = 3, biological replicates). **C** and **D** The OD_450_ of si*NC* or si*DCAF7-*transfected HCC cells treated with DMSO or Fer-1 (1 μM) for different durations. Mean ± SD (n = 3, biological replicates). **E**–**G** The relative intracellular GSH (**E**), ROS (**F**), and MDA (**G**) levels for the control or *DCAF7*-knockdown HCC cells. Mean ± SD (n = 3, biological replicates). **H** qPCR analysis of ferroptosis-related genes in the control or *DCAF7*-knockdown HepG2 and SMMC-7721 cells. **I** Western blotting analysis of SLC7A11 in the control or *DCAF7*-knockdown HCC cells. Mean ± SD (n = 3, biological replicates). **J**–**L** The relative intracellular GSH (**J**), ROS (**K**), and MDA (**L**) levels in the control or *DCAF7*-knockdown Huh7 and SNU-449 cells transfected with an empty vector or HA-SLC7A11 plasmid. Mean ± SD (n = 3, biological replicates). **M** Western blotting analysis of HIF1α and SLC7A11 in the control or *DCAF7*-knockdown HCC cells. Mean ± SD (n = 3, biological replicates). **N**–**P** qPCR and Western blotting analysis of the relative mRNA (**N** and **O**) and protein level (**P**) of SLC7A11 in the control or *DCAF7*-knockdown HCC cells transfected with an empty vector or HA-HIF1α plasmid. Mean ± SD (n = 3, biological replicates). **Q**–**S** The relative intracellular GSH (**Q**), ROS (**R**), and MDA (**S**) levels for the control or *DCAF7*-knockdown Huh7 and SNU-449 cells transfected with an empty vector or HA-HIF1α plasmid. Mean ± SD (n = 3, biological replicates). The *P*-values were calculated using two-tailed, unpaired Student’s *t*-test (**E**–**G**, **I**, and **M**), one-way ANOVA analysis with a Tukey’s multiple comparisons post hoc test (**J**–**L**, and **N**–**S**), and two-way ANOVA analysis with a Sidak’s multiple comparisons post hoc test (**A**–**D**). *P < 0.05, **P < 0.01, ***P < 0.001, ****P < 0.0001.

A bioinformatic study reported that DCAF7 is a putative ferroptosis regulator in HCC [6]. Moreover, our gene set enrichment analysis (GSEA) results further suggested that DCAF7 is a potential indicator for ferroptosis from the TCGA LIHC dataset (Fig. S2A). However, the role of DCAF7 in ferroptosis was still elusive. Strikingly, we found *DCAF7* knockdown sensitized HCC cells to both ferroptosis inducers erastin and RSL3 (Fig. 2A, B and Fig. S2B, C). Moreover, ferrostatin-1 (Fer-1), the ferroptosis inhibitor, partially alleviated the inhibitory effect of *DCAF7* knockdown on the HCC cell growth (Fig. 2C, D), indicating that *DCAF7* knockdown induced cell death, at least partly, through ferroptosis. Supportively, the GSH level was decreased (Fig. 2E), while the levels of ROS (Fig. 2F) and MDA (Fig. 2G) were upregulated in the *DCAF7*-knockdown HCC cells. Taken together, our data substantiate that depletion of *DCAF7* induces ferroptosis.

To delineate the precise molecular pathways through which DCAF7 modulates ferroptosis, the expression of a series of ferroptosis-related genes was examined using qPCR. Among these genes, only *SLC7A11* was reduced markedly in *DCAF7*-knockdown HCC cells (Fig. 2H). Consistently, the SLC7A11 protein was significantly decreased in *DCAF7*-knockdown HCC cells (Fig. 2I). Furthermore, *DCAF7* and *SLC7A11* expression was positively correlated in the LIHC database (Fig. S2D). Moreover, SLC7A11 overexpression (Fig. S2E) could reverse the decrease in the GSH level (Fig. 2J) and the increase in the ROS (Fig. 2K) and MDA (Fig. 2L) levels triggered by *DCAF7* knockdown. Collectively, these findings suggest that DCAF7 modulates ferroptosis primarily through SLC7A11.

Next, we aimed to identify the transcription factor of *SLC7A11* modulated by DCAF7. Given that previous studies have demonstrated that HIF1α [16, 17], Nrf2 [18, 19], p53 [20, 21], and ATF4 [22] are critical transcription factors for *SLC7A11* in regulating ferroptosis, we examined the effect of DCAF7 on their protein levels. Strikingly, Nrf2 and ATF4 expression remained unaffected following *DCAF7* knockdown, whereas HIF1α and p53 protein levels significantly reduced in HCC cells (Fig. 2M and Fig. S2F, G). Given that p53 serves as a transcriptional repressor of *SLC7A11* [21], and considering that *DCAF7* knockdown reduced SLC7A11 protein even under *p53*-deficient conditions (Fig. S2H), we thus conclude that DCAF7 likely regulates *SLC7A11* transcription predominantly through HIF1α rather than p53.

Indeed, the decrease of HIF1α was further confirmed under CoCl₂-induced hypoxia conditions (Fig. S2I). Moreover, the downregulation of SLC7A11 mediated by *DCAF7* knockdown was attenuated when HIF1α was overexpressed (Fig. 2N–P). Consistently, HIF1α overexpression could reverse the reduction of GSH levels (Fig. 2Q) and the elevation of ROS (Fig. 2R) and MDA (Fig. 2S) levels caused by *DCAF7* knockdown. Besides, HIF1α overexpression partially mitigated the inhibition of HCC cell growth upon *DCAF7* knockdown (Fig. S2J, K). Furthermore, *DCAF7* knockdown significantly reduced the transcription of *HIF1α* and *SLC7A11* (Fig. S2L, M). Coherently, analysis of the LIHC database demonstrated significant positive correlations between *DCAF7*-*HIF1α* and *HIF1α*-*SLC7A11* mRNA levels (Fig. S2N, O). Taken together, these findings indicate that *DCAF7* depletion induces ferroptosis partly through the HIF1α-SLC7A11 axis.

### DCAF7 stabilizes BMAL1 protein to upregulate *HIF1α* transcription

**Fig. 3:**
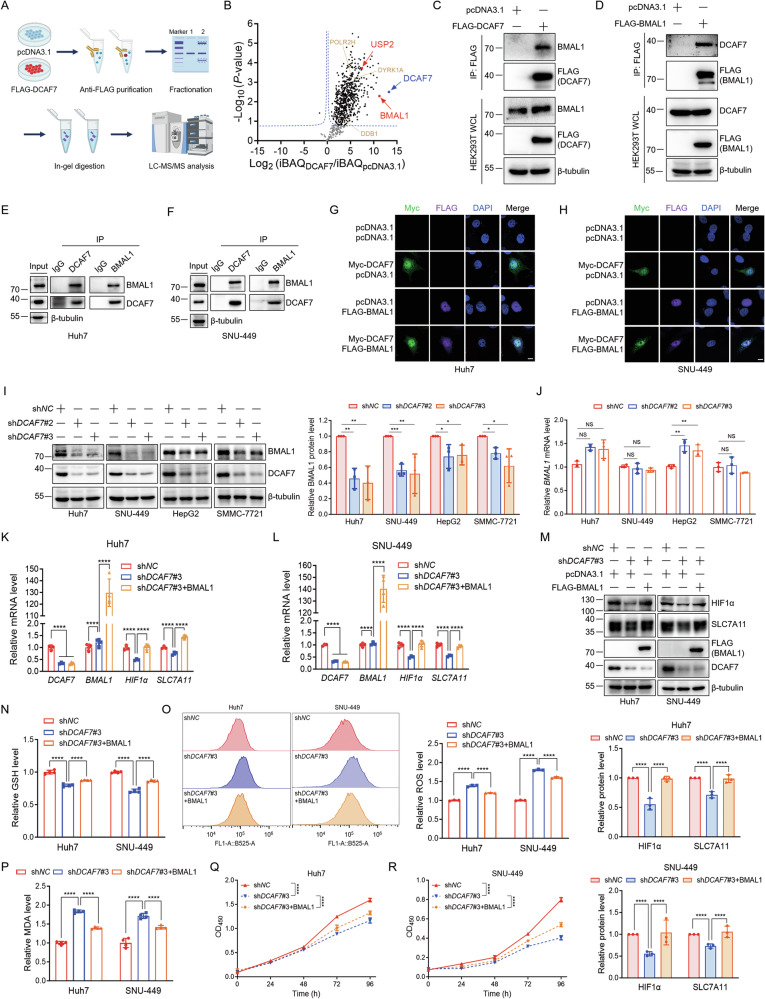
DCAF7 stabilizes BMAL1 to upregulate *HIF1α* transcription. **A** and **B** IP-MS/MS analysis of the DCAF7-interacting proteins. The schematic (**A**) and the volcano plot (**B**) of the DCAF7-interacting proteins obtained by IP-MS/MS analyses. DYRK1A, DDB1, and POLR2H were labeled as reported positive DCAF7-interacting proteins in the volcano plots. **C**–**F** Co-immunoprecipitation of DCAF7 and BMAL1 exogenously or endogenously. **G** and **H** Immunofluorescence analyses of the colocalization of DCAF7 and BMAL1 in Huh7 (**G**) and SNU-449 (**H**) cells. Scar bar: 10 μm. **I** and **J** Western blotting and qPCR analyses of BMAL1 protein (**I**) and mRNA (**J**) levels in the control or *DCAF7*-knockdown HCC cells. Mean ± SD (n = 3, biological replicates). **K**–**M** qPCR analyses of the relative *HIF1α* and *SLC7A11* mRNA levels (**K** and **L**) and Western blotting analyses of HIF1α and SLC7A11 protein (**M**) in the control or *DCAF7*-knockdown Huh7 and SNU-449 cells transfected with an empty vector or FLAG-BMAL1 plasmid. Mean ± SD (n = 3, biological replicates). **N**–**P** The relative intracellular GSH (**N**), ROS (**O**), and MDA (**P**) levels in the control or *DCAF7*-knockdown Huh7 and SNU-449 cells transfected with an empty vector or FLAG-BMAL1 plasmid. Mean ± SD (n = 3, biological replicates). **Q** and **R** The OD_450_ of the control or *DCAF7*-knockdown HCC cells transfected with an empty vector or FLAG-BMAL1 plasmid. Mean ± SD (n = 3, biological replicates). The *P*-values were calculated using two-tailed, unpaired Student’s *t*-test (**I** and **J**), one-way ANOVA analysis with a Tukey’s multiple comparisons post hoc test (**K**–**P**), and two-way ANOVA analysis with a Sidak’s multiple comparisons post-test (**Q** and **R**). NS, *P* > 0.05, *P < 0.05, **P < 0.01, ***P < 0.001, ****P < 0.0001.

To explore the mechanism by which DCAF7 regulates *HIF1α* transcription, we performed IP-MS/MS analysis and identified the core clock protein BMAL1 as a DCAF7-interacting protein (Fig. 3A, B and Fig. S3A). Co-immunoprecipitation and immunofluorescence further verified that DCAF7 interacted and colocalized with BMAL1, respectively (Fig. 3C–H). Additionally, *DCAF7* knockdown significantly decreased the BMAL1 protein level without reducing its mRNA level (Fig. 3I, J and Fig. S3B, C), while DCAF7 overexpression stabilized the BMAL1 protein (Fig. S3D–G).

It has been reported that BMAL1 functions as a *HIF1α* transcription factor [23]. Aligning with these findings, our ChIP-qPCR analysis demonstrated the direct binding of BMAL1 to the E-box-containing region (−522 to −233) in the *HIF1α* promoter (Fig. S3H). We thus hypothesized that BMAL1 might mediate the DCAF7-regulated *HIF1α* transcription. As expected, BMAL1 overexpression rescued the downregulation of *HIF1α* and its downstream *SLC7A11* expression induced by *DCAF7*-knockdown (Fig. 3K–M and Fig. S3I–K). Furthermore, BMAL1 could partially reverse the reduction of GSH (Fig. 3N) and the upregulation of ROS (Fig. 3O) and MDA (Fig. 3P and Fig. S3L) in *DCAF7*-knockdown HCC cells. In parallel, BMAL1 could also partially rescue the growth-inhibitory effects induced by *DCAF7* knockdown in HCC cells (Fig. 3Q, R). Collectively, our work suggests that DCAF7 stabilized BMAL1 protein to regulate ferroptosis through the HIF1α-SLC7A11 axis.

### DCAF7 inhibits clockophagy to stabilize BMAL1

**Fig. 4:**
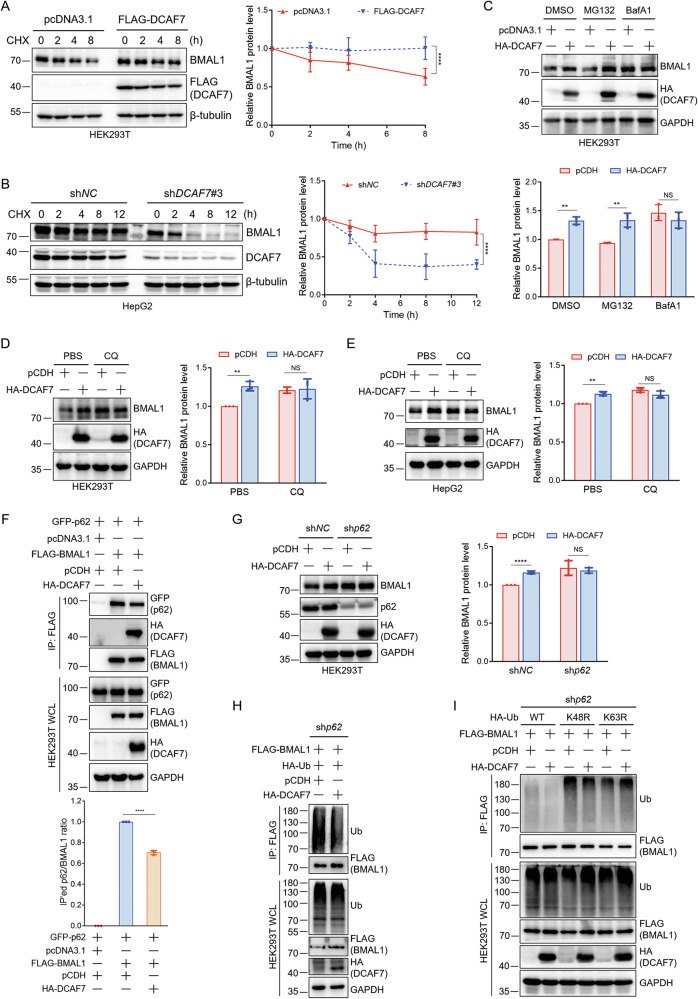
DCAF7 inhibits clockophagy to stabilize BMAL1. **A** and **B** Analysis of the BMAL1 protein turnover under DCAF7 overexpression (**A**) or knockdown (**B**) conditions. Cycloheximide (CHX): 200 μg/mL. Mean ± SD (n = 3, biological replicates). **C** Western blotting analysis and quantification of BMAL1 in HEK293T cells transfected with an empty vector or HA-DCAF7 plasmid in the absence or presence of MG132 (10 μM) or BafA1 (100 nM), respectively, for 12 h. Mean ± SD (n = 3, biological replicates). **D** and **E** Western blotting analysis and quantification of BMAL1 in HEK293T (**D**) and HepG2 (**E**) cells transfected with an empty vector or HA-DCAF7 plasmid in the absence or presence of CQ (50 μM) for 12 h. Mean ± SD (n = 3, biological replicates). **F** Analysis and quantification of the BMAL1-p62 interaction in the absence or presence of DCAF7. Mean ± SD (n = 3, biological replicates). **G** Western blotting analysis and quantification of the effect of DCAF7 on BMAL1 protein level in the control or *p62*-knockdown HEK293T cells. Mean ± SD (n = 3, biological replicates). **H** Analysis of the BMAL1 ubiquitination in the absence or presence of DCAF7 in *p62*-knockdown HEK293T cells. **I** Analysis of the type of polyubiquitin chain on BMAL1 regulated by DCAF7 in the *p62*-knockdown HEK293T cells. The *P*-values were calculated using a two-tailed, unpaired Student’s *t*-test (**C**–**G**) and two-way ANOVA analysis with a Sidak’s multiple comparisons post-test (**A**, **B**). NS, *P* > 0.05, **P < 0.01, ****P < 0.0001.

To further address how DCAF7 stabilizes BMAL1, we performed a cycloheximide (CHX) chase assay and revealed that DCAF7 overexpression markedly delayed BMAL1 degradation (Fig. 4A), whereas *DCAF7* knockdown accelerated BMAL1 turnover (Fig. 4B). Furthermore, we discovered that the autophagy inhibitors bafilomycin A1 (BafA1) and chloroquine (CQ), rather than the proteasome inhibitor MG132, blocked the DCAF7-mediated upregulation of BMAL1 (Fig. 4C–E), suggesting that DCAF7 stabilizes BMAL1 through inhibiting its autophagic degradation.

Previous studies have demonstrated that p62 functions as a selective autophagy receptor in clockophagy by mediating BMAL1 recognition and autophagic degradation through its ubiquitin-associated (UBA) domain [9]. Thus, it is reasonable to speculate that p62 may participate in the DCAF7-mediated inhibition of BMAL1 autophagic degradation. Indeed, DCAF7 overexpression significantly decreased the interaction between BMAL1 and p62 (Fig. 4F). Moreover, the upregulation of BMAL1 by DCAF7 was blocked by *p62* knockdown (Fig. 4G). Given that the UBA domain in p62 specifically recruits ubiquitinated proteins to an autophagosome for lysosomal degradation [9], we thus assessed the effect of DCAF7 on BMAL1 ubiquitination. As expected, DCAF7 reduced BMAL1 ubiquitination significantly (Fig. 4H), which is mainly the K63-linked polyubiquitination (Fig. 4I).

Collectively, these results demonstrate that DCAF7 inhibits clockophagy through the sequential mechanism: DCAF7 attenuates BMAL1 K63-linked polyubiquitination, reduces BMAL1-p62 interaction, ultimately blocks clockophagy, and thus enhances its stability.

### DCAF7 recruits USP2 to deubiquitinate BMAL1 and inhibit clockophagy

**Fig. 5:**
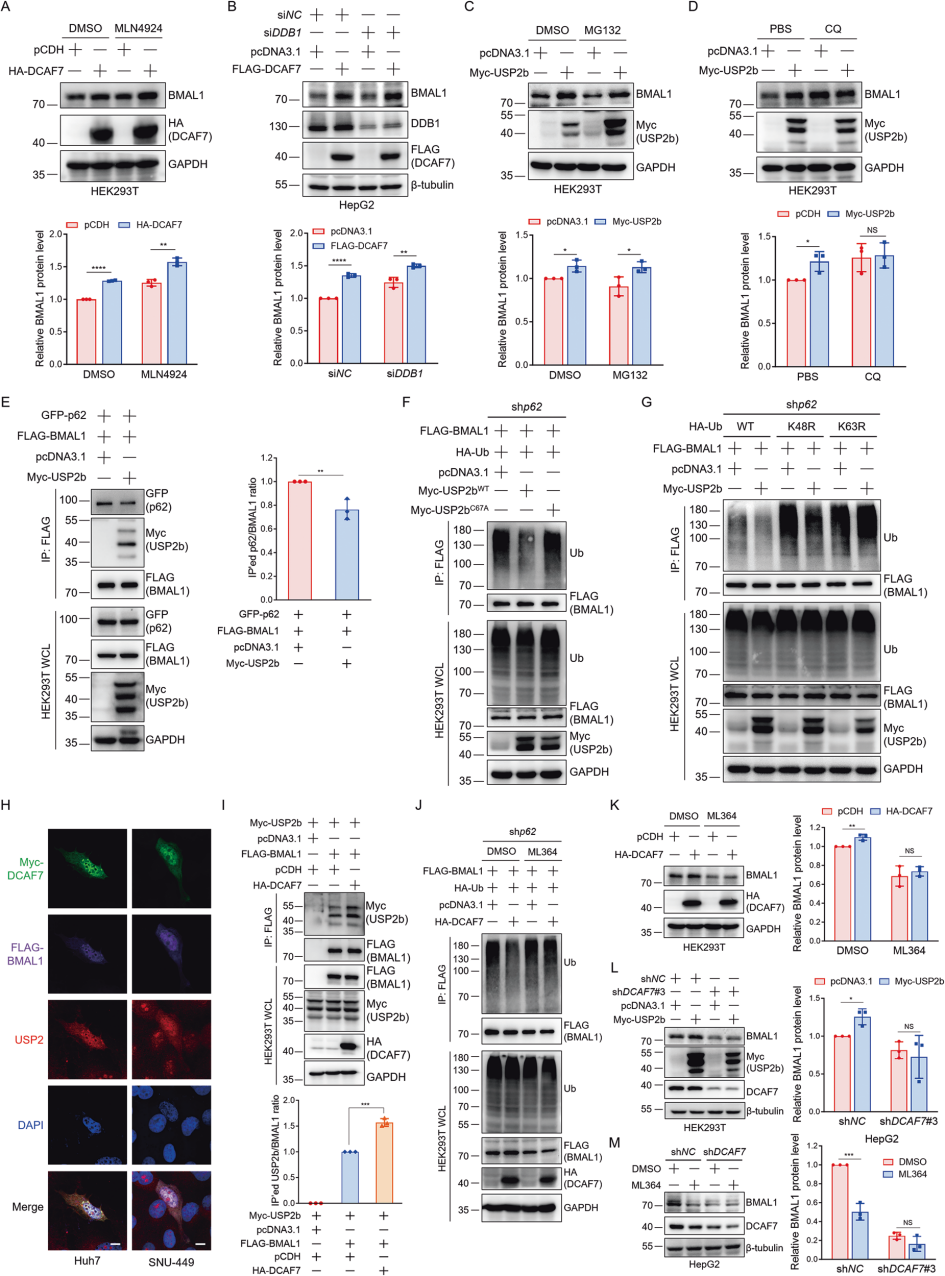
DCAF7 recruits USP2 to deubiquitinate BMAL1 and inhibit clockophagy. **A** Western blotting analysis and quantification of BMAL1 in HEK293T cells transfected with an empty vector or HA-DCAF7 plasmid and treated with or without MLN4924 (1 μM) for 24 h. Mean ± SD (n = 3, biological replicates). **B** Western blotting analysis and quantification of the effect of DCAF7 on BMAL1 protein level in the control or *DDB1*-knockdown HepG2 cells. Mean ± SD (n = 3, biological replicates). **C** and **D** Western blotting analysis and quantification of BMAL1 in HEK293T cells transfected with an empty vector or Myc-USP2b plasmid and treated with or without MG132 (10 μM) (**C**), or CQ (50 μM) (**D**) for 12 h, respectively. Mean ± SD (n = 3, biological replicates). **E** Analysis of the BMAL1-p62 interaction in the absence or presence of USP2b. Mean ± SD (n = 3, biological replicates). **F** Analysis of the BMAL1 ubiquitination in the absence or presence of USP2b or its catalytically inactive mutant USP2b^C67A^ in *p62*-knockdown HEK293T cells. **G** Analysis of the type of polyubiquitin chain on BMAL1 regulated by USP2b in the *p62*-knockdown HEK293T cells. **H** Immunofluorescence analysis of the colocalization of DCAF7, BMAL1, and USP2 in Huh7 and SNU-449 cells. Scar bar: 10 μm. **I** Analysis and quantification of the BMAL1-USP2b interaction in the absence or presence of DCAF7. Mean ± SD (n = 3, biological replicates). **J** Analysis of the effect of DCAF7 on BMAL1 ubiquitination in *p62*-knockdown HEK293T cells treated with or without the USP2 inhibitor ML364 (10 μM) for 12 h. **K** Western blotting analysis and quantification of the effect of DCAF7 on BMAL1 protein level in HEK293T cells treated with or without ML364 (10 μM) for 12 h. Mean ± SD (n = 3, biological replicates). **L** Western blotting analysis and quantification of the effect of USP2b on BMAL1 protein level in the control (sh*NC*) or *DCAF7*-knockdown (sh*DCAF7*) HEK293T cells. Mean ± SD (n = 3, biological replicates). **M** Western blotting analysis and quantification of the effect of ML364 (10 μM, 12 h) on BMAL1 protein level in the control or *DCAF7*-knockdown HepG2 cells. Mean ± SD (n = 3, biological replicates). The *P*-values were calculated using a two-tailed, unpaired Student’s *t*-test (**A**–**E**, **I**, and **K**–**M**). NS, *P* > 0.05, *P < 0.05, **P < 0.01, ***P < 0.001, ****P < 0.0001.

We next sought to explore how DCAF7 promotes BMAL1 deubiquitination. As a substrate receptor of the CRL4 E3 ligase complex, DCAF7 is known to recruit target proteins for ubiquitination [1, 24]. MLN4924, an inhibitor for the NEDD8-activating enzyme, can suppress CRL4 E3 ligase activity [25]. We examined the effect of DCAF7 on BMAL1 protein in the absence or presence of MLN4924. However, DCAF7 still upregulated BMAL1 in the presence of MLN4924 (Fig. 5A). Consistently, when *DDB1* was knocked down to disrupt the CRL4 complex, DCAF7 continued to elevate BMAL1 levels (Fig. 5B). Thus, these results suggest that DCAF7 stabilizes BMAL1 independent of the CRL4^DCAF7^ E3 ligase activity.

Of note, DCAF7 also serves as a scaffold protein to promote protein-protein interaction. For example, DCAF7 facilitates the interaction between DYRK1A and RNA polymerase II (Pol II), enabling DYRK1A to hyperphosphorylate Pol II [4]. Thus, it is reasonable to speculate that DCAF7 may function as a scaffold protein to promote the interaction between BMAL1 and its deubiquitinase, to promote BMAL1 deubiquitination, and enhance its stabilization.

USP1 [26], USP9X [27], and USP2 [10] are the reported deubiquitinases for BMAL1. Among them, USP1 and USP9X mediated BMAL1 deubiquitination and inhibited its degradation through the ubiquitin-proteasome pathway [26, 27]. However, although USP2 was considered to stabilize BMAL1 by inhibiting its ubiquitin-mediated proteasomal degradation, no evidence directly confirmed this conjecture [10]. To address this, we first evaluated the effect of USP2 on BMAL1 protein. Consistent with previous reports, both USP2 isoforms, USP2b (Fig. S4A) and USP2a (Fig. S4B), significantly upregulated BMAL1 without affecting *BMAL1* and *DCAF7* mRNA levels (Fig. S4C, D). Interestingly, BafA1 and CQ, rather than MG132, blocked the upregulation of BMAL1 induced by USP2 (Fig. 5C, D and Fig. S4E, F). Moreover, similar to DCAF7, USP2 also significantly diminished the interaction between BMAL1 and p62 (Fig. 5E and Fig. S4G), and the upregulation of BMAL1 by USP2 was blocked by *p62* knockdown (Fig. S4H, I). Furthermore, ubiquitination assays demonstrated that USP2 but not its catalytically inactive mutant USP2b^C67A^ reduces BMAL1 polyubiquitination (Fig. 5F and Fig. S4J), specifically decreasing its K63-linked ubiquitin chains (Fig. 5G and Fig. S4K). Collectively, these results demonstrate that USP2 stabilizes BMAL1 by inhibiting clockophagy rather than the ubiquitin-proteasome pathway.

Our quantitative proteomics identified USP2 as a DCAF7-interacting partner (Fig. 3B), and GST pulldown assays confirmed the direct binding between DCAF7 and both USP2 isoforms (USP2a and USP2b) in vitro (Fig. S5A, B). In addition, both DCAF7 and USP2 stabilize BMAL1 by inhibiting clockophagy. Therefore, we propose that USP2 is essential for DCAF7-mediated BMAL1 deubiquitination and stabilization. To test this hypothesis, we first investigate the cellular localization of DCAF7, BMAL1, and USP2. The results indicated that DCAF7, BMAL1, and USP2 colocalized with each other (Fig. 5H). Moreover, DCAF7 notably increased the interaction between USP2 and BMAL1 (Fig. 5I and Fig. S5C), whereas *DCAF7* knockdown markedly reduced the interaction of BMAL1 with both USP2 isoforms (USP2a and USP2b) (Fig. S5D). Critically, pharmacological inhibition of USP2 by ML364 completely abrogated both DCAF7-mediated BMAL1 deubiquitination (Fig. 5J) and the subsequent BMAL1 stabilization (Fig. 5K). Notably, *DCAF7* knockdown abolished the USP2-mediated BMAL1 stabilization and the ML364-induced BMAL1 degradation (Fig. 5L, M), confirming their functional interdependence in BMAL1 proteostasis. In addition, neither *DCAF7* knockdown nor DCAF7 overexpression significantly impacted the USP2 protein level (Fig. S5E, F). In concert with this, *DCAF7* knockdown did not alter the protein level of two USP2 substrates, CCND1 [28] and Snail [29], in HCC cells (Fig. S5G, H). Taken together, our results indicate that DCAF7 specifically recruits USP2 to decrease the K63-linked polyubiquitination of BMAL1, thereby weakening the BMAL1-p62 interaction and subsequently blocking clockophagy, which ultimately stabilizes BMAL1.

### USP2 inhibits DCAF7 autophagic degradation and modulates ferroptosis

**Fig. 6:**
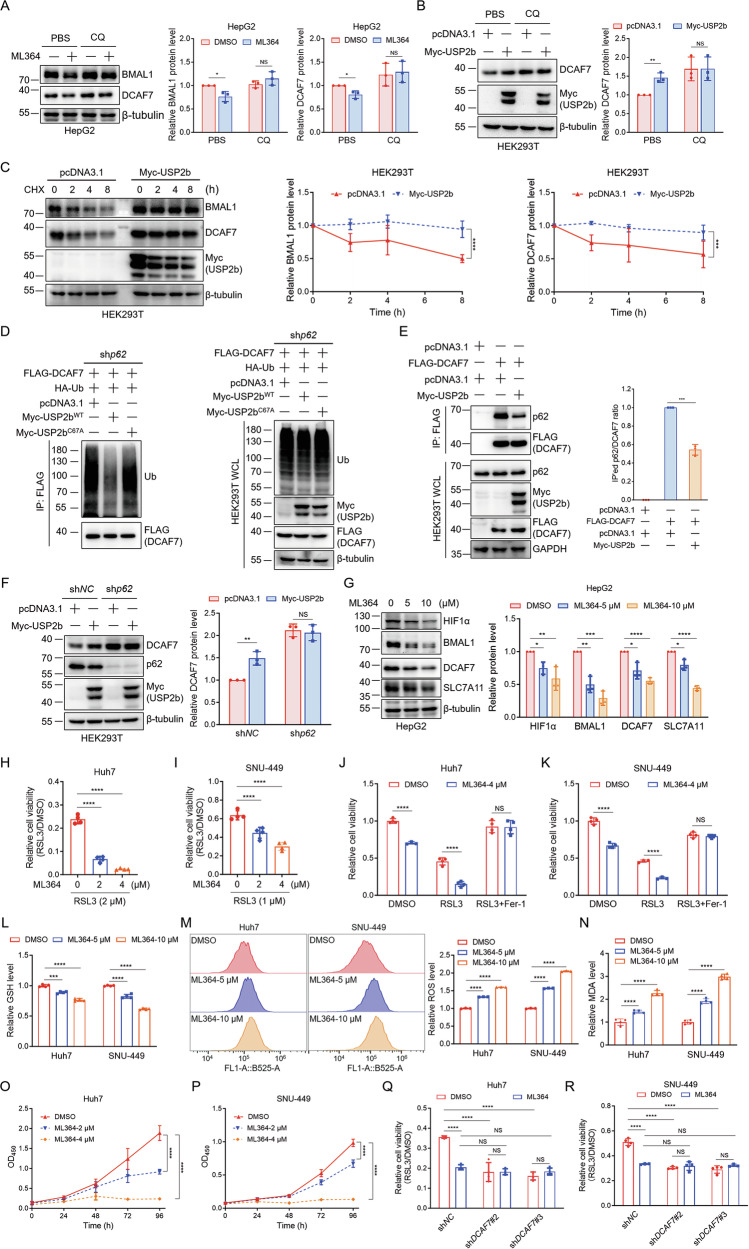
USP2 inhibits DCAF7 autophagic degradation and modulates ferroptosis. **A** Effect of ML364 on DCAF7 and BMAL1 proteins in HepG2 cells treated with PBS or CQ (50 μM) for 12 h. Mean ± SD (n = 3, biological replicates). **B** Effect of USP2b on DCAF7 protein in HEK293T cells treated with PBS or CQ (50 μM) for 12 h. Mean ± SD (n = 3, biological replicates). **C** Analysis of USP2b on the DCAF7 and BMAL1 protein turnover. CHX: 200 μg/mL. Mean ± SD (n = 3, biological replicates). **D** Analysis of USP2b on the DCAF7 ubiquitination in *p62*-knockdown HEK293T cells. **E** Analysis of USP2b on the DCAF7-p62 interaction. Mean ± SD (n = 3, biological replicates). **F** Effect of USP2b on DCAF7 protein in the control or *p62*-knockdown HEK293T cells. Mean ± SD (n = 3, biological replicates). **G** Analysis of the effect of ML364 on DCAF7, BMAL1, HIF1α, and SLC7A11 protein. Mean ± SD (n = 3, biological replicates). **H** and **I** The relative cell viability of HCC cells treated with ML364 and RSL3 for 24 h. Mean ± SD (n = 3, biological replicates). **J** and **K** The relative cell viability of HCC cells treated with DMSO, RSL3 (2 μM for Huh7, 1 μM for SNU-449), or RSL3 and Fer-1(1 μM) and ML364 (4 μM) for 24 h. Mean ± SD (n = 3, biological replicates). **L**–**N** The relative GSH (**L**), ROS (**M**), and MDA (**N**) levels in HCC cells treated with ML364. Mean ± SD (n = 3, biological replicates). **O** and **P** The OD_450_ of the HCC cells treated with ML364. Mean ± SD (n = 3, biological replicates). **Q** and **R** The relative cell viability of the control and *DCAF7*-knockdown HCC cells treated with ML364 and RSL3 for 24 h. Mean ± SD (n = 3, biological replicates). The *P*-values were calculated using two-tailed, unpaired Student’s *t*-test (**A**, **B**, **E**, **F**, **J**, **K**), one-way ANOVA analysis with a Tukey’s multiple comparisons post hoc test (**G**–**I**, **L**–**N**, **Q**, **R**), and two-way ANOVA analysis with a Sidak’s multiple comparisons post hoc test (**C,** **O**, **P**). NS, *P* > 0.05, *P < 0.05, **P < 0.01, ***P < 0.001, ****P < 0.0001.

In the above experiments, we observed an intriguing phenomenon wherein the USP2-specific inhibitor ML364 significantly attenuated DCAF7 (Fig. 5M). This unexpected finding prompted us to further exploit the mechanism by which ML364 modulates DCAF7. Immunoblotting results revealed that CQ, rather than MG132, blocked the downregulation of DCAF7 and BMAL1 upon ML364 treatment (Fig. 6A and Fig. S6A), suggesting that ML364 mediates the autophagic degradation of DCAF7 and BMAL1. Consistently, USP2b significantly upregulated the protein level of DCAF7 without affecting its mRNA level, while these processes can be blocked by CQ (Fig. 6B and Fig. S4D). Moreover, USP2b overexpression markedly extended the half-life of both endogenous DCAF7 and BMAL1 proteins (Fig. 6C). Additionally, ubiquitination assays demonstrated that USP2 enzymatically reduces DCAF7 polyubiquitination (Fig. 6D), specifically decreasing its K63-linked ubiquitin chains (Fig. S6B). Consistent with its role in regulating BMAL1, USP2b also significantly reduced the interaction between DCAF7 and p62 (Fig. 6E), while the enzymatically inactive mutant USP2b^C67A^ lost this function (Fig. S6C). In addition, USP2b was unable to upregulate DCAF7 in the *p62*-depleted cells (Fig. 6F). Collectively, these data validate that USP2 mediates DCAF7 deubiquitination, diminishes the DCAF7-p62 interaction, and inhibits the DCAF7 autophagic degradation.

Next, we tested whether USP2 regulates DCAF7 and BMAL1 downstream ferroptosis-related proteins HIF1α and SLC7A11. Indeed, ML364 not only decreased DCAF7 and BMAL1 protein but also significantly dwindled HIF1α and SLC7A11 protein in HCC cells (Fig. 6G and Fig. S6D). These results prompted us to further explore the potential role of USP2 on ferroptosis. Notably, ML364 enhanced the vulnerability of HCC cells to ferroptosis (Fig. 6H, I), while the ML364-caused RSL3 sensitization could be rescued by Fer-1 (Fig. 6J, K), indicating that the inhibition of USP2 activity by ML364 can induce ferroptosis. Furthermore, ML364 reduced the GSH levels (Fig. 6L) and elevated the ROS (Fig. 6M) and MDA (Fig. 6N) levels in a dose-dependent manner. In addition, ML364 exhibited a marked inhibitory effect on the growth of HCC cells (Fig. 6O, P). Strikingly, *DCAF7* depletion abolished ML364-induced RSL3 sensitization, while ML364 treatment reciprocally attenuated *DCAF7* knockdown-mediated ferroptosis (Fig. 6Q, R). Moreover, USP2 overexpression elevated intracellular GSH levels (Fig. S6E, F) while reducing MDA (Fig. S6G, H) and ROS (Fig. S6I, J) production in HCC cells. Importantly, these anti-ferroptotic effects were blocked by *DCAF7* knockdown, demonstrating a functional cooperation between USP2 and DCAF7 in regulating ferroptosis (Fig. S6E–J). Taken together, these findings support the fact that USP2 inhibition promotes DCAF7 autophagic degradation and induces ferroptosis in HCC cells.

### Targeting DCAF7 or USP2 sensitizes HCC cells to sorafenib by inducing ferroptosis

**Fig. 7:**
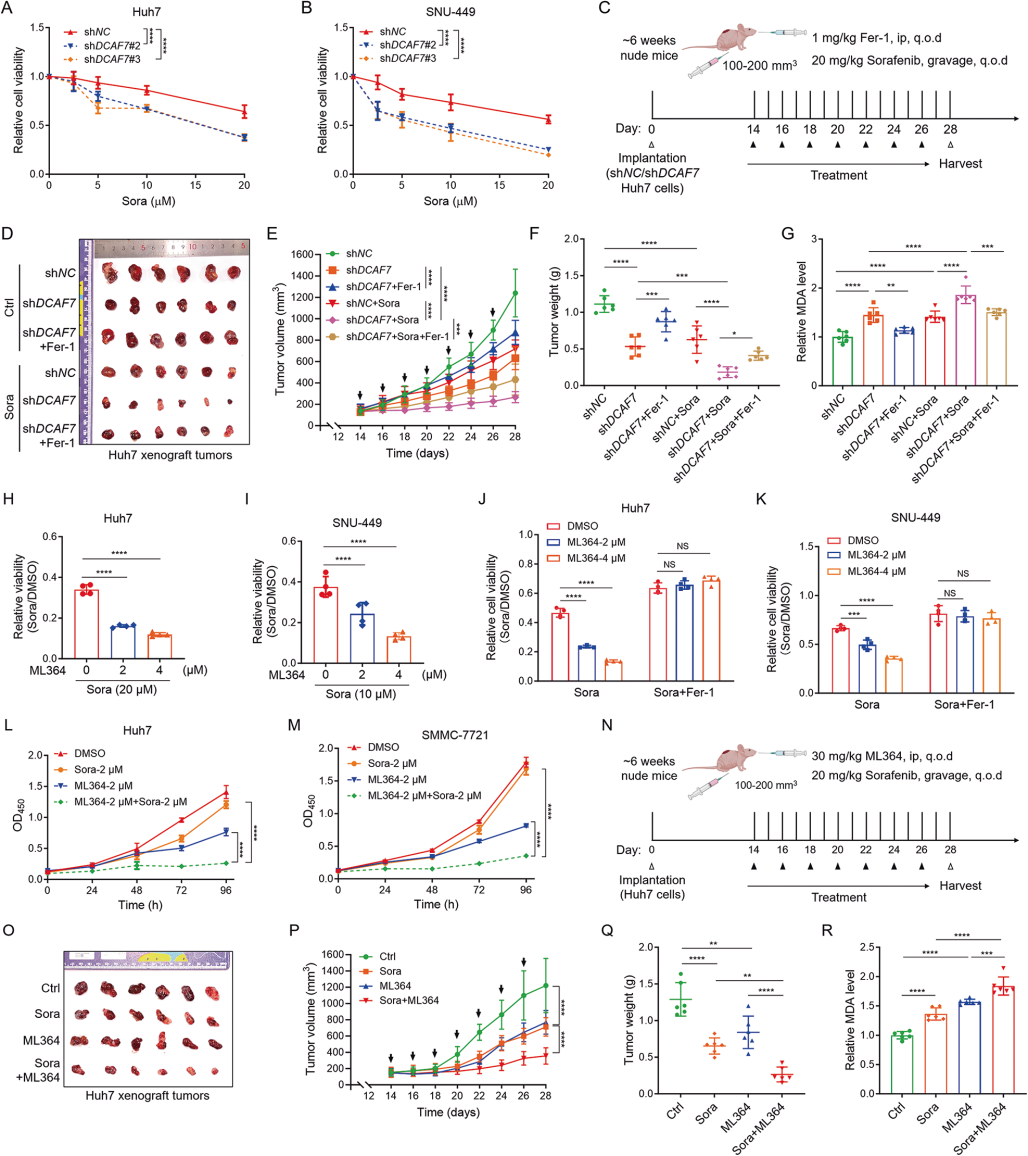
Targeting DCAF7 or USP2 sensitizes HCC cells to sorafenib by inducing ferroptosis. **A** and **B** The relative cell viability of the sh*NC* and sh*DCAF7*-HCC cells treated with different concentrations of sorafenib for 24 h. Mean ± SD (n = 3, biological replicates). **C** Schematic representation of the treatment schedule of sorafenib and Fer-1 for sh*NC* or sh*DCAF7* Huh7 xenografted mice. **D**–**G** Images (**D**), growth curves (**E**), tumor weight (**F**), and MDA (**G**) of the sh*NC* or sh*DCAF7*-expressing Huh7 xenografted mice treated as indicated. Mean ± SD (n = 6 mice per group). **H** and **I** The relative viability of HCC cells treated with the indicated concentrations of ML364 and sorafenib for 24 h. Mean ± SD (n = 3, biological replicates). **J** and **K** The relative viability of HCC cells treated with the indicated concentrations of ML364 and sorafenib (20 μM for Huh7, 10 μM for SNU-449) in the absence or presence of Fer-1 (1 μM) for 24 h. Mean ± SD (n = 3, biological replicates). **L** and **M** The OD_450_ of the HCC cells treated with ML364 (2 μM), sorafenib (2 μM), or the combination treatment of sorafenib (2 μM) and ML364 (2 μM) for different durations. Mean ± SD (n = 3, biological replicates). **N** Schematic representation of the therapy schedule of sorafenib, ML364, or combination therapy for Huh7 xenografted mice. **O**–**R** Images (**O**), growth curves (**P**), tumor weight (**Q**), and MDA (**R**) of the Huh7 xenografted mice treated as indicated. Mean ± SD (n = 6 mice per group). The *P*-values were calculated using one-way ANOVA analysis with a Tukey’s multiple comparisons post hoc test (**F–K**, **Q**, and **R**), and two-way ANOVA analysis with a Sidak’s multiple comparisons post hoc test (**A**, **B**, **E**, **L**, **M**, and **P**). NS, *P* > 0.05, *P < 0.05, **P < 0.01, ***P < 0.001, ****P < 0.0001.

Sorafenib, a multiple tyrosine kinase inhibitor, has been approved by the FDA as first-line therapy for advanced HCC [30]. As DCAF7 has been implicated in regulating drug sensitivity in pancreatic neuroendocrine tumors and nasopharyngeal carcinoma [1, 2], we sought to investigate its potential involvement in modulating sorafenib response in HCC. CCK-8 assay disclosed that *DCAF7*-knockdown sensitized HCC cells to sorafenib significantly (Fig. 7A, B), supporting that DCAF7 is a critical determinant of sorafenib efficacy. Indeed, *DCAF7* knockdown significantly enhanced sorafenib sensitivity and dramatically suppressed Huh7 xenograft tumor growth in vivo, without altering the mouse body weight (Fig. 7C–F and Fig. S7A). Notably, these antitumor and synergistic effects were partially rescued by the ferroptosis inhibitor Fer-1 (Fig. 7D–F). Consistent with these results, *DCAF7* knockdown increased the MDA level, especially in the presence of sorafenib, while Fer-1 substantially mitigated the upregulation of lipid peroxidation under these conditions (Fig. 7G), suggesting *DCAF7* knockdown potentiates sorafenib response through ferroptosis in HCC. In addition, *DCAF7* knockdown diminished BMAL1 protein level without altering its mRNA level, and attenuated the mRNA and protein level of HIF1α and SLC7A11, but did not influence the expression of USP2 in Huh7 xenografts (Fig. S7B, C).

Our aforementioned results demonstrated that USP2 collaborates with DCAF7 to regulate clockophagy-induced ferroptosis. This evidence encouraged us to investigate the function of USP2 on sorafenib response in HCC cells. As expected, ML364 substantially sensitized HCC cells to sorafenib (Fig. 7H, I), while this process was effectively abrogated by Fer-1 (Fig. 7J, K). Additionally, ML364 significantly augmented sorafenib’s anti-proliferative activity in both Huh7 and the sorafenib-insensitive SMMC-7721 cells (Fig. 7L, M). Moreover, we evaluated the therapeutic potential of combining ML364 with sorafenib in vivo (Fig. 7N). As expected, the combinatory therapy significantly reduced the tumor volume and weight (Fig. 7O–Q), with obviously increased MDA level in Huh7 xenografts (Fig. 7R), further supporting the involvement of ferroptosis in this synergistic effect. It should be noted that these treatments did not alter animal body weight or hematologic indices (Fig. S7D, E), suggesting the treatment was well-tolerated. These findings align with and extend prior safety data for ML364 [31], further supporting its therapeutic potential. Moreover, the combinatory therapy reduced the transcription of *HIF1α* and *SLC7A11*, as well as reduced the protein level of DCAF7, BMAL1, HIF1α, and SLC7A11, but did not affect the USP2 protein level (Fig. S7F, G). Consistently, IHC analysis further demonstrated that the combinatory treatment effectively suppressed BMAL1 expression while upregulating the ferroptosis marker PTGS2 (Fig. S7H).

Collectively, our data substantiate that targeting either DCAF7 or USP2 induces ferroptosis to potentiate sorafenib sensitivity through ferroptosis in vitro and in vivo and highlight the combination of sorafenib and USP2 inhibitors as a promising strategy for improving clinical outcomes in HCC patients.

### Clinical relevance of the DCAF7/USP2/BMAL1-HIF1α axis in HCC

**Fig. 8:**
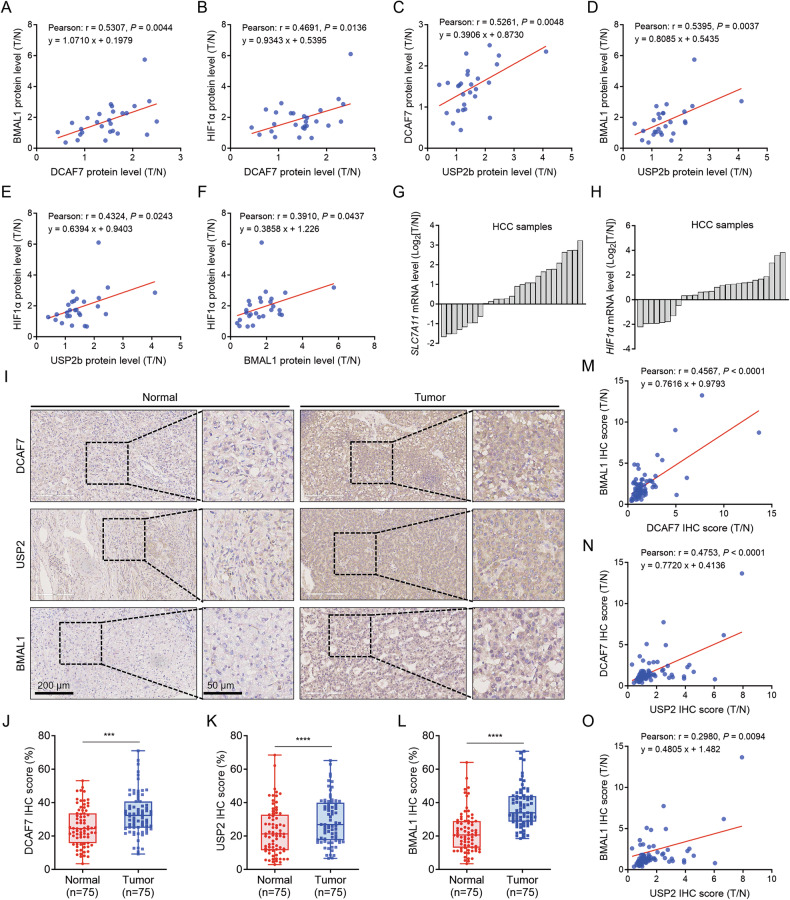
Clinical relevance of the DCAF7/USP2/BMAL1-HIF1α-SLC7A11 axis in HCC. **A**–**F** The two-tailed Pearson correlation analyses of the abundance of the indicated proteins in HCC tissue samples. **G**–**H** Waterfall plot of the relative *SLC7A11* (**G**) and *HIF1α* (**H**) mRNA level measured by qPCR from 27 HCC and paired adjacent tissues. Each bar represents one case. **I** Representative images for DCAF7, USP2, and BMAL1 staining in human HCC tissue microarray samples. Scale bars represent 200 μm and 50 μm. **J**–**L** The staining scores for the indicated proteins in a tissue microarray with 75 paired HCC and matched adjacent tissues were compared. **M**–**O** The two-tailed Pearson correlation analyses of the staining scores of indicated proteins with a tissue microarray containing 75 paired HCC clinical tissue specimens. The *P*-values were calculated using a two-tailed, paired Student’s *t*-test (**J**–**L**). ***P < 0.001, ****P < 0.0001.

Next, we aimed to determine the clinical relevance of the DCAF7/USP2/BMAL1-HIF1α axis in HCC patient tissues. On the one hand, Western blotting analysis of 27 paired HCC tissues indicated that DCAF7, USP2a/b, BMAL1, and HIF1α proteins were highly elevated in HCC tissues (Fig. 1D and Fig. S8A–I). Notably, we observed a strong positive correlation between DCAF7 and BMAL1 and HIF1α in HCC specimens (Fig. 8A, B). However, only USP2b demonstrated positive correlations with DCAF7, BMAL1, and HIF1α (Fig. 8C–E and Fig. S8J–L). Furthermore, BMAL1 protein expression showed a positive correlation with HIF1α levels (Fig. 8F). On the other hand, qPCR analysis of 27 paired HCC samples revealed that the HCC samples exhibited elevated *HIF1α* and *SLC7A11* mRNA levels, consistent with data from the UALCAN database (Fig. 8G, H and Fig. S8M, N). In addition, our data indicated that *BMAL1* and *USP2* mRNA was elevated in most HCC tissue samples (Fig. S8O, P).

We further verified these findings by a tissue microarray containing 75 paired HCC clinical tissue specimens. Similarly, quantitative analysis revealed significant overexpression of DCAF7, USP2, and BMAL1 proteins in HCC tissues compared to adjacent tissues (Fig. 8I–L). Moreover, correlation analysis demonstrated strong positive associations among DCAF7, USP2, and BMAL1 proteins in HCC samples (Fig. 8M–O). Collectively, these findings underscore the clinical relevance of the DCAF7/USP2/BMAL1-HIF1α axis in HCC pathogenesis.

## Discussion

In this study, we identified DCAF7 as a novel oncogenic factor in HCC. Functional studies revealed that *DCAF7* ablation potently induced ferroptosis, thereby suppressing HCC progression. Mechanistically, we discovered that DCAF7 recruits USP2 to promote BMAL1 deubiquitination and simultaneously disrupts the BMAL1-p62 association, thereby inhibiting clockophagy. *DCAF7* deficiency or USP2 inhibition triggered clockophagy and suppressed the HIF1α-SLC7A11 axis to induce ferroptosis. Importantly, ML364 synergized with sorafenib to augment ferroptosis and suppress tumor growth, underscoring the therapeutic potential for HCC.

Emerging evidence has implicated DCAF7 as an oncogenic driver in various malignancies, including pancreatic neuroendocrine tumors and nasopharyngeal carcinoma [1, 2]. In our work, we evaluated the role of DCAF7 in HCC, uncovered its anti-ferroptosis function, and elucidated its mechanism in regulating HCC progression, raising the possibility of targeting DCAF7 for the combinatory therapy to HCC. Generally, DCAF7 acts as a substrate receptor for the CRL4 E3 ligase complex, participating in the ubiquitin-proteasomal degradation of specific substrates [1]. This process requires the formation of the CUL4-DDB1 complexes and the neddylation-mediated CRL4 activation. However, our aforementioned data indicated that neither *DDB1* knockdown nor inhibition of the NEDD8-activating enzyme impedes the DCAF7-mediated upregulation of BMAL1, suggesting that DCAF7 stabilizes BMAL1 independently of the CRL4 E3 ligase activity. Structurally, DCAF7 is a WD repeat-containing protein, which assembles into a ringed β-propeller structure, providing multiple open binding surfaces for protein-protein interactions [32, 33]. Consistently, our work revealed that DCAF7 served as a scaffold protein to promote the interaction between USP2 and BMAL1 and thus to stabilize BMAL1. This observation coincides with DCAF7 promotes the interaction between USP10 and G3BP1, leading to the deubiquitination of G3BP1 [2]. Thus, these results highlight the crucial dual roles of DCAF7 in modulating substrate ubiquitination, either as a substrate receptor for the CRL4 E3 ligase complex directly mediating substrate ubiquitination or as a scaffold protein bridging substrate to its specific deubiquitinase.

Previous studies have demonstrated that DCAF7 can regulate apoptosis and other pathways to inhibit tumor cell growth [1, 2]. In our study, while ferroptosis inhibitors partially but significantly rescued the growth-inhibitory effects of *DCAF7* knockdown in HCC cells both in vitro and in vivo, the incomplete rescue implicates coexisting mechanisms involving both alternative cell death pathways and cell death-independent growth inhibition. These findings highlight the multifaceted nature of DCAF7’s tumor-suppressive functions, with ferroptosis representing one of the important mechanisms. Further investigation will be needed to fully dissect the complete spectrum of growth-inhibitory pathways regulated by DCAF7 in HCC.

Autophagy is a highly conserved lysosome-dependent degradation of cellular proteins or organelles [34, 35]. Recent studies have discovered a series of autophagy-dependent ferroptosis, such as ferritinophagy [36], lipophagy [37], and clockophagy [9]. Clockophagy, the selective autophagic degradation of BMAL1 by p62 in response to the ferroptosis inducer RSL3, is critical for ferroptosis in ferroptosis-sensitive cancer cell lines [9]. In our work, we further explore the role and the precise regulatory mechanism of clockophagy in HCC progression. Specifically, we demonstrated that DCAF7 recruits USP2 to BMAL1, which promotes the deubiquitination of BMAL1 and diminishes the BMAL1-p62 interaction. This process reduces the autophagic degradation of BMAL1 and stabilizes this protein. Consequently, targeting either DCAF7 or USP2 promotes clockophagy to induce ferroptosis. We further uncovered that *DCAF7* knockdown inhibits *HIF1a* transcription through BMAL1, in line with the previous discovery that BMAL1 directly activates *HIF1α* transcription [23]. In addition, it has been reported that *HIF1α* and *SLC7A11* are highly expressed, which inhibits ferroptosis in sorafenib-resistant HCC cells and tissues [16]. Our work further demonstrated that *DCAF7* knockdown sensitized HCC cells to sorafenib by inducing ferroptosis. Thus, this study not only broadens our understanding of clockophagy in HCC but also uncovers novel therapeutic strategies for HCC through targeting clockophagy.

USP2 was generally thought to stabilize BMAL1 by inhibiting its ubiquitin-mediated proteasomal degradation; however, no biological experiments have directly confirmed this hypothesis [10]. Our work uncovered that both USP2a and USP2b stabilize BMAL1 by inhibiting its autophagic degradation rather than inhibiting its ubiquitin-mediated proteasomal degradation. Specifically, USP2 reduced the K63-linked polyubiquitination on BMAL1 and diminished the BMAL1-p62 interaction to block clockophagy. We further disclosed that this process depended on DCAF7, which functioned as a necessary scaffold protein. Moreover, our work discovered that USP2 stabilized DCAF7 by inhibiting its autophagic degradation through its catalytic activity, while DCAF7 did not significantly affect the protein level of USP2. These findings collectively establish a novel regulatory model for the USP2-DCAF7-BMAL1 protein complex, expanding our understanding of the intricate regulatory networks involved in cellular homeostasis.

Emerging research indicates that USP2 exhibits pro-tumorigenic activity in several tumors [12]. However, a recent work disclosed that *USP2* depletion suppressed ferroptosis by inducing the degradation of NCOA4 to facilitate ESCC tumorigenesis [38]. This apparent contradiction likely reflects the complex, tissue-specific regulation of USP2 function, which may be influenced by several reasons: differential expression patterns of USP2 isoforms (USP2a vs. USP2b), cancer-type-specific availability of USP2 substrates, and variable cellular dependence on distinct USP2-regulated pathways. Our work extends this understanding by identifying a novel mechanism through which USP2 promotes HCC progression via modulating BMAL1 stabilization and subsequent inhibition of clockophagy-mediated ferroptosis. Importantly, our therapeutic investigations revealed that pharmacological inhibition of USP2 with ML364 significantly enhances sorafenib sensitivity in HCC cells. These findings not only expand our understanding of USP2’s role in regulating ferroptosis but also have important clinical implications for overcoming sorafenib resistance in HCC. Further studies are necessary to investigate whether the combination of a USP2 inhibitor and sorafenib is particularly effective for HCC patients in clinical trials.

In this study, we reveal that DCAF7 and USP2 are two previously unrecognized negative regulators of clockophagy-induced ferroptosis in HCC progression and elucidate the associated molecular mechanisms. These novel findings highlight the therapeutic potential of targeting the USP2-DCAF7-BMAL1 protein complex as a novel and promising therapeutic strategy for HCC treatment.

## Supplementary information

Supplementary Materials

Uncropped blots

## Data Availability

All data are available in the main text or the supplementary materials. The mass spectrometry proteomics data have been deposited to the ProteomeXchange Consortium (https://proteomecentral.proteomexchange.org, RRID: SCR_004055) via the iProX (RRID: SCR_021741) partner repository [39, 40] with the dataset identifier PXD056709.
