# Supplementary material for: DCAF7 recruits USP2 to facilitate hepatocellular carcinoma progression by suppressing clockophagy-induced ferroptosis
Source: Cell Death Dis. 2025 Aug 28;16(1):654. doi: 10.1038/s41419-025-07977-3 (PMC12394690; doi:10.1038/s41419-025-07977-3)
Supplement: Supplementary file 1 — Supplementary Materials [file 41419_2025_7977_MOESM1_ESM.pdf]

DCAF7 recruits USP2 to facilitate hepatocellular carcinoma progression by suppressing clockophagy-induced ferroptosis

Honglv Jiang, Xiaohui Wang, Zhenhua Zhu, Cheng Song, Dan Li, Yixuan Yun, Li Hui, Leilei Bao, Darran P. O'Connor, Jingjing Ma, Guoqiang Xu

This PDF file includes:

Supplementary Methods

Fig. S1. DCAF7 is highly expressed in HCC and promotes HCC progression.
Fig. S2. *DCAF7* deficiency induces ferroptosis to suppress HCC progression through the HIF1α-SLC7A11 axis.
Fig. S3. DCAF7 stabilizes BMAL1 to upregulate *HIF1α* transcription.
Fig. S4. USP2 deubiquitinates BMAL1 and inhibits clockophagy.
Fig. S5. DCAF7 recruits USP2 to deubiquitinate BMAL1 and inhibit clockophagy.
Fig. S6. USP2 inhibits DCAF7 autophagic degradation and modulates ferroptosis.
Fig. S7. Targeting DCAF7 or USP2 sensitizes HCC cells to sorafenib by inducing ferroptosis.
Fig. S8. Clinical relevance of the DCAF7/USP2/BMAL1-HIF1α-SLC7A11 axis in HCC.

Table S1. Information of antibodies used in this work.
Table S2. Sequences for primers used for qRT-PCR.
Table S3. siRNA sequences used in this work.
Table S4. Sequences for primers used for plasmid construction.
Table S5. shRNA sequences for the construction of shRNA plasmids.

Supplementary Methods

Plasmid construction

The primers (Table S4) for plasmid construction were synthesized by GENEWIZ. The open reading frames of human (h) DCAF7, USP2a, and USP2b were amplified from the cDNA library obtained from HEK293T cells by RT-PCR with the appropriate epitope tags and then cloned into the pCDH or pcDNA3.1 vector. The USP2b mutant plasmid USP2b^{C67A} was constructed using the Mut Express MultiS Fast Mutagenesis Kit (C215-01, Vazyme) following the manufacturer's instructions. FLAG-BMAL1, HA-HIF1α, and wild-type (WT), K48R, and K63R HA-ubiquitin plasmids were kind gifts from Dr. Ying Xu, Dr. Yanli Liu, and Dr. Xinliang Mao, respectively, at Soochow University.

Construction of stable cell lines

Stable *DCAF7*- and *p62*-knockdown cell lines were constructed according to previously described methods. Briefly, the shRNA sequences targeting *DCAF7* or *p62* (Table S5) were synthesized by GENEWIZ and inserted into the pLKO.1-TRC vector. For the production of lentiviral particles, HEK293T packaging cells were transfected with 20 μg of lentiviral vectors, including 10 μg targeting shRNA or empty vector, 6.6 μg psPAX2, and 3.4 μg pMD2.G using PEI transfection reagent. At 48 h after transfection, the supernatant containing viral particles was collected and filtered with a 0.45 μm filter. HCC or HEK293T cells were treated with the supernatant and supplemented with 10 μg/mL polybrene for 12 h. The stable cell lines were selected with puromycin (2 μg/mL) for two weeks. The knockdown efficiency of target genes was examined by qPCR and Western blotting.

Immunofluorescence

Plasmid-transfected cells in 24-well plates were processed 48 h post-transfection. After PBS rinsing, fixation (4% PFA, 10 min), and permeabilization (0.1% Triton X-100, 5 min), samples were blocked with 5% BSA (2 h). Overnight incubation with primary antibodies (1:200) at 4°C was followed by TBST washes and staining with fluorescent secondary antibodies (1:500, 2 h, dark). DAPI (1:10,000) was used for nuclear labeling before confocal imaging (Nikon A1R HD25).

Chromatin immunoprecipitation (ChIP)-qPCR

HepG2 cells (5×10^6) were transfected with either pcDNA3.1 (empty vector) or FLAG-BMAL1 plasmids for 48 hours. Cells were then fixed with 1% formaldehyde for 10 min at room temperature. After fixation, cells were collected, lysed, and subjected to sonication (10 cycles of 10 sec ON / 20 sec OFF, 50% amplitude) using a Sonics VCX130 ultrasonic processor (Sonics & Materials, USA) to shear chromatin into fragments of 200–500 bp. BMAL1-DNA complexes were immunoprecipitated using anti-FLAG affinity resin. The enriched DNA was amplified by qPCR using ChamQ SYBR qPCR Master Mix (Vazyme, Q511-02). The primers (forward: 5′-TGTTTGGGACCAGGCAACC-3′; reverse: 5′-TGAGGTGGAGGCGGGTT-3′) targeted the E-box motif (GCACGTGA) in the *HIF1α* promoter region (- 371 to - 363 bp).

GST pull-down assay

GST-His_6-DCAF7 and MBP-His_6-USP2 (USP2a/USP2b) plasmids were expressed in *E. coli*, and fusion proteins were purified following standard protocols. For pull-down assays, ~100 µg of GST or GST-His_6-DCAF7 was immobilized on GST-tag purification resin (Beyotime, P2251) in 50 µL suspension, equilibrated, and incubated with ~100 µg MBP-His_6-USP2 at 4°C for 1 h with gentle rotation. After three PBST washes, the protein complexes were incubated overnight at 4°C. Bound proteins were eluted using glutathione buffer (10 mM in PBS, pH 8.0) and detected via Coomassie blue staining and Western blotting.

Mass spectrometry analysis

Following SDS-PAGE separation of control and FLAG-DCAF7 immunoprecipitated samples, gel bands were manually excised and diced into 1 mm^3 pieces. After standard in-gel reduction, alkylation, and tryptic digestion, peptides were extracted, desalted using C18 ZipTips, and reconstituted in 0.1% formic acid. LC-MS/MS analysis was performed on a Q Exactive Orbitrap mass spectrometer (Thermo Fisher Scientific) coupled to a nanoHPLC system. Peptides were separated on a C18 column using a 120-min gradient (4-99% B; solvent A: 0.1% FA, solvent B: 80% ACN/0.1% FA) and analyzed in positive ion mode with data-dependent acquisition

A Q Exactive Orbitrap mass spectrometer with an electrospray ionization inlet (Thermo Fisher Scientific, RRID: SCR_014593) was used to analyze the peptide samples. Briefly, samples were loaded on a C18 analytical column through a nanoscale HPLC with 96% solvent A

(0.1% formic acid) and 4% solvent B (80% acetonitrile and 0.1% formic acid). Peptides were separated with a 120 min segmented gradient as follows: 4–5% solvent B for 2 min, 5–20% solvent B for 88 min, 20–35% solvent B for 27 min, 35–99% solvent B for 1 min, followed by a 4 min 100% solvent B wash. The Q Exactive Orbitrap was operated in the positive ion mode with a data-dependent acquisition system. The instrument parameters were set as follows: MS scan range (m/z): 350-1550; maximum injection time: 50 ms; AGC target: 3.0×10^6; microscan: 1; resolution 70,000 (at 200 m/z); isolation window for MS/MS: 2 m/z; loop count: 15; normalized collision energies (nce): 30%.

MS data processing

MS/MS raw data were processed using MaxQuant (version 2.6.7.0, RRID: SCR_014485) with the human UniProt database (www.uniprot.org), including reversed sequences and common contaminants. Trypsin was specified as the protease, permitting up to 2 missed cleavages. Carbamidomethylation of cysteine residues was defined as a fixed modification, while methionine oxidation and N-terminal acetylation were variable modifications. The mass error thresholds were set to 10 ppm (precursor ions) and 0.6 Da (fragment ions), with a 1% FDR cutoff at the peptide level.

For relative protein quantification, the iBAQ values from MaxQuant were utilized. Missing iBAQ values were imputed using Perseus (version 2.1.3.0, RRID: SCR_015753) by generating random numbers from a normal distribution (width: 0.3; downshift: 1.8). Statistical significance was assessed via a two-tailed Student's *t*-test. Volcano plots were generated in GraphPad Prism by plotting Log_2 ($iBAQ_{DCAF7}/iBAQ_{pcDNA3.1}$) against $-Log_{10}$ (P-value) based on triplicate experiments.

Supplementary Figures

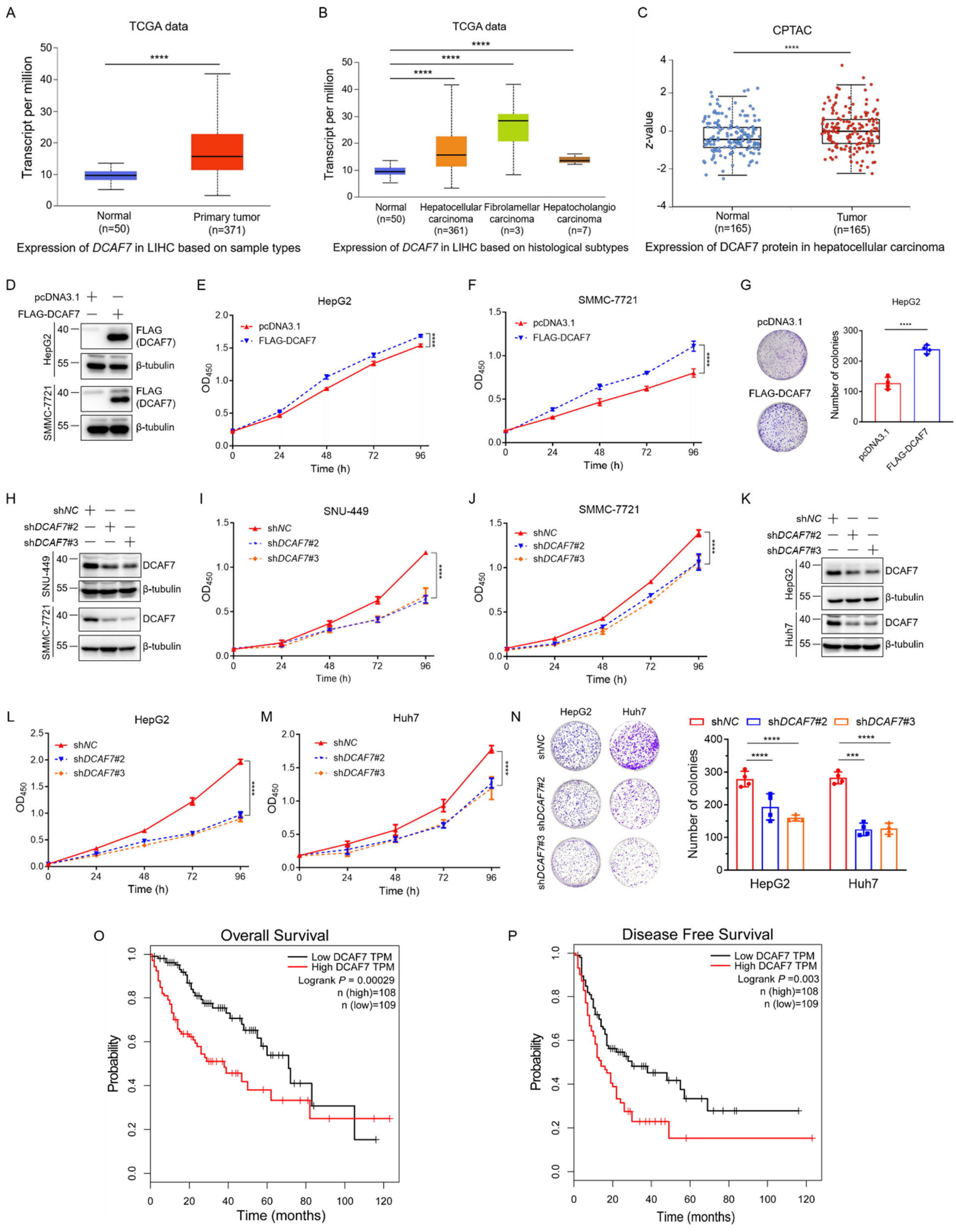

Fig. S1. DCAF7 is highly expressed in HCC and promotes HCC progression. (**A** and **B**) The expression of *DCAF7* mRNA in normal or liver cancer (tumor) samples with different sample types and histological subtypes. Data were obtained from the UALCAN database (https://ualcan.path.uab.edu/index.html). (**C**) The protein level of DCAF7 in normal and HCC (tumor) tissues obtained from the CPTAC database (https://proteomics.cancer.gov). (**D**) Western blotting analysis of cell lysates from HepG2 and SMMC-7721 cells transfected with control or FLAG-DCAF7 plasmids. (**E** and **F**) The OD_{450} of HepG2 (**E**) and SMMC-7721 (**F**) cells transfected with either an empty vector or FLAG-DCAF7 plasmid. Mean ± SD (n = 3, biological replicates). (**G**) Colony formation assays of HepG2 cells transfected with either an empty vector or FLAG-DCAF7 plasmid. Mean ± SD (n = 4, biological replicates). (**H**) Western blotting analysis of the *DCAF7* knockdown efficiency in SNU-449 and SMMC-7721 cells. (**I** and **J**) The OD_{450} of the SNU-449 (**I**) and SMMC-7721 (**J**) cells stably transfected with sh*NC* or sh*DCAF7*. Mean ± SD (n = 3, biological replicates). (**K**) Western blotting analysis of the *DCAF7* knockdown efficiency in HepG2 and Huh7 cells. (**L** and **M**) The OD_{450} of HepG2 (**L**) and Huh7 (**M**) cells stably transfected with sh*NC* or sh*DCAF7*. Mean ± SD (n = 3, biological replicates). (**N**) Colony formation assays of HepG2 and Huh7 cells transfected with sh*NC* or sh*DCAF7*. Mean ± SD (n = 4, biological replicates). (**O** and **P**) The overall survival (OS) and disease-free survival (DFS) analysis of HCC patients with different expression levels of *DCAF7* mRNA. The data were obtained from the GPEIA database (http://gepia.cancer-pku.cn/). The *P* values were calculated using two-tailed, unpaired Student's *t*-test (**A** to **C**, **G**, and **N**) and two-way ANOVA analysis with a Sidak's multiple comparisons post-test (**E**, **F**, **I, J, L,** and **M**). $***P < 0.001$, $****P < 0.0001$.

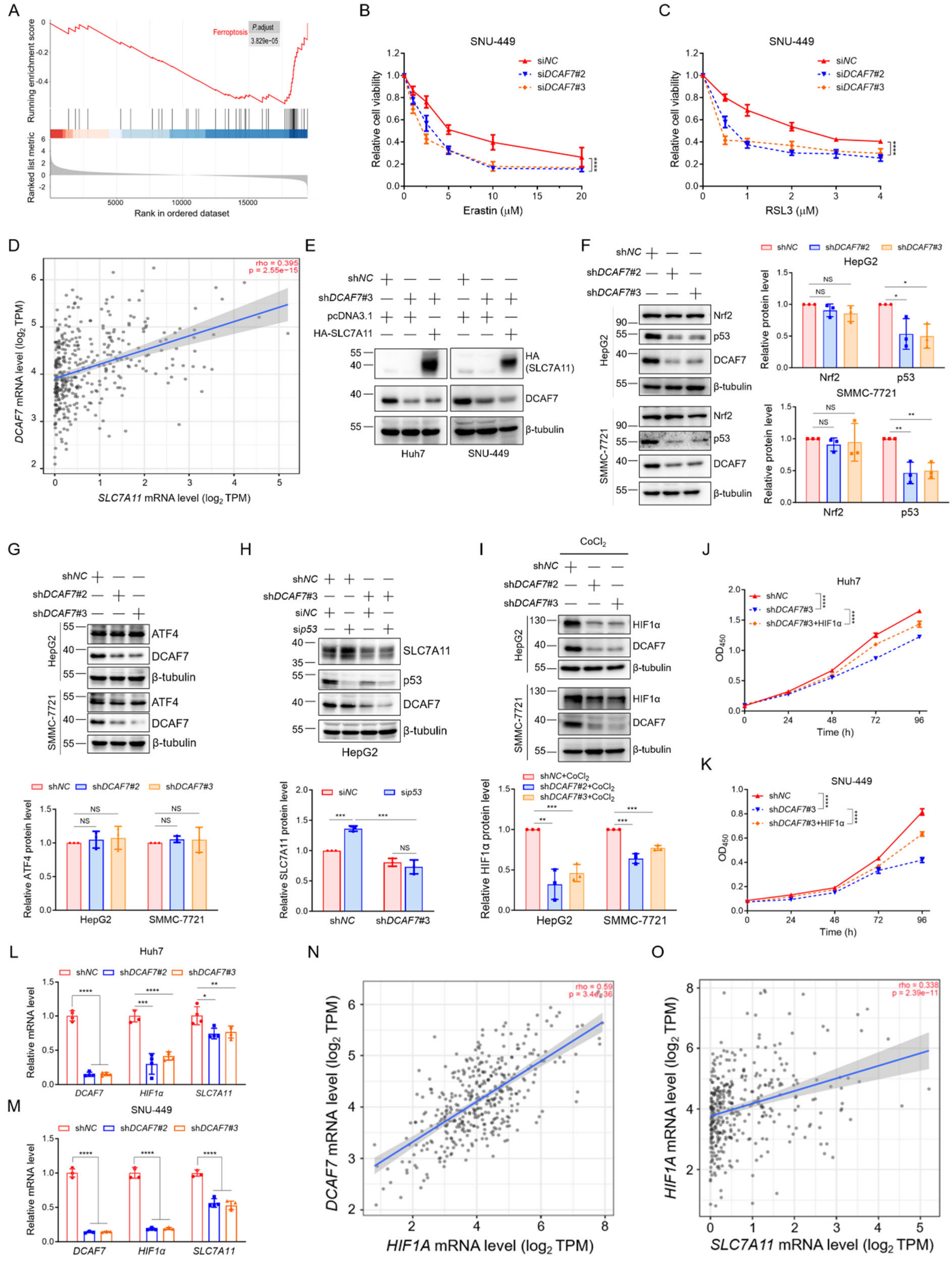

Fig. S2. *DCAF7* deficiency induces ferroptosis to suppress HCC progression through the HIF1α-SLC7A11 axis. (A) Enrichment of DCAF7 in the ferroptosis pathway by GSEA. The

enriched gene sets in ferroptosis ranked by *DCAF7* expression. (**B** and **C**) The relative cell viability of si*NC* or si*DCAF7* transfected SUN-449 cells treated with different concentrations of the ferroptosis inducer erastin (**B**) or RSL3 (**C**) for 24 h. Mean ± SD (n = 3, biological replicates). (**D**) Correlation analysis of the mRNA expression of DCAF7 and SLC7A11 in LIHC obtained from the TCGA database (http://timer.cistrome.org). (**E**) Western blotting analysis of cell lysates from the control and *DCAF7*-knockdown Huh7 and SNU-449 cells transfected with either empty vector or HA-SLC7A11 plasmid. (**F-G**) Western blotting analysis and quantification of Nrf2, p53 (**F**), and ATF4 (**G**) in the control or *DCAF7*-knockdown HCC cells. Means ± SD (n = 3, biological replicates). (**H**) Western blotting analysis and quantification of the effect of *DCAF7*-knockdown on SLC7A11 protein level in the control or *p53*-knockdown HepG2 cells. Mean ± SD (n = 3, biological replicates). (**I**) Western blotting analysis and quantification of HIF1α in $CoCl_2$-treated control or *DCAF7*-knockdown HepG2 and SMMC-7721 cells ($CoCl_2$: 500 μM, 12 h). Mean ± SD (n = 3, biological replicates). (**J-K**) The OD_{450} of the control or *DCAF7*-knockdown HCC cells transfected with an empty vector or HA-HIF1α plasmid. Mean ± SD (n = 3, biological replicates). (**L-M**) qPCR analysis of the relative mRNA level of *HIF1α* and its downstream *SLC7A11* in the control or *DCAF7*-knockdown HCC cells. Mean ± SD (n = 3, biological replicates). (**N-O**) Correlation analysis of the mRNA level of *DCAF7-HIF1α* (**N**) and *HIF1α-SLC7A11* (**O**) in LIHC obtained from the TCGA database. TPM, transcripts per million. The *P* values were calculated using two-tailed, unpaired Student's *t*-test (**F-I, L** and **M**), and two-way ANOVA analysis with a Sidak's multiple comparisons post-test (**B**, **C**, **J**, and **K**). NS, $P > 0.05$, *$P < 0.05$, **$P < 0.01$, ***$P < 0.001$, ****$P < 0.0001$.

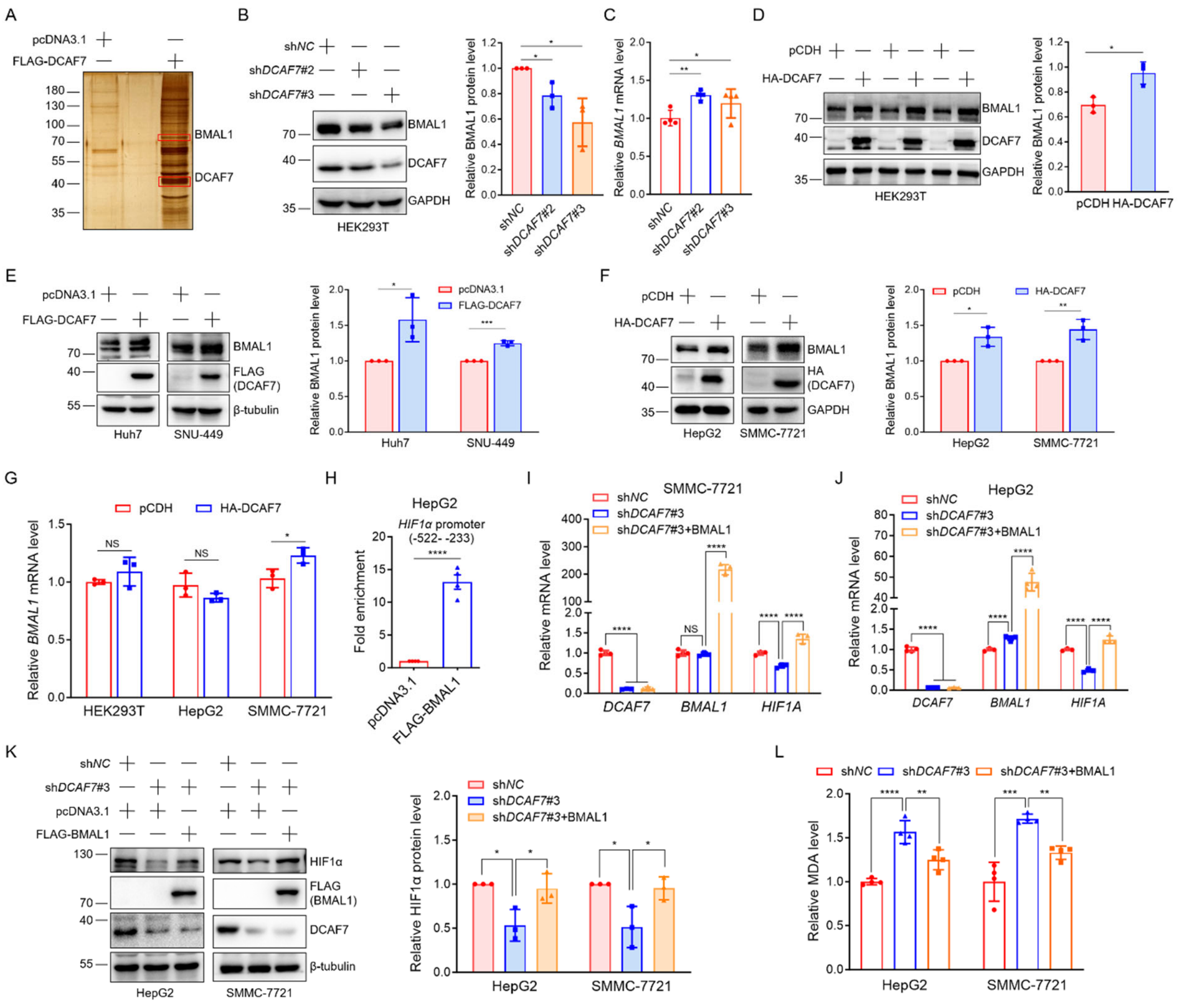

Fig. S3. DCAF7 stabilizes BMAL1 to upregulate *HIF1α* transcription. (**A**) The silver staining results of the FLAG affinity gel purified proteins. (**B** and **C**) Western blotting (**B**) and qPCR (**C**) analysis and quantification of BMAL1 in the control or *DCAF7*-knockdown HEK293T cells. Mean ± SD (n = 3, biological replicates). (**D-G**) Western blotting analysis of BMAL1 protein level (**D** to **F**) and qPCR analysis of the relative *BMAL1* mRNA level (**G**) in the control or DCAF7-overexpressed HEK293T and HCC cells. Mean ± SD (n = 3, biological replicates). (**H**) ChIP-qPCR analysis of the binding of BMAL1 to the E-box-containing promoters of *HIF1α* in BMAL1-overexpressed and control HepG2 cells. (**I-K**) qPCR analysis of the relative *HIF1α* mRNA level (**I** and **J**) and Western blotting analysis of HIF1α (**K**) in the control or *DCAF7*-knockdown HepG2 and SMMC-7721 cells transfected with an empty vector or FLAG-BMAL1 plasmid. Mean ± SD (n = 3, biological replicates). (**L**) The relative MDA level for the control and *DCAF7*-knockdown HepG2 and SMMC-7721 cells transfected with an

empty vector or FLAG-BMAL1 plasmid. Mean ± SD (n = 3, biological replicates). The *P* values were calculated using two-tailed, unpaired Student's *t*-test (**B**-**H**) and one-way ANOVA analysis with a Tukey's multiple comparisons test (**I**-**L**). NS, $P > 0.05$, *$P < 0.05$, **$P < 0.01$, ***$P < 0.001$, ****$P < 0.0001$.

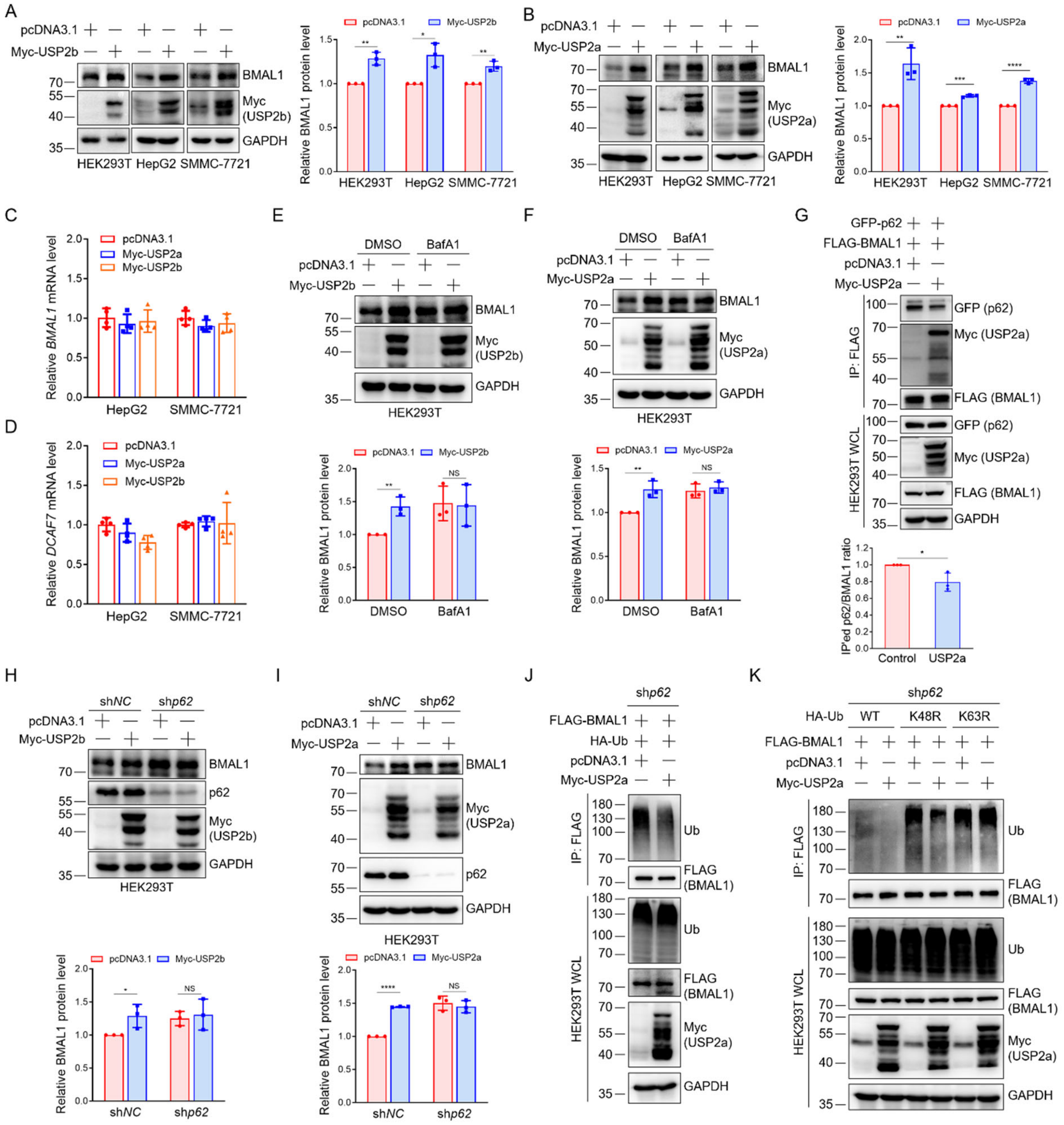

Fig. S4. USP2 deubiquitinates BMAL1 and inhibits clockophagy. (**A** and **B**) Western blotting analysis and quantification of BMAL1 in the control or USP2b (**A**) or USP2a (**B**) overexpressed HEK293T and HCC cells. Mean ± SD (n = 3, biological replicates). (**C** and **D**) qPCR analysis of the relative *BMAL1* (**C**) and *DCAF7* (**D**) mRNA levels in the control or USP2-overexpressed HCC cells. Mean ± SD (n = 3, biological replicates). (**E-F**) Western blotting analysis and quantification of BMAL1 in HEK293T cells transfected with empty vector, Myc-USP2b (**E**) or Myc-USP2a (**F**) plasmid and treated with DMSO or BafA1 (100 nM) for 12 h. Mean ± SD (n =

3, biological replicates). (**G**) Analysis and quantification of the BMAL1-p62 interaction in the absence or presence of USP2a. Mean ± SD (n = 3, biological replicates). (**H-I**) Western blotting analysis and quantification of the effect of USP2b (**H**) or USP2a (**I**) on BMAL1 protein level in the control or *p62*-knockdown HEK293T cells. Mean ± SD (n = 3, biological replicates). (**J**) Analysis of the BMAL1 ubiquitination in the absence or presence of USP2a in *p62*-knockdown HEK293T cells. (**K**) Analysis of the effect of USP2a on the type of polyubiquitin chain on BMAL1 in the *p62*-knockdown HEK293T cells. The *P* values were calculated using two-tailed, unpaired Student's *t*-test (**A-I**). NS, $P > 0.05$, *$P < 0.05$, **$P < 0.01$, ***$P < 0.001$, ****$P < 0.0001$.

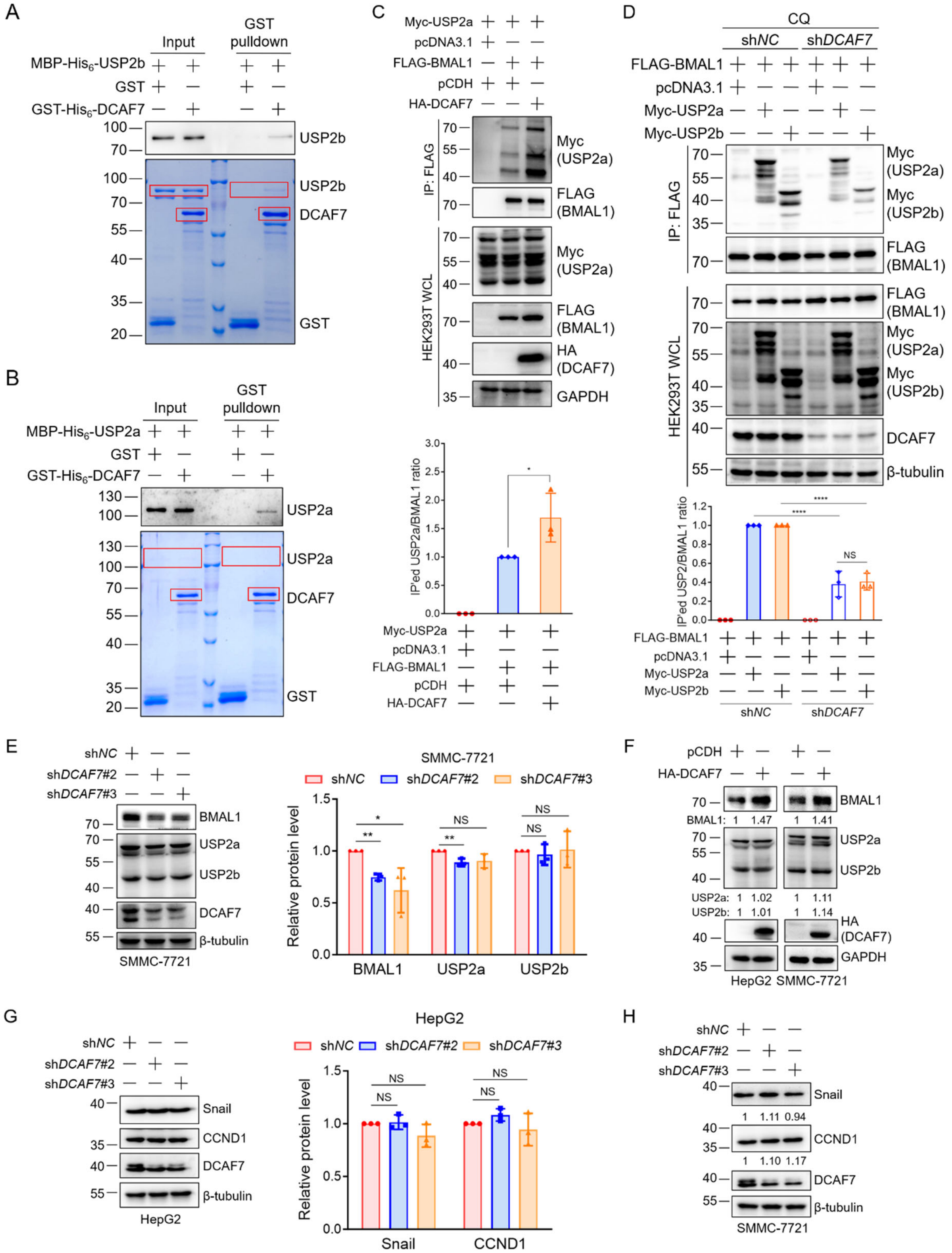

Fig. S5. DCAF7 recruits USP2 to deubiquitinate BMAL1 and inhibit clockophagy. (**A** and **B**) Detection of MBP-His6-USP2b (**A**) or MBP-His6-USP2a (**B**) bound to GST-His6-DCAF7 or

GST in a GST pulldown assay. The results were detected by Coomassie blue staining (down) and Western blotting (top). (**C**) Analysis and quantification of the BMAL1-USP2a interaction in the absence or presence of DCAF7. Mean ± SD (n = 3, biological replicates). (**D**) Analysis and quantification of the BMAL1-USP2a and BMAL1-USP2b interaction in the control or *DCAF7* knockdown HEK293T cells treated with CQ (50 μM) for 12 h. Mean ± SD (n = 3, biological replicates). (**E** and **F**) Western blotting analysis and quantification of the effect of *DCAF7*-knockdown (**E**) or DCAF7 overexpression (**F**) on USP2 protein level in HCC cells. Mean ± SD (n = 3, biological replicates). (**G** and **H**) Western blotting analysis of the effect of *DCAF7*-knockdown on CCND1 and Snail protein level in HepG2 (**G**) and SMMC-7721 (**H**) cells. Mean ± SD (n = 3, biological replicates). The *P* values were calculated using two-tailed, unpaired Student's *t*-test (**C**, **D, E,** and **G**). NS, $P > 0.05$, *$P < 0.05$, **$P < 0.01$, ****$P < 0.0001$.

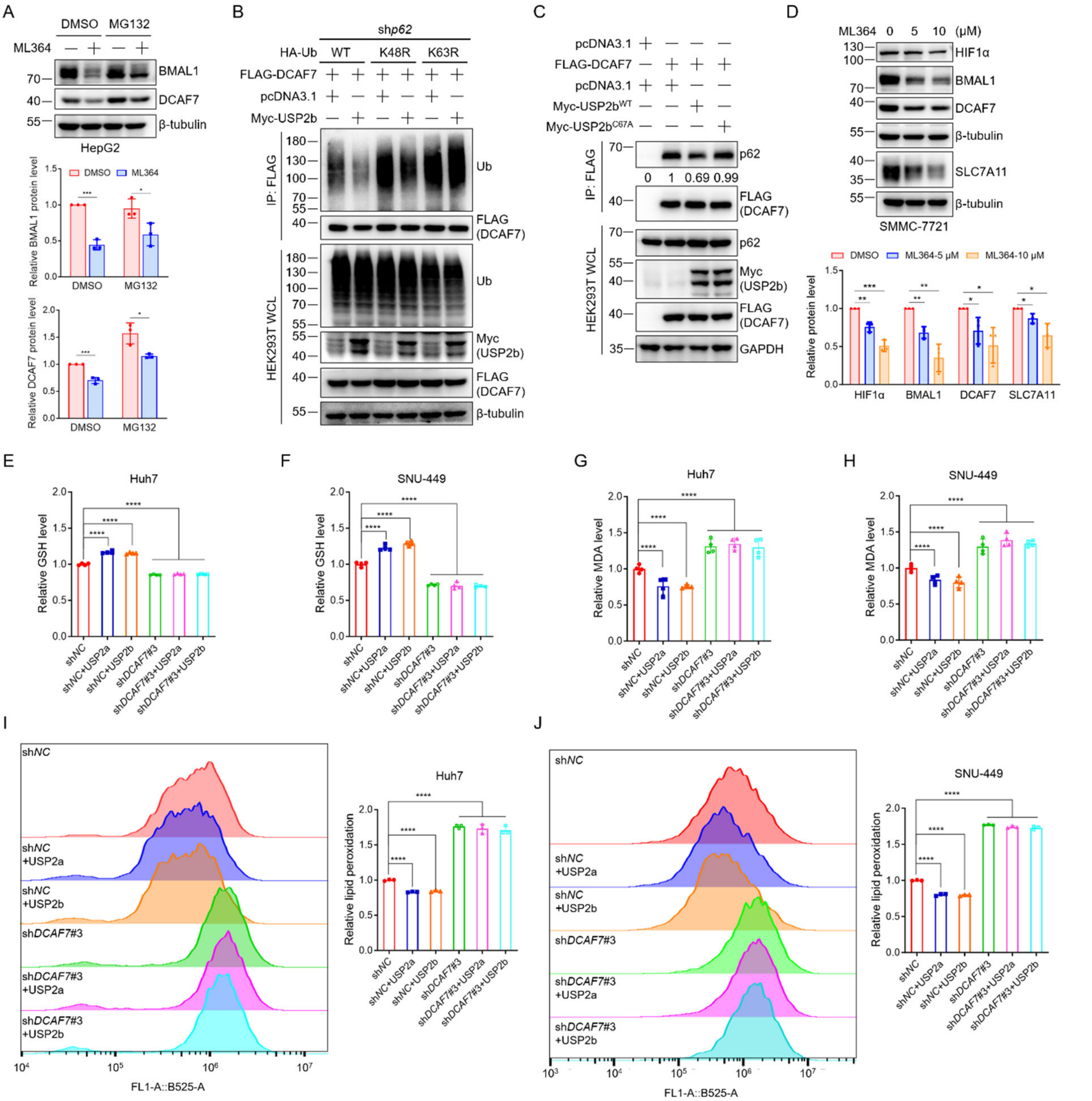

Fig. S6. USP2 inhibits DCAF7 autophagic degradation and modulates ferroptosis. (**A**) Western blotting analysis and quantification of DCAF7 in HepG2 cells treated with DMSO or ML364 (10 μM, 12 h) in the absence or presence of MG132 (10 μM). Mean ± SD (n = 3, biological replicates). (**B**) Analysis of the effect of USP2b on the type of polyubiquitin chain on BMAL1 in the *p62*-knockdown HEK293T cells. (**C**) Analysis of the DCAF7-p62 interaction in the absence or presence of USP2b^{WT} or its catalytically inactive mutant USP2b^{C67A}. (**D**) Western blotting analysis and quantification of the effect of ML364 on DCAF7, BMAL1, HIF1α, and SLC7A11 protein levels in SMMC-7721 cells. Cells were treated with the indicated

concentrations of ML364 for 12 h and lysed for immunoblotting. Mean ± SD (n = 3, biological replicates). (**E-J**) The relative intracellular GSH (**E**, **F**), MDA (**G**, **H**), and ROS (**I**, **J**) levels in the control or *DCAF7*-knockdown HCC cells transfected with an empty vector or Myc-USP2a or USP2b plasmid. Mean ± SD (n = 3, biological replicates). The P values were calculated using two-tailed, unpaired Student's t-test (**A**, **D**, and **E-J**). *$P < 0.05$, **$P < 0.01$, ***$P < 0.001$, ****$P < 0.0001$.

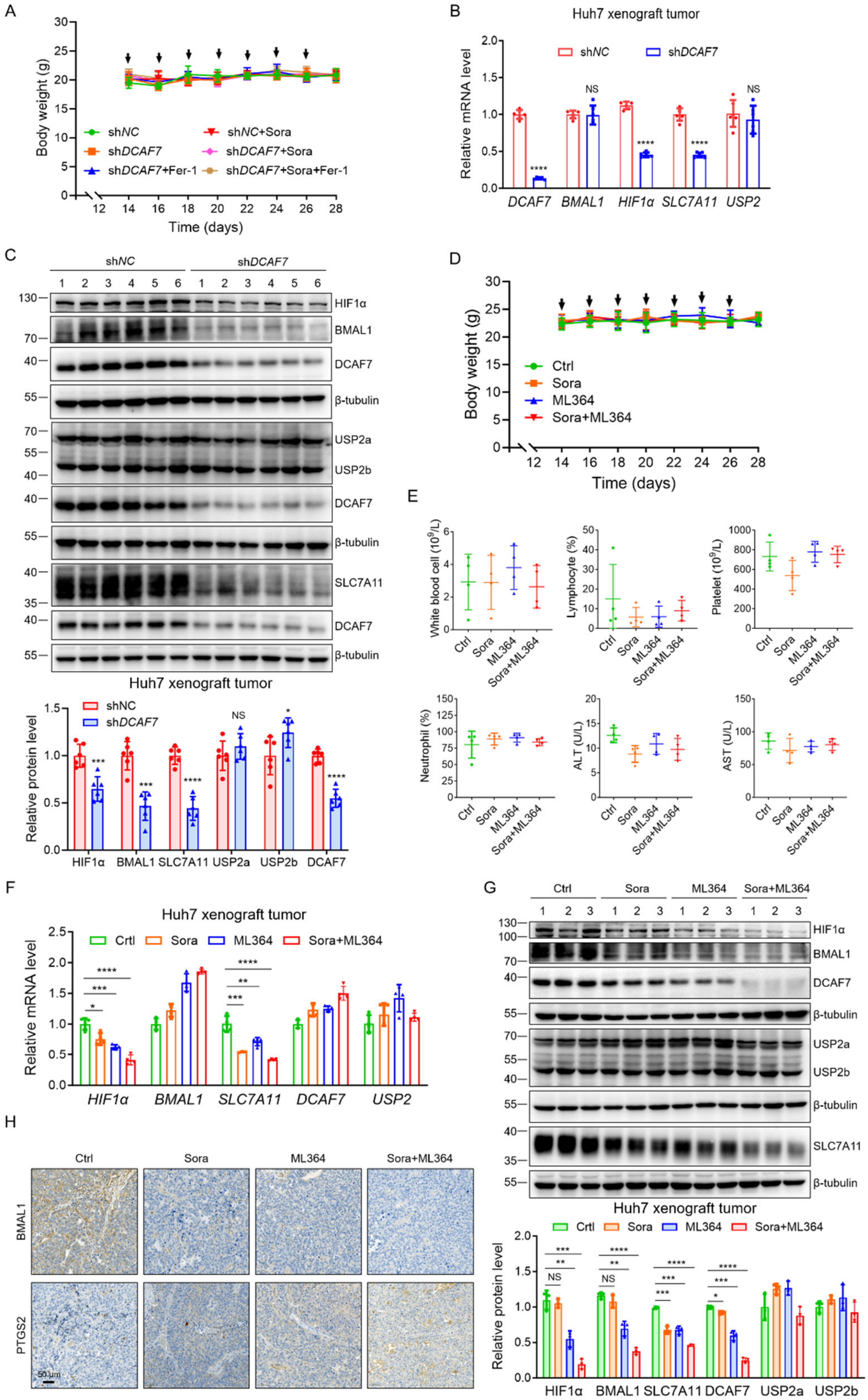

Fig. S7. Targeting DCAF7 or USP2 sensitizes HCC cells to sorafenib by inducing ferroptosis. (**A**) The body weights of nude mice with sh*NC* or sh*DCAF7* Huh7 xenografts

treated as indicated. The black arrow represents the day of administration (n = 6 mice per group). (**B**-**C**) qPCR and Western blotting analysis of the mRNA (**B**) and the protein (**C**) levels of DCAF7, BMAL1, HIF1α, USP2, and SLC7A11 in sh*NC* or sh*DCAF7*-expressing Huh7 xenograft tumors. Mean ± SD (n = 6). (**D**) The body weights of nude mice with Huh7 xenografts treated as indicated. The black arrow represents the day of administration (n = 6 mice per group). (**E**) The hematologic indices of nude mice treated as indicated. Mean ± SD (n = 4). (**F** and **G**) qPCR and Western blotting analysis of the mRNA (**F**) and the protein (**G**) level of DCAF7, BMAL1, HIF1α, USP2, and SLC7A11 in Huh7 xenograft tumors treated as indicated. Mean ± SD (n = 3). (**H**) Representative IHC images for BMAL1 and PTGS2 in Huh7 xenograft tumors treated as indicated (Sora, 20 mg/kg; ML364, 30 mg/kg). Scale bar: 50 μm. The *P* values were calculated using a two-tailed, unpaired Student's *t*-test (**B**, **C**, **F**, and **G**). NS, $P > 0.05$, *$P < 0.05$, **$P < 0.01$, ***$P < 0.001$, ****$P < 0.0001$.

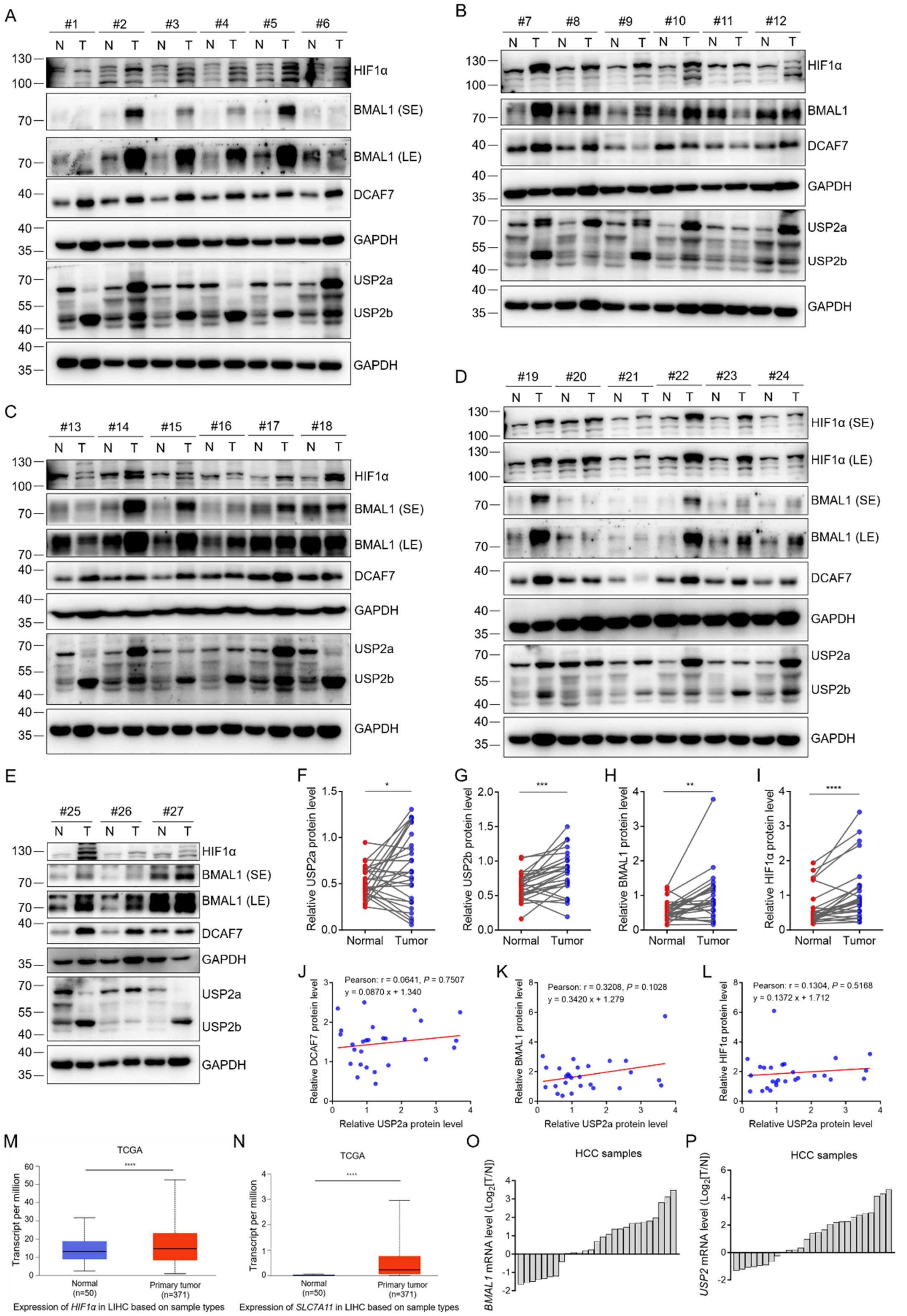

Fig. S8. Clinical relevance of the DCAF7/USP2/BMAL1-HIF1α-SLC7A11 axis in HCC. (**A-E**) The Western blotting analyses of the indicated proteins in HCC tissues and adjacent normal tissues. (**F-I**) The quantification of the indicated proteins in HCC (T, tumor) and matched paratumor (N, normal) tissues. Mean ± SD (n = 27). (**J-L**)

The two-tailed Pearson correlation analyses of the USP2a and DCAF7 (**J**), BMAL1 (**K**), or HIF1α (**L**) protein abundance in HCC tissue samples. (**M** and **N**) The expression of *HIF1α* (**M**) and *SLC7A11* (**N**) mRNA in normal or liver cancer (tumor) samples with different sample types and histological subtypes. Data were obtained from the UALCAN database (https://ualcan.path.uab.edu/index.html). (**O** and **P**) Waterfall plot of the relative *BMAL1* (**O**) and *USP2* (**P**) mRNA level measured by qPCR from 27 HCC and paired paratumor tissues. Each bar represents one case. The P values were calculated using two-tailed, unpaired Student's t-test (**M**, **N**) or paired Student's t-test (**F** to **I**). *$P < 0.05$, **$P < 0.01$, ***$P < 0.001$, ****$P < 0.0001$.

Supplementary Tables

Table S1. Information of antibodies used in this work.

Antibody	Dilution	Supplier	Cat #	RRID
DCAF7	1:1000	ABclonal	A6787	AB_2767370
BMAL1	1:1000	Santa Cruz	sc-365645AC	AB_10841724
HIF1a	1:1000	Proteintech	20960-1-AP	AB_10732601
SLC7A11	1:1000	CST	12691	AB_2687474
USP2	1:1000	Abcepta	AP2131c	AB_2212429
p62	1:2000	Sigma Aldrich	P0068	AB_1841066
Nrf2	1:1000	Proteintech	16396-1-AP	AB_2782956
p53	1:1000	Santa Cruz	sc-126	AB_628082
GFP	1:3000	Proteintech	50430-2-AP	AB_11042881
DDB1	1:1000	Proteintech	11380-1-AP	AB_2088808
Ub	1:1000	Santa Cruz	sc-8017	AB_628423
HA	1:10000	MBL	M180-3	AB_10951811
FLAG	1:10000	MBL	M185-3L	AB_11123930
Myc	1:3000	Proteintech	16386-1-AP	AB_2269755
GAPDH	1:3000	Proteintech	60004-1-Ig	AB_2107436
β-tubulin	1:3000	Proteintech	10068-1-AP	AB_2303998

Table S2. Sequences for primers used for qRT-PCR.

Primer Name	Sequence (5′-3′)
h*BMAL1* q-For	AAGGGAAGCTCACAGTCAGAT
h*BMAL1* q-Rev	GGACATTGCGTTGCATGTTGG
h*DCAF7* q-For	CTGACACAAAAGGCGTCTATCC
h*DCAF7* q-Rev	TGCATGTCGTATCAATGCTTGAG
h*SLC7A11* q-For	TCTCCAAAGGAGGTTACCTGC
h*SLC7A11* q-Rev	AGACTCCCCTCAGTAAAGTGAC
h*GPX4* q-For	GAGGCAAGACCGAAGTAAACTAC
h*GPX4* q-Rev	CCGAACTGGTTACACGGGAA
h*FSP1* q-For	GACTCCTTCCACCACAATGTGG
h*FSP1* q-Rev	CAGCACCATCTGGTTCTTCAGG
h*FTH1* q-For	TCCTACGTTTACCTGTCCATGT
h*FTH1* q-Rev	GTTTGTGCAGTTCCAGTAGTGA
h*SLC3A2* q-For	CTGGTGCCGTGGTCATAATC
h*SLC3A2* q-Rev	GCTCAGGTAATCGAGACGCC
h*TFR1* q-For	ACCATTGTCATATACCCGGTTCA
h*TFR1* q-Rev	CAATAGCCCAAGTAGCCAATCAT
h*ACSL4* q-For	CATCCCTGGAGCAGATACTCT
h*ACSL4* q-Rev	TCACTTAGGATTTCCCTGGTCC
h*HIF1a* q-For	GAACGTCGAAAAGAAAAGTCTCG
h*HIF1a* q-Rev	CCTTATCAAGATGCGAACTCACA
h*FABP3* q-For	CATGACCAAGCCTACCACAAT
h*FABP3* q-Rev	CCCCAACTTAAAGCTGATCTCTG
h*CP* q-For	GGGCCATCTACCCTGATAACA
h*CP* q-Rev	TTAAAGGTCCGATGAGTCCTGA
h*GAPDH* q-For	CCATCAATGACCCCTTCATTGACC
h*GAPDH* q-Rev	GAAGGCCATGCCAGTGAGCTTCC

Table S3. siRNA sequences used in this work.

siRNA	Sequence (5′-3′)
si*DCAF7*#1 sense	CUUGGUUUAGAUGAGGAGAGUTT
si*DCAF7*#1 antisense	ACUCUCCUCAUCUAAACCAACTT
si*DCAF7*#2 sense	CCUGAAGGAGAGAUCAACAATTT
si*DCAF7*#2 antisense	AUUGUUGAUCUCUCCUUCAGCTT
si*DDB1*#2 sense	CGGCAUUAUUGGCAUCAUUTT
si*DDB1*#2 antisense	AAUGAUGCCAAUAAUGCCGTT

Table S4. Sequences for primers used for plasmid construction.

Primer Name	Sequence (5′-3′)
pcDNA3.1-Myc-USP2a-For	CTTGGTACCGAGCTCGGATCC**ATGTCCCAGCTCTCCTCCAC**
pcDNA3.1-Myc-USP2a-Rev	CTGTGCTGGATATCTGCAGAATTC**CATTCGGGAGGGCGGG**
pcDNA3.1-Myc-USP2b-For	CTTGGTACCGAGCTCGGATCC**ATGCGCACCTCGTACACC**
pcDNA3.1-Myc-USP2b-Rev	CTGTGCTGGATATCTGCAGAATTC**CATTCGGGAGGGCGG**
pCDH-HA-DCAF7 For	GTCCCAGACTACGCAGCTAGC**ATGTCCCTGCACGGCAAA**
pCDH-HA-DCAF7 Rev	ATCCGATTTAAATTCGAATTC**CTACACTCTGAGTATCTCCAGGC**
pcDNA3.1-FLAG-DCAF7 For	CTTGGTACCGAGCTCGGATCC*ATGGATTACAAGGACGACGATGACAAG***ATGTCCCTGCACGGCAAA**
pcDNA3.1-FLAG-DCAF7 Rev	CTGTGCTGGATATCTGCAGAATTC**CTACACTCTGAGTATCTCCAGGC**
pcDNA3.1-HA-SLC7A11 For	GTCCCAGACTACGCAGCTAGC**ATGGTCAGAAAGCCTGTTGTG**
pcDNA3.1-HA-SLC7A11 Rev	ATCCGATTTAAATTCGAATTC**GAAGTTGTACCAGAAGAAGATAAGTTATGA**
pGEX-GST-His$_6$-DCAF7 For	TTGTATTTCCAGGGCCATATG**ATGTCCCTGCACGGCAAAC**
pGEX-GST-His$_6$-DCAF7 Rev	AGTCTGCAGGGTACCAAGCTT**CTAGACATCTAGAATCACCA**
pMAL-MBP-His$_6$-USP2a For	CAGACTAATGCTGCTGCGGAATTC**ATGTCCCAGCTCTCCTCCAC**
pMAL-MBP-His$_6$-USP2a Rev	CATATGCCGACCCTGTTTG**CTACATTCGGGAGGGCGG**
pMAL-MBP-His$_6$-USP2b For	CAGACTAATGCTGCTGCGGAATTC**ATGTCCCAGCTCTCCTCCAC**
pMAL-MBP-His$_6$-USP2b Rev	CATATGCCGACCCTGTTTG**CTACATTCGGGAGGGCGG**

Notes: Homologous arm (+ *FLAG tag sequence*) + **Primers**

Table S5. shRNA sequences for the construction of shRNA plasmids.

shRNA	Sequence (5′-3′)
sh*DCAF7*#1 For	CCGGGCAAACGGAAGGAGATCTACACTCGAGTGTAGATCTCCTTCCGTTTGCTTTTTG
sh*DCAF7*#1 Rev	AATTCAAAAAGCAAACGGAAGGAGATCTACACTCGAGTGTAGATCTCCTTCCGTTTGC
sh*DCAF7*#2 For	CCGGGGAGTACAACAACAAGGTTCACTCGAGTGAACCTTGTTGTTGTACTCCTTTTTG
sh*DCAF7*#2 Rev	AATTCAAAAAGGAGTACAACAACAAGGTTCACTCGAGTGAACCTTGTTGTTGTACTCC
sh*DCAF7*#3 For	CCGGGGTTTAGATGAGGAGAGTTCA CTCGAGTGAACTCTCCTCATCTAAACC TTTTTG
sh*DCAF7*#3 Rev	AATTCAAAAAGGTTTAGATGAGGAGAGTTCACTCGAGTGAACTCTCCTCATCTAAACC
sh*p62*#1 For	CCGGGGCCTACCTTCTGGGCAAGGACTCGAGTCCTTGCCCAGAAGGTAGGCCTTTTTG
sh*p62*#1 Rev	AATTCAAAAAGGCCTACCTTCTGGGCAAGGACTCGAGTCCTTGCCCAGAAGGTAGGCC
