# Supplementary material for: DCAF7 recruits USP2 to facilitate hepatocellular carcinoma progression by suppressing clockophagy-induced ferroptosis
Source: Cell Death Dis. 2025 Aug 28;16(1):654. doi: 10.1038/s41419-025-07977-3 (PMC12394690; doi:10.1038/s41419-025-07977-3)

Figure 1D

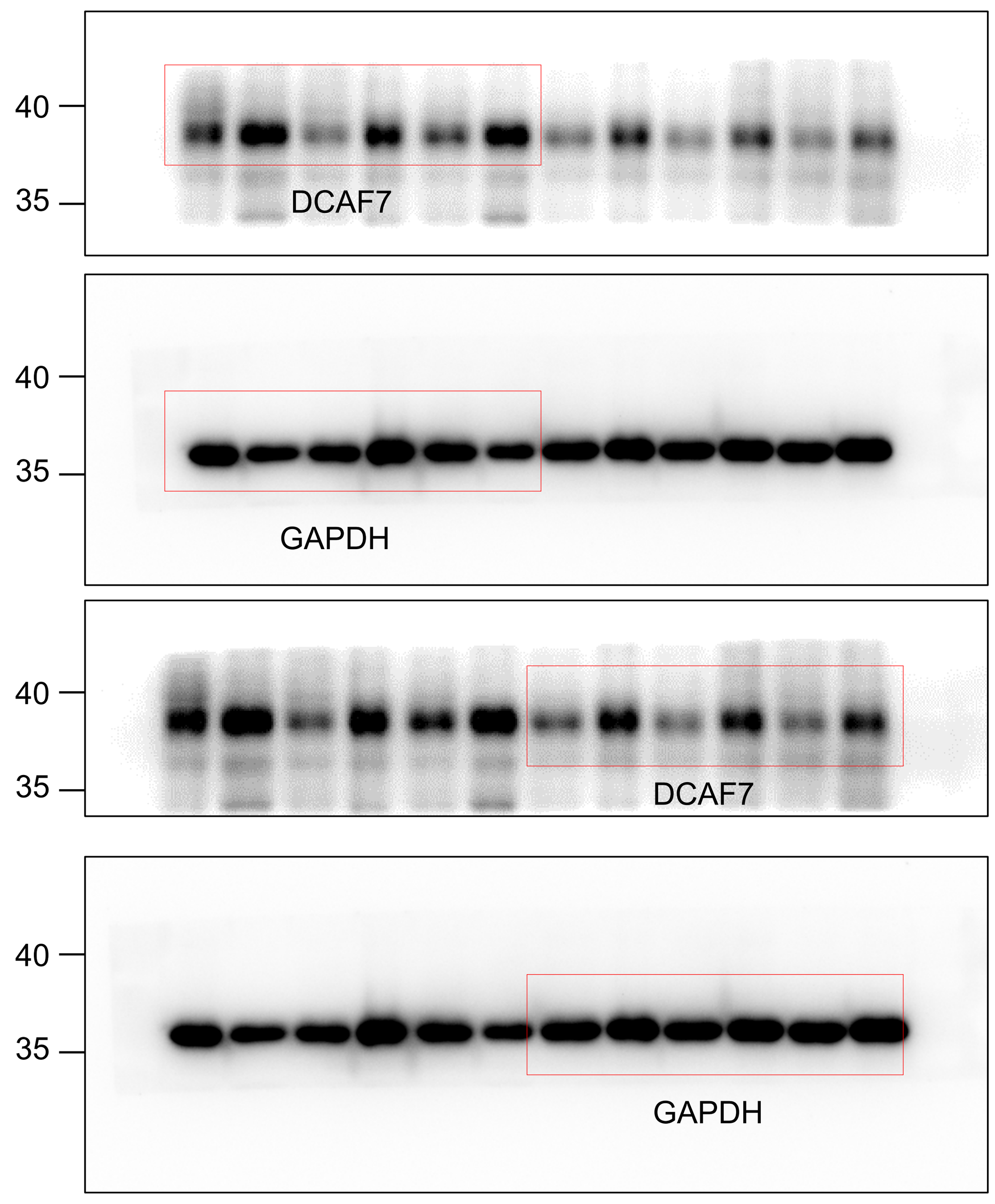

Figure 1E

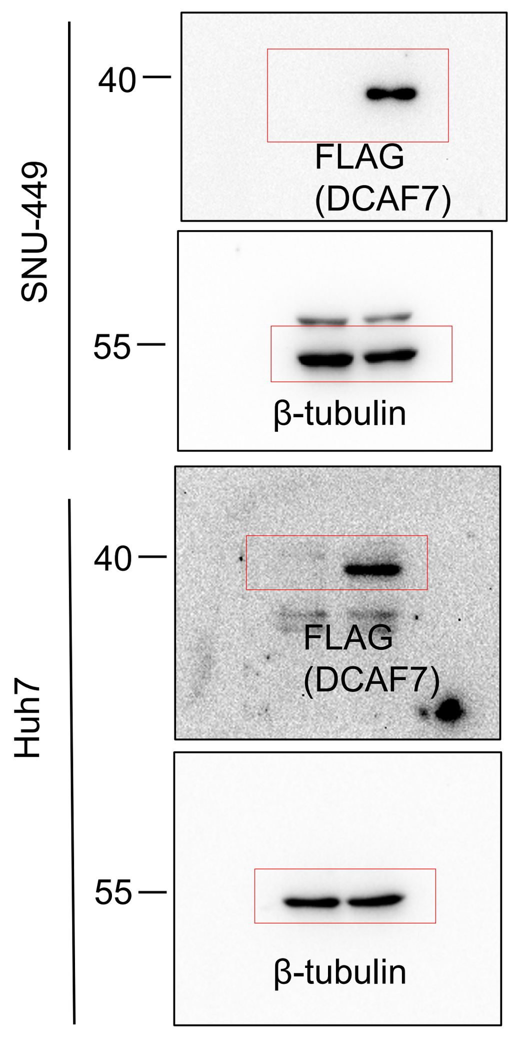

Figure 1I

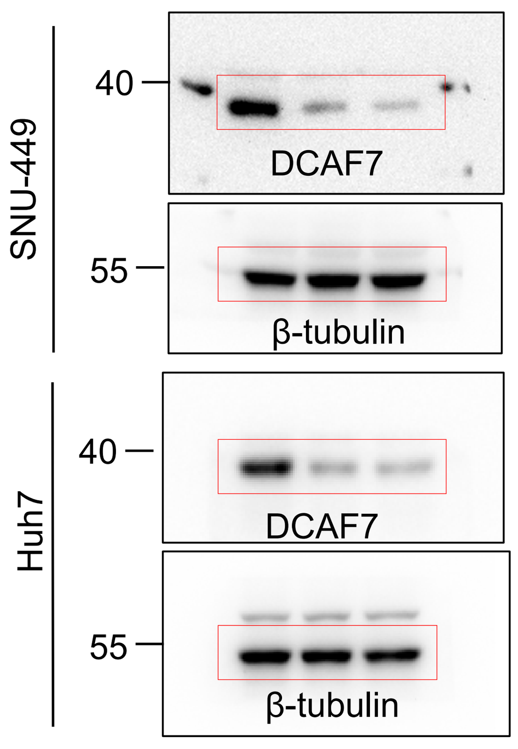

Figure 2I

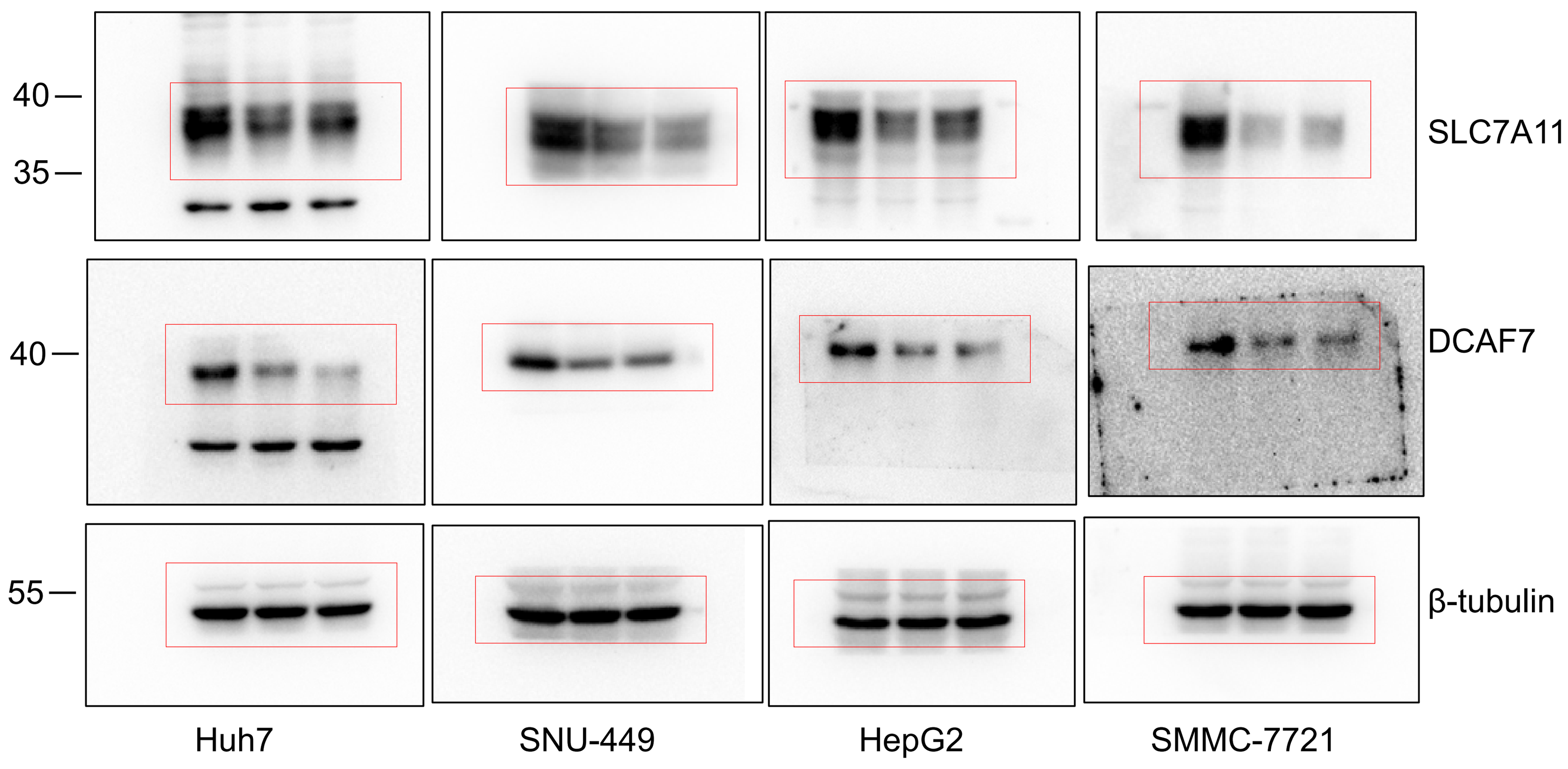

Figure 2M

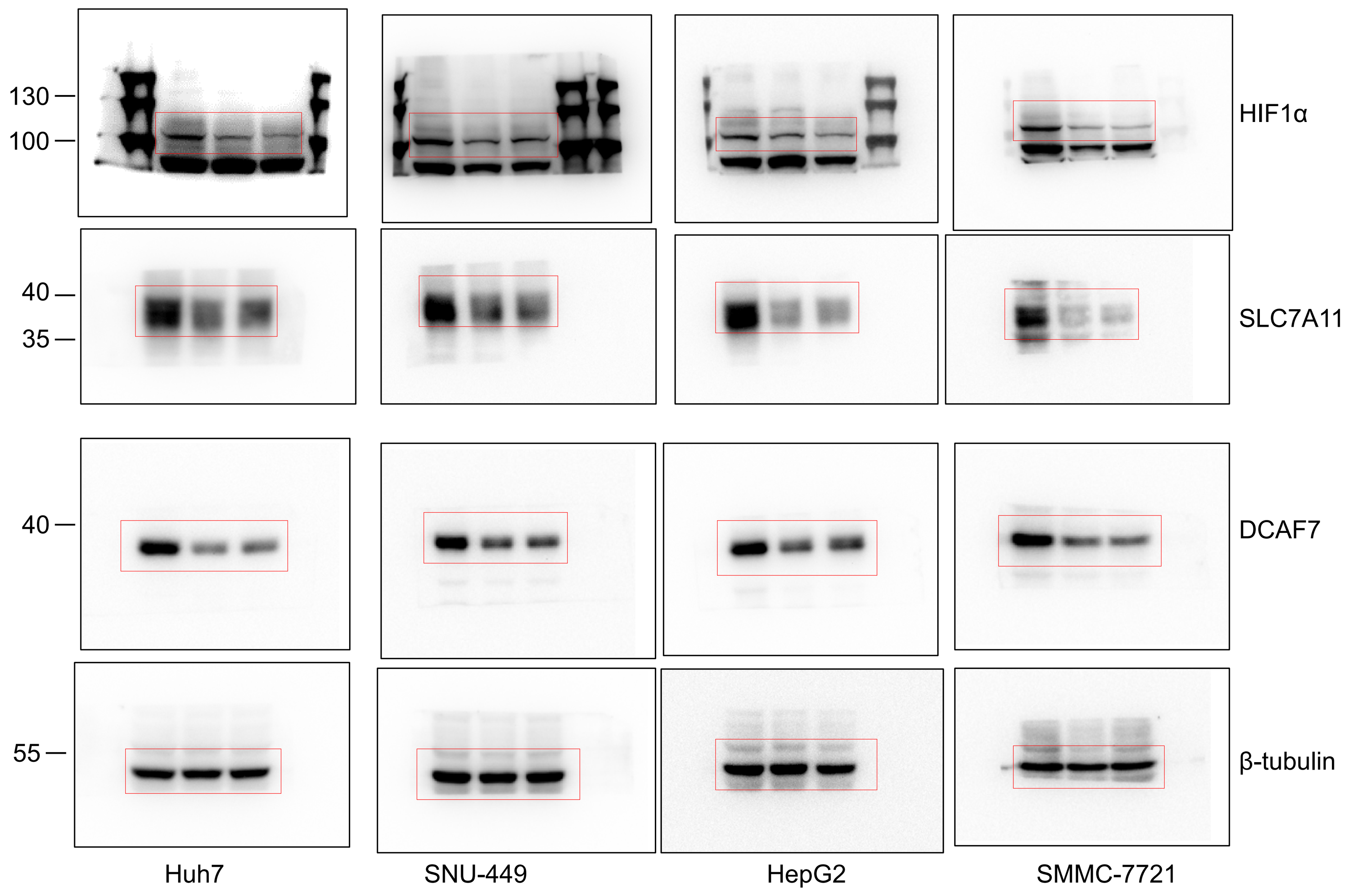

Figure 2P

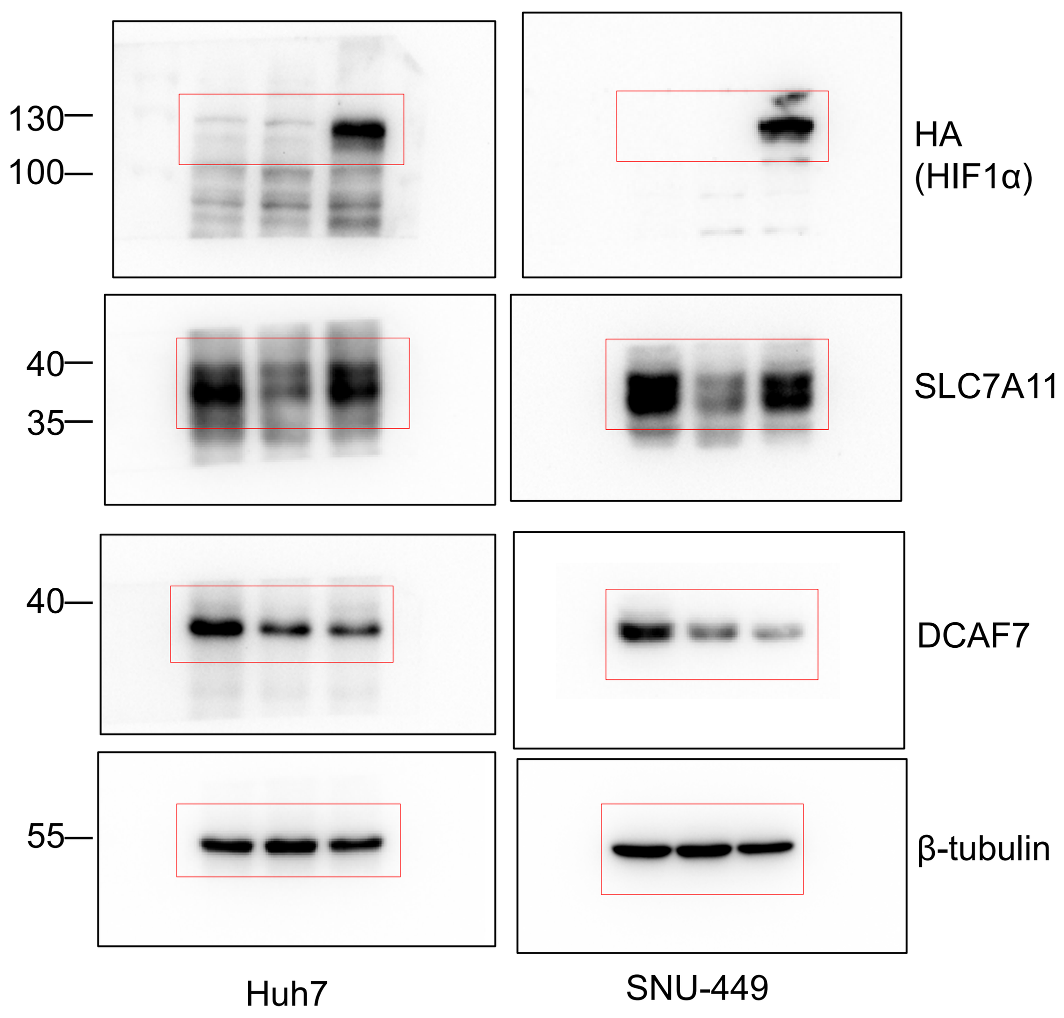

Figure 3C

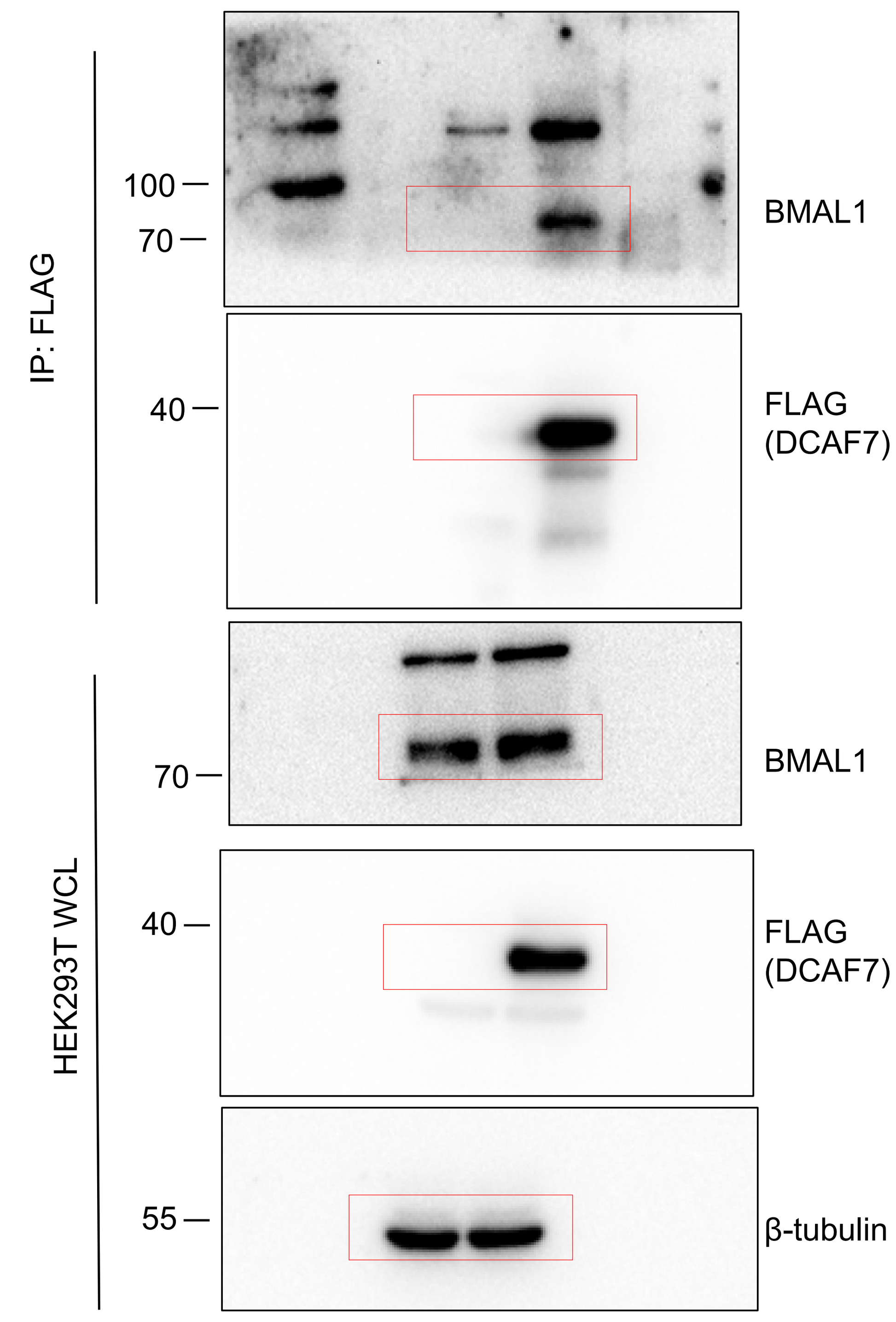

Figure 3D

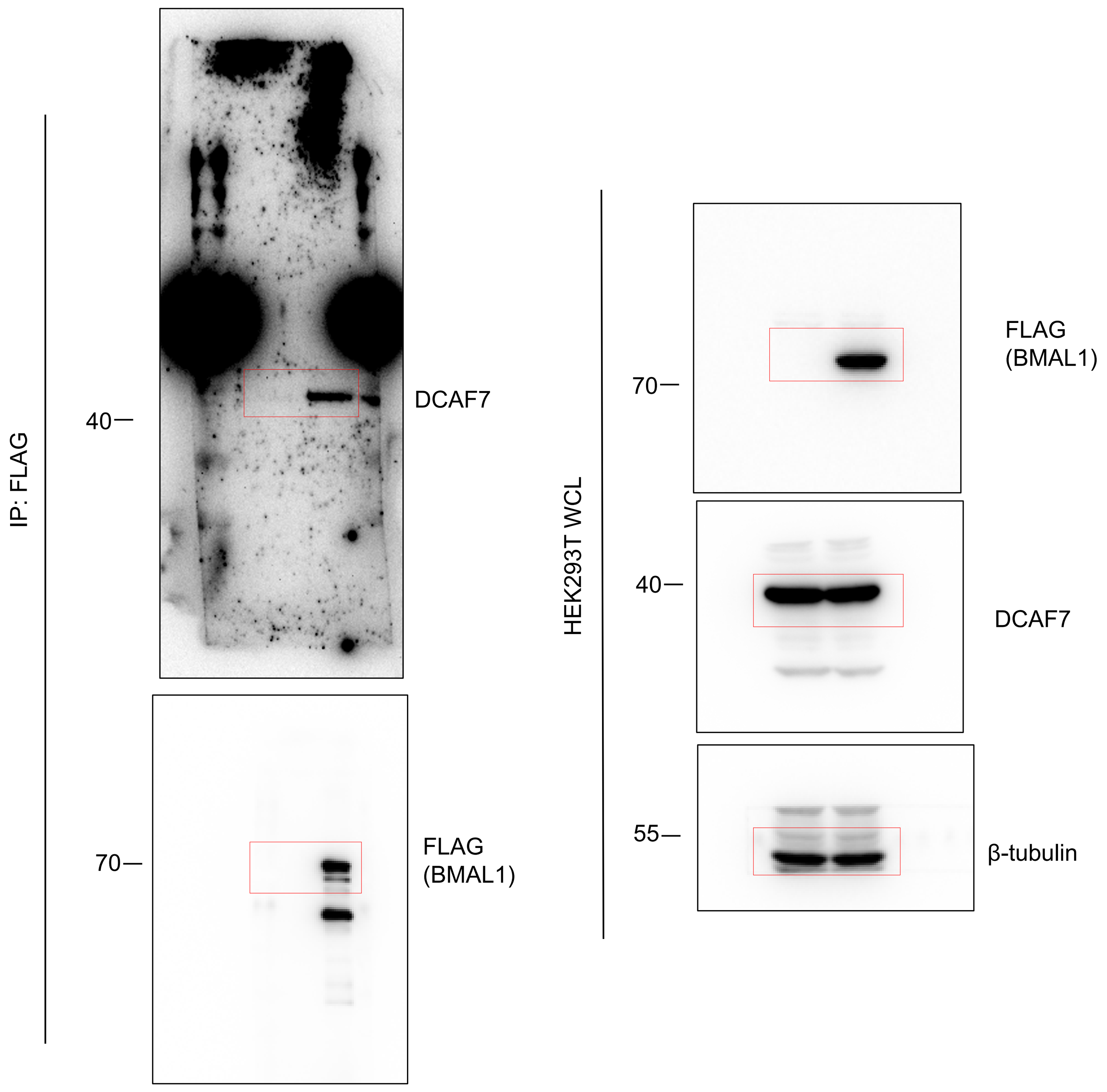

Figure 3E

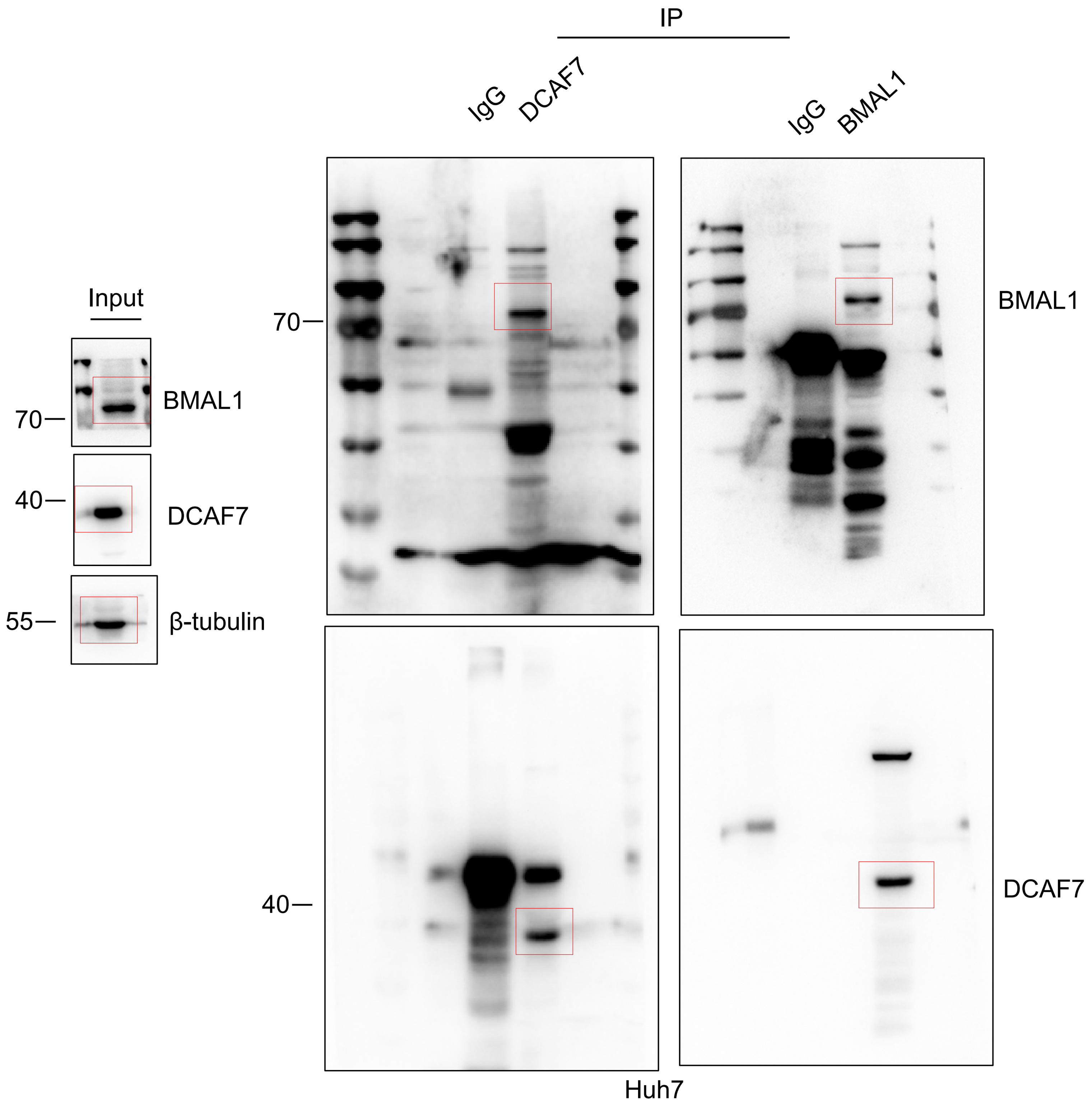

Figure 3F

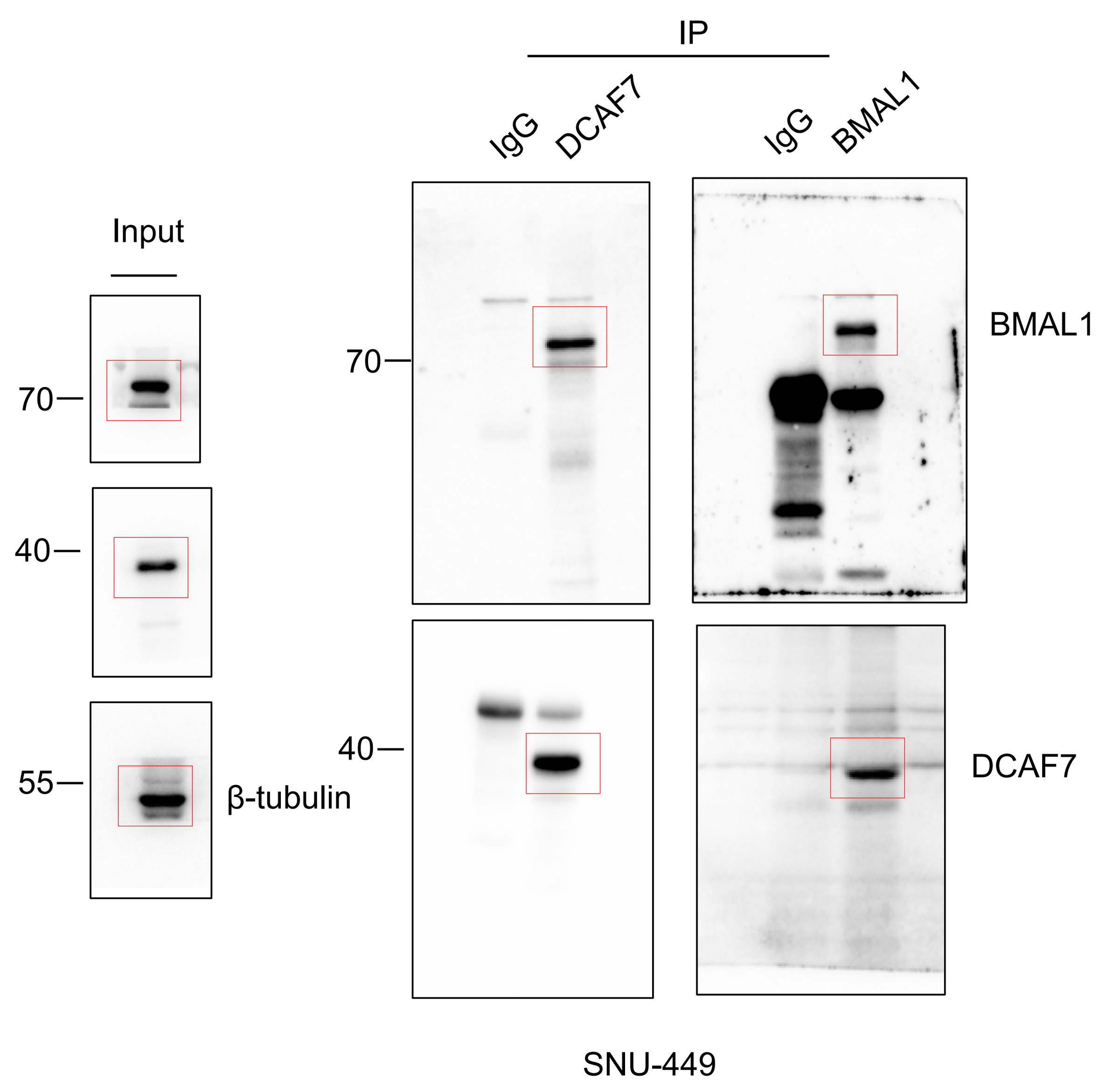

Figure 3I

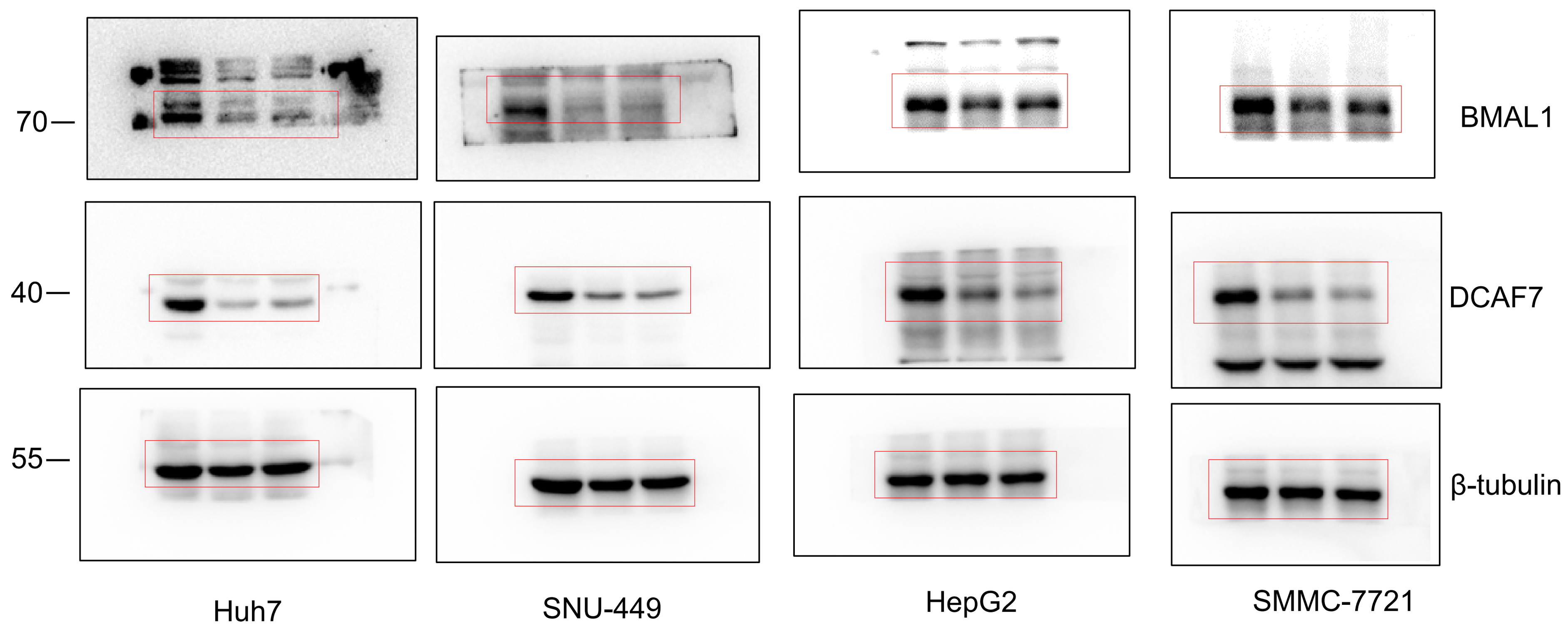

Figure 3M

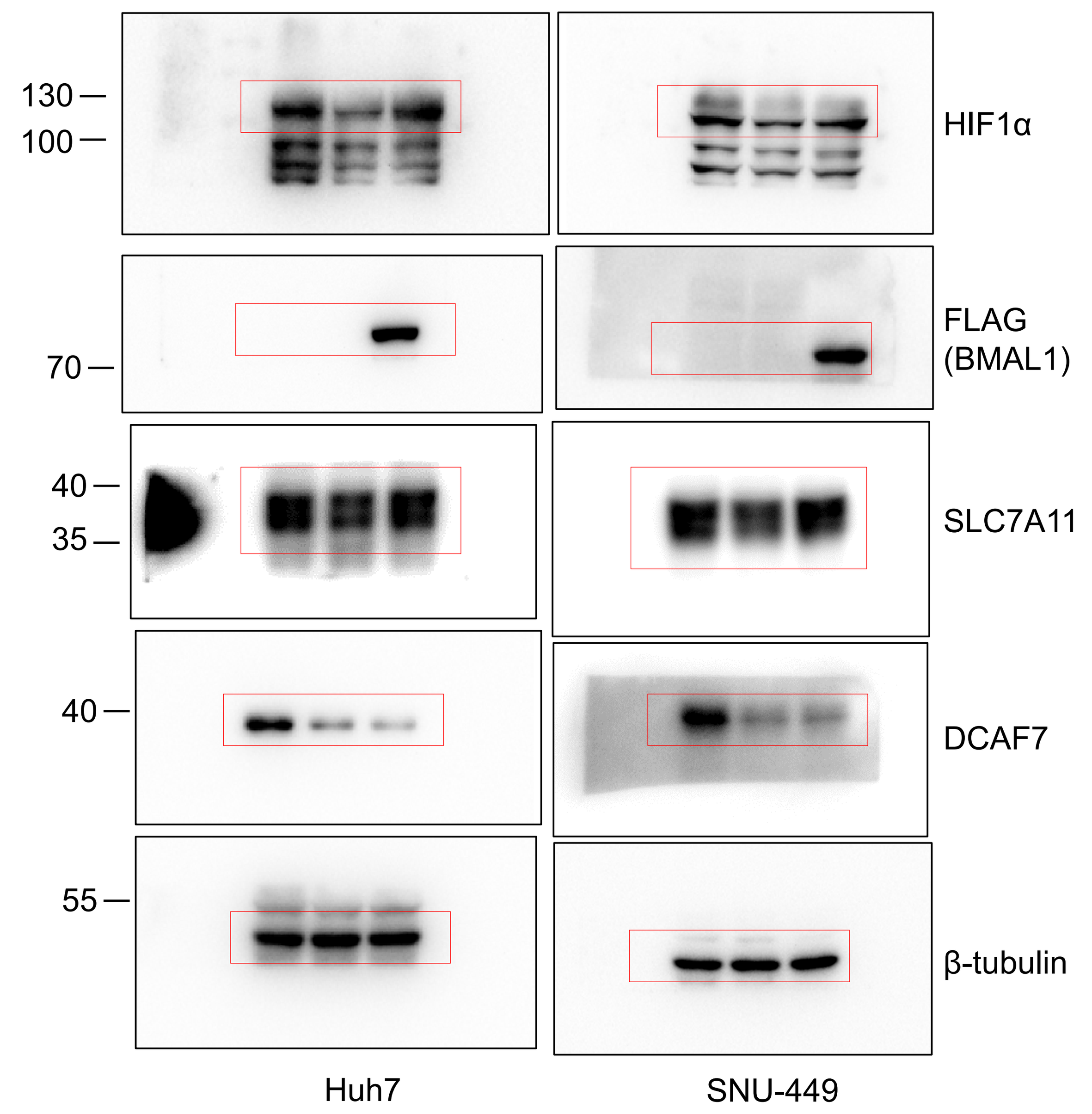

Figure 4A

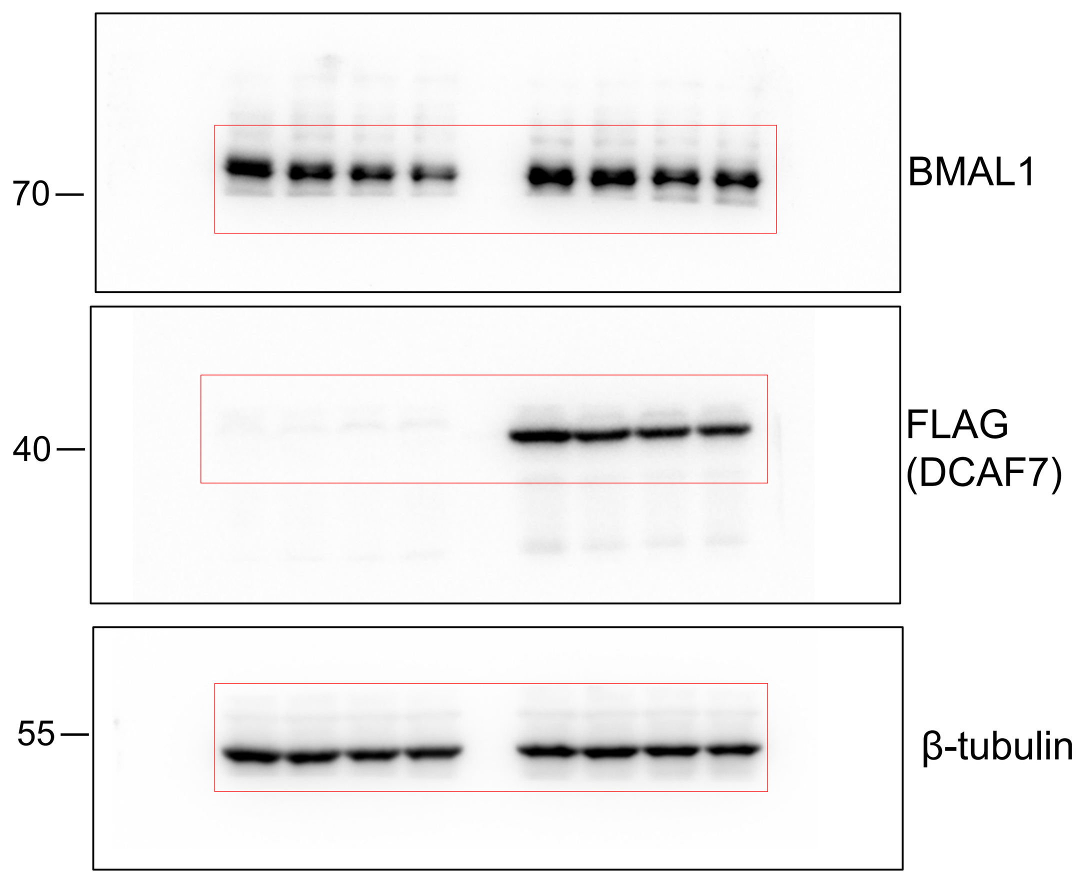

Figure 4B

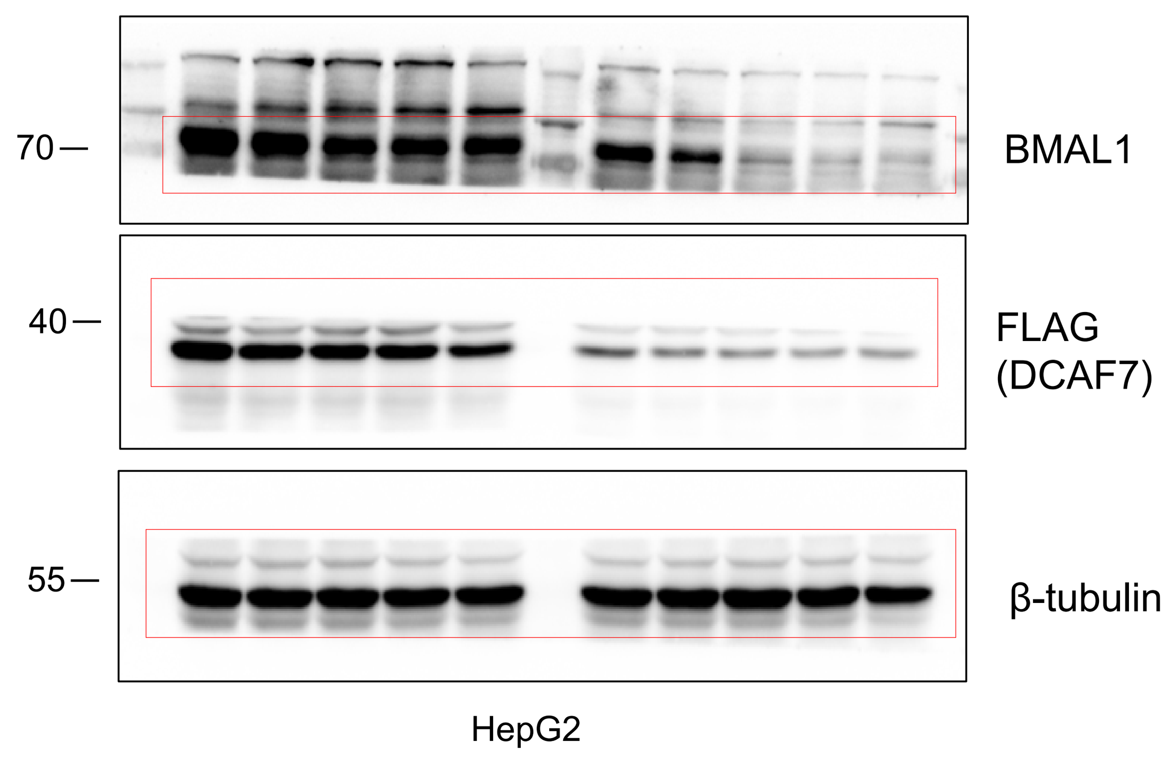

Figure 4C

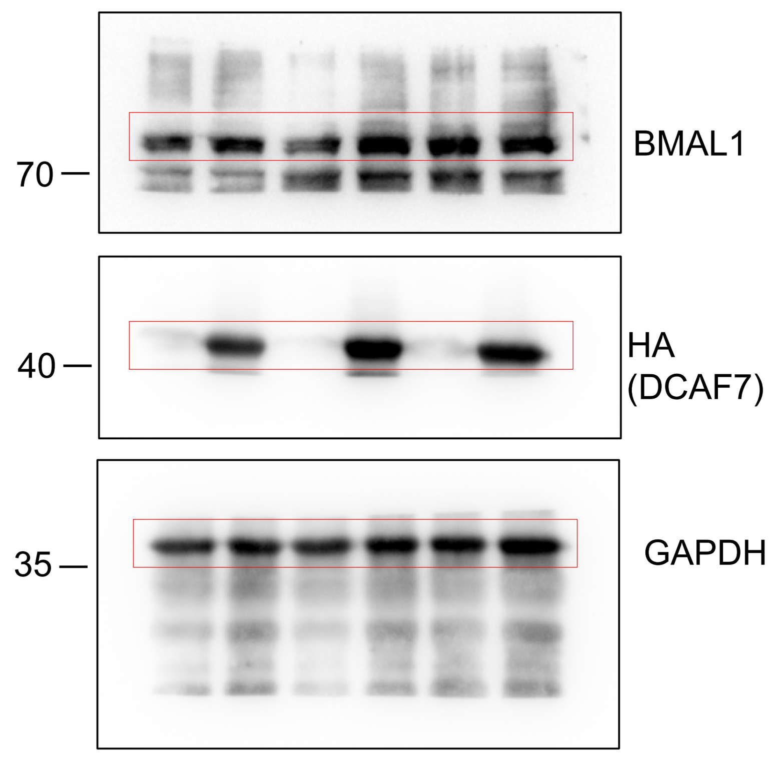

Figure 4D

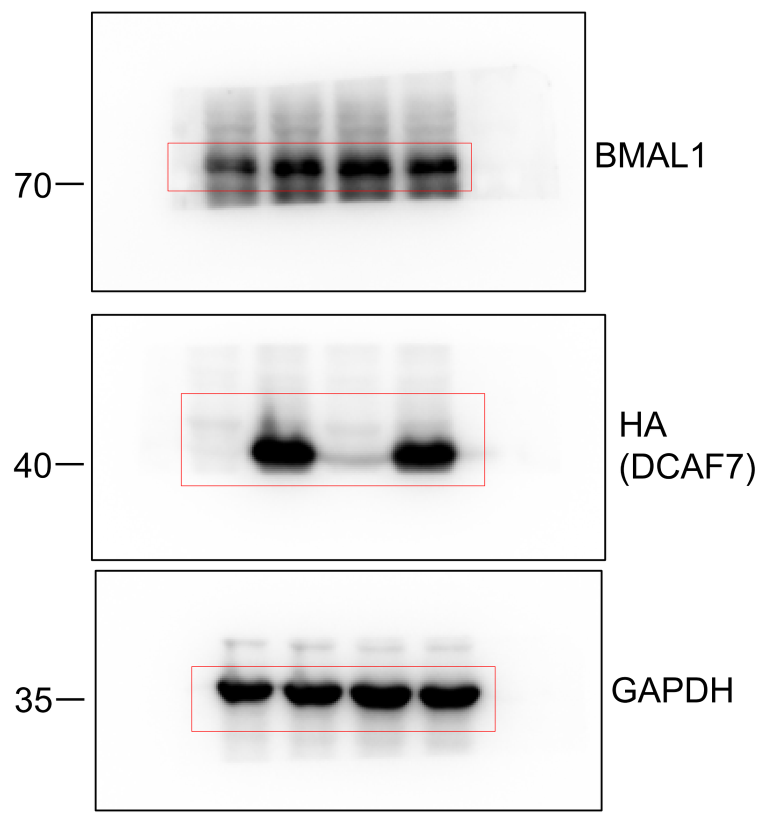

Figure 4E

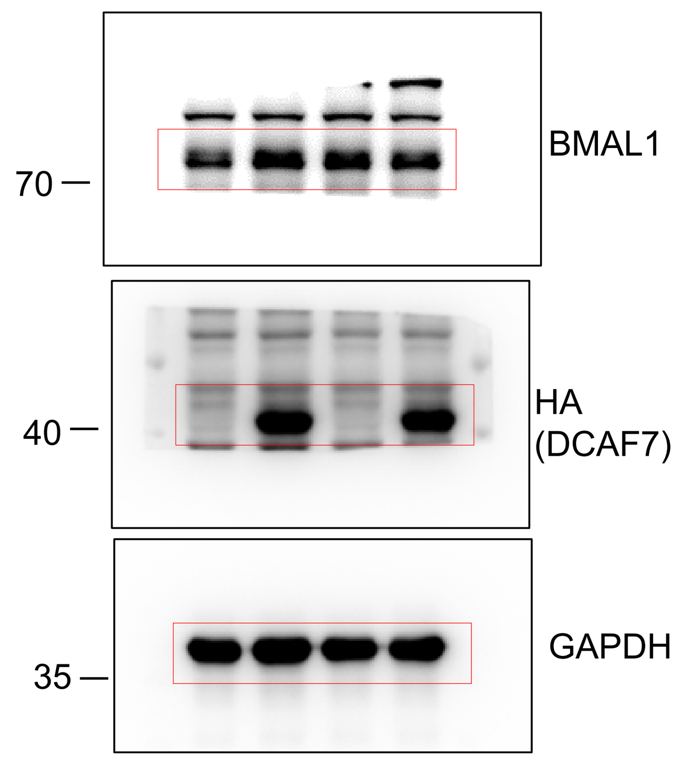

Figure 4F

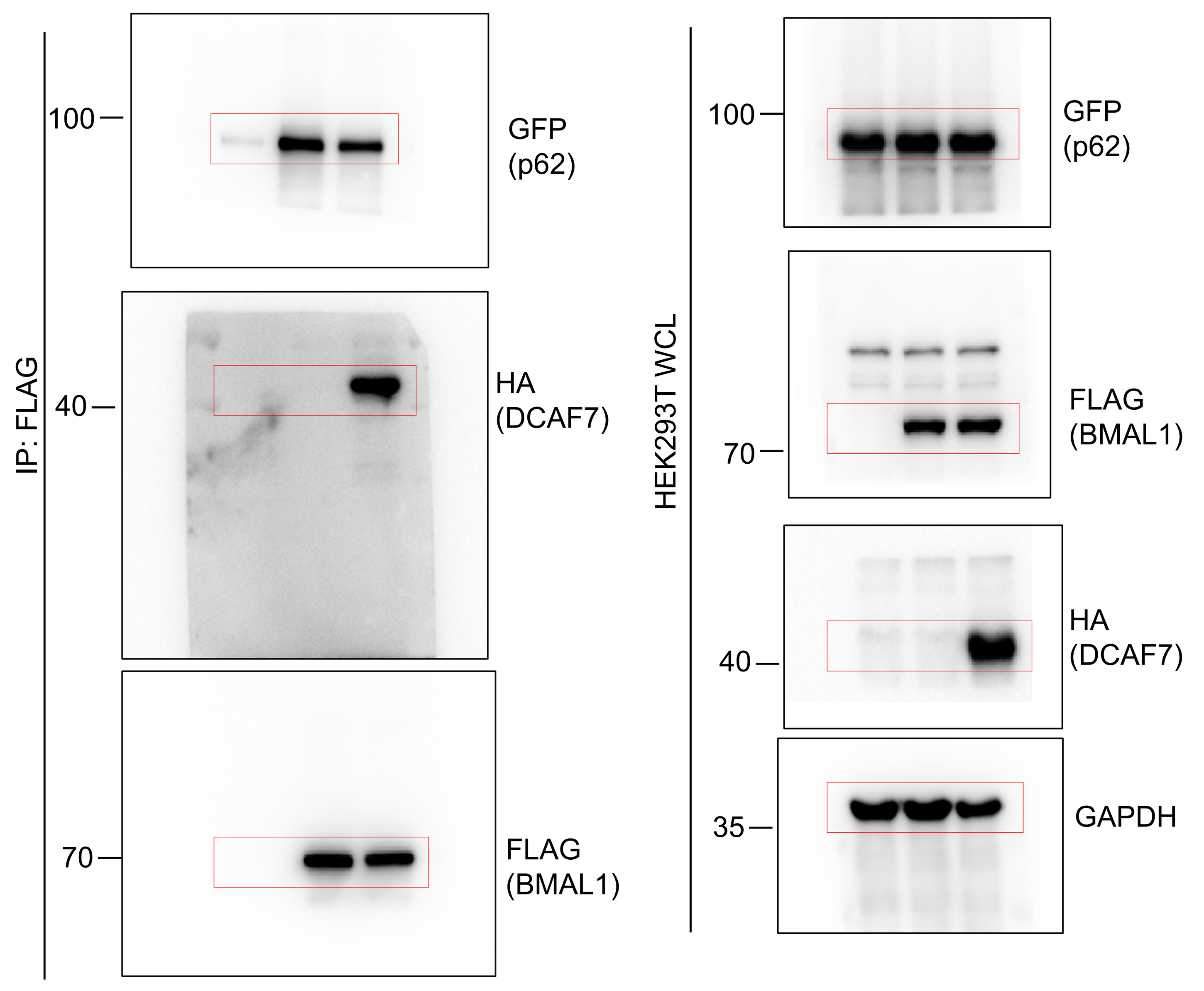

Figure 4G

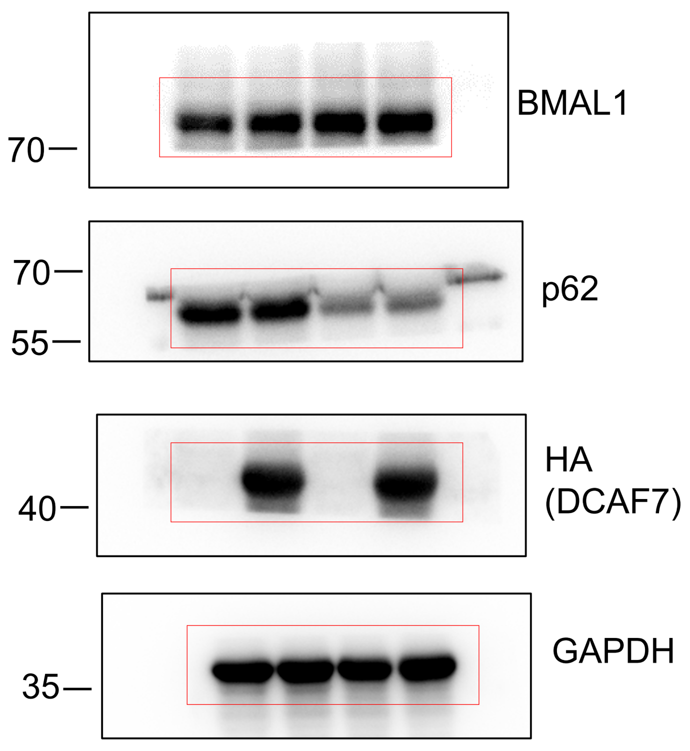

Figure 4H

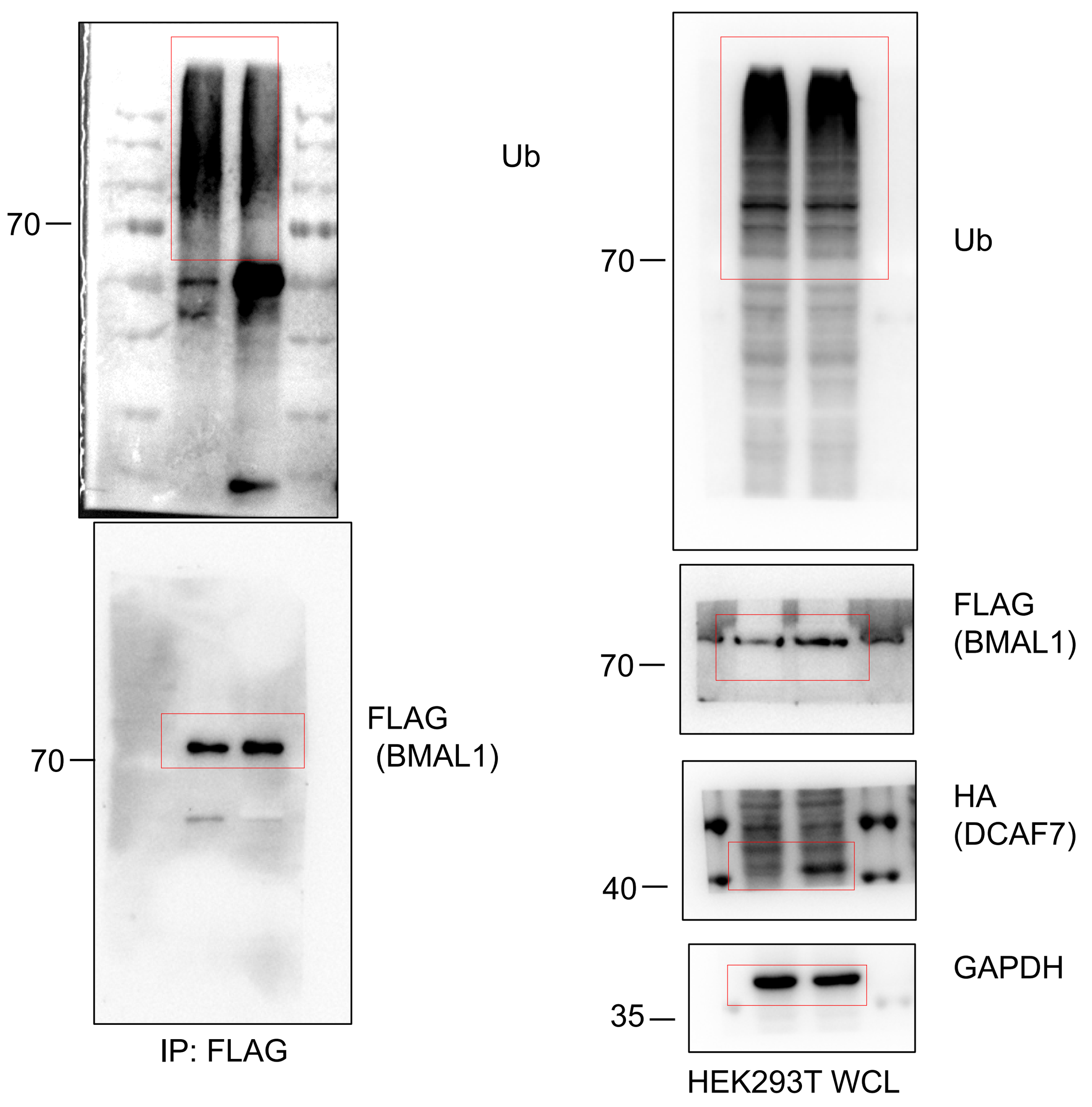

Figure 4I

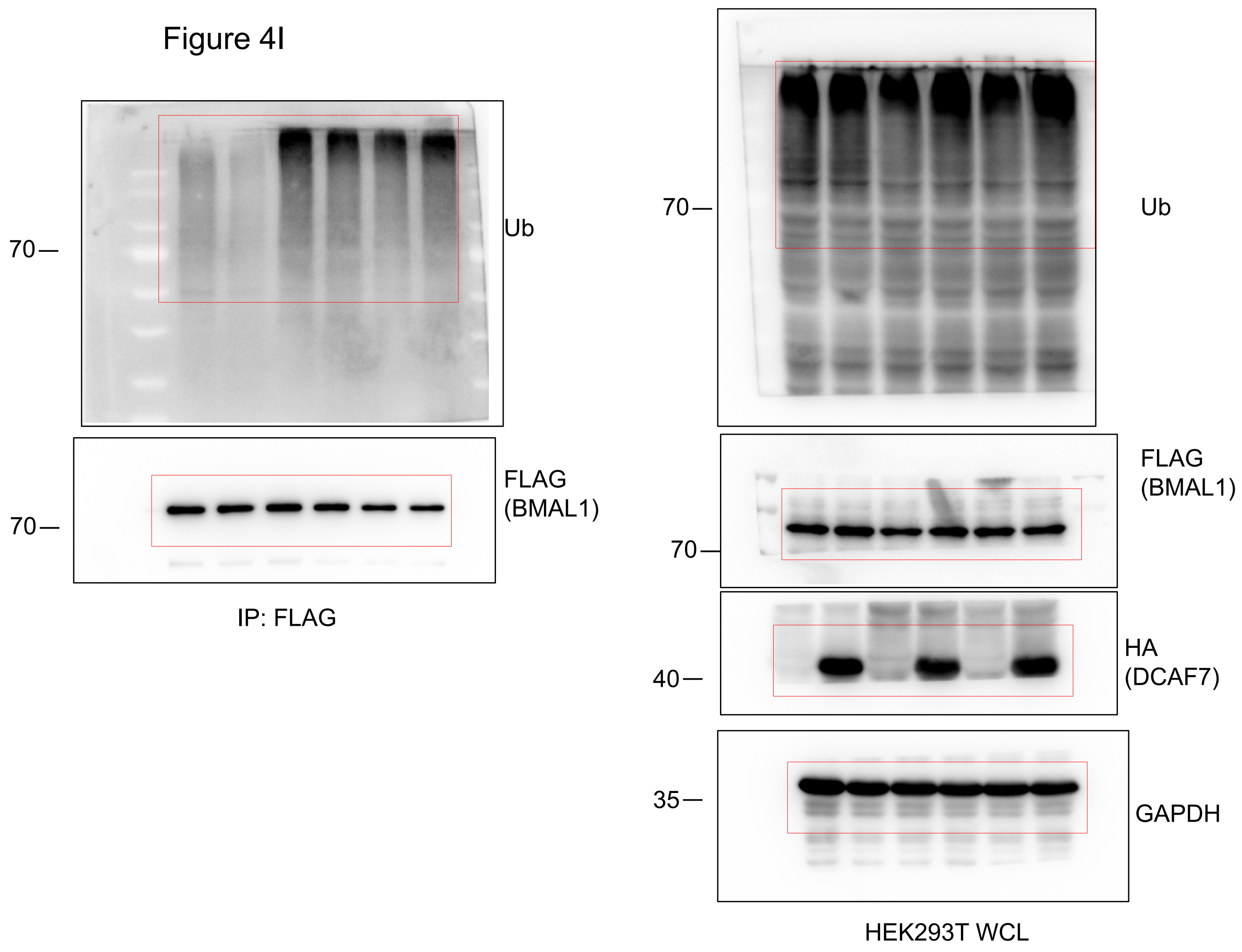

Figure 5A

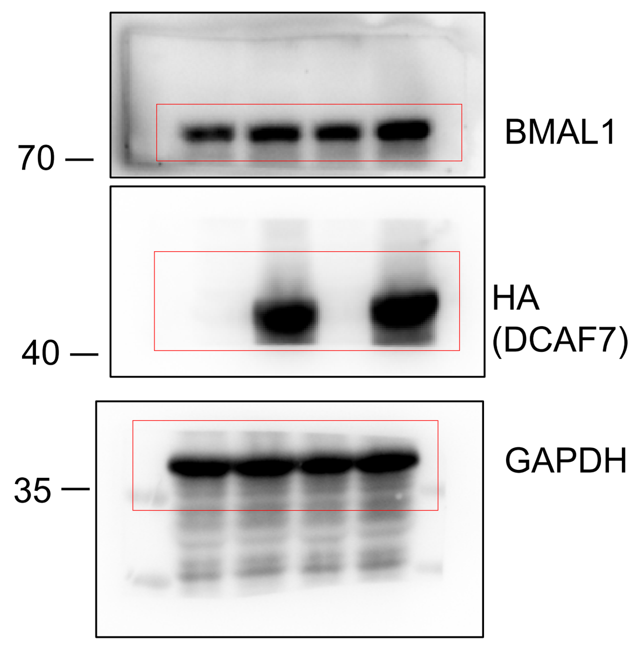

Figure 5B

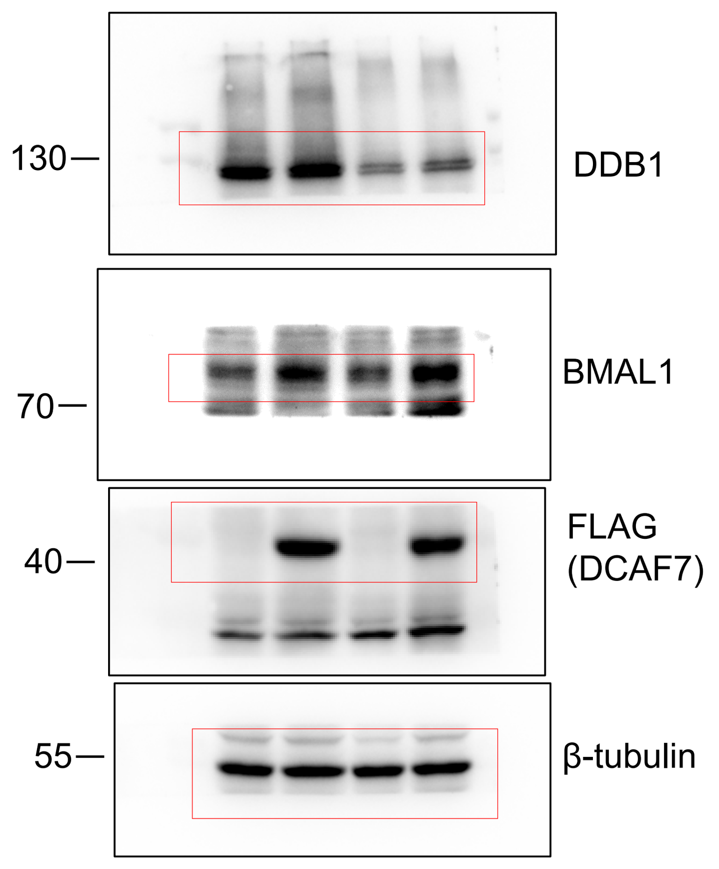

Figure 5C

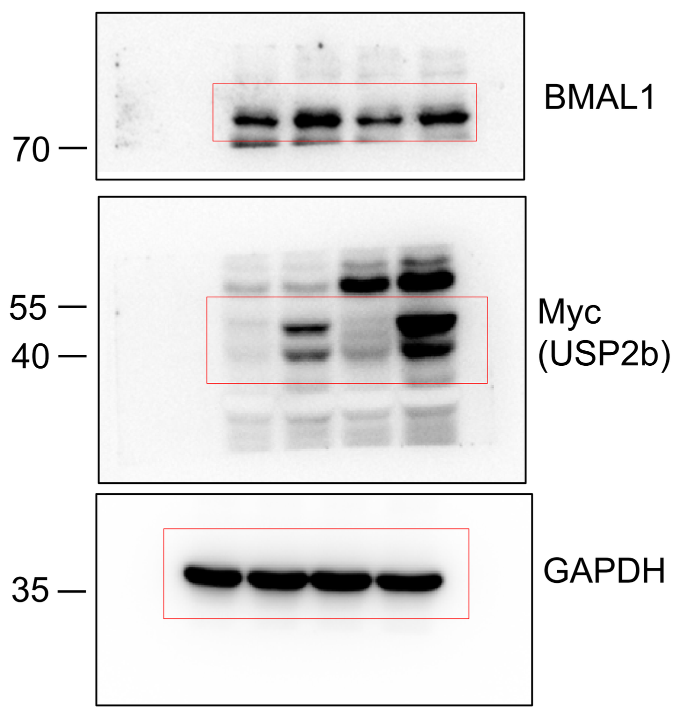

Figure 5D

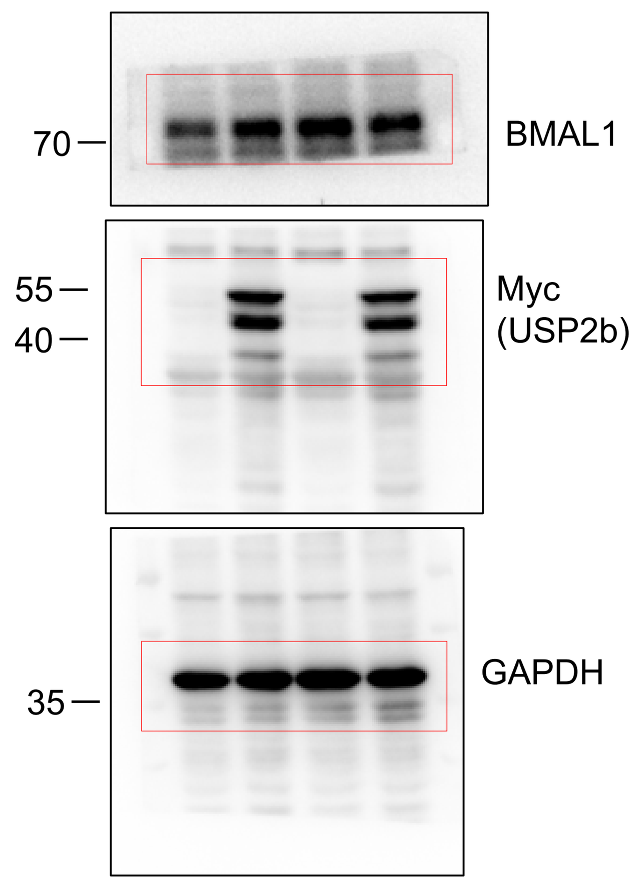

Figure 5E

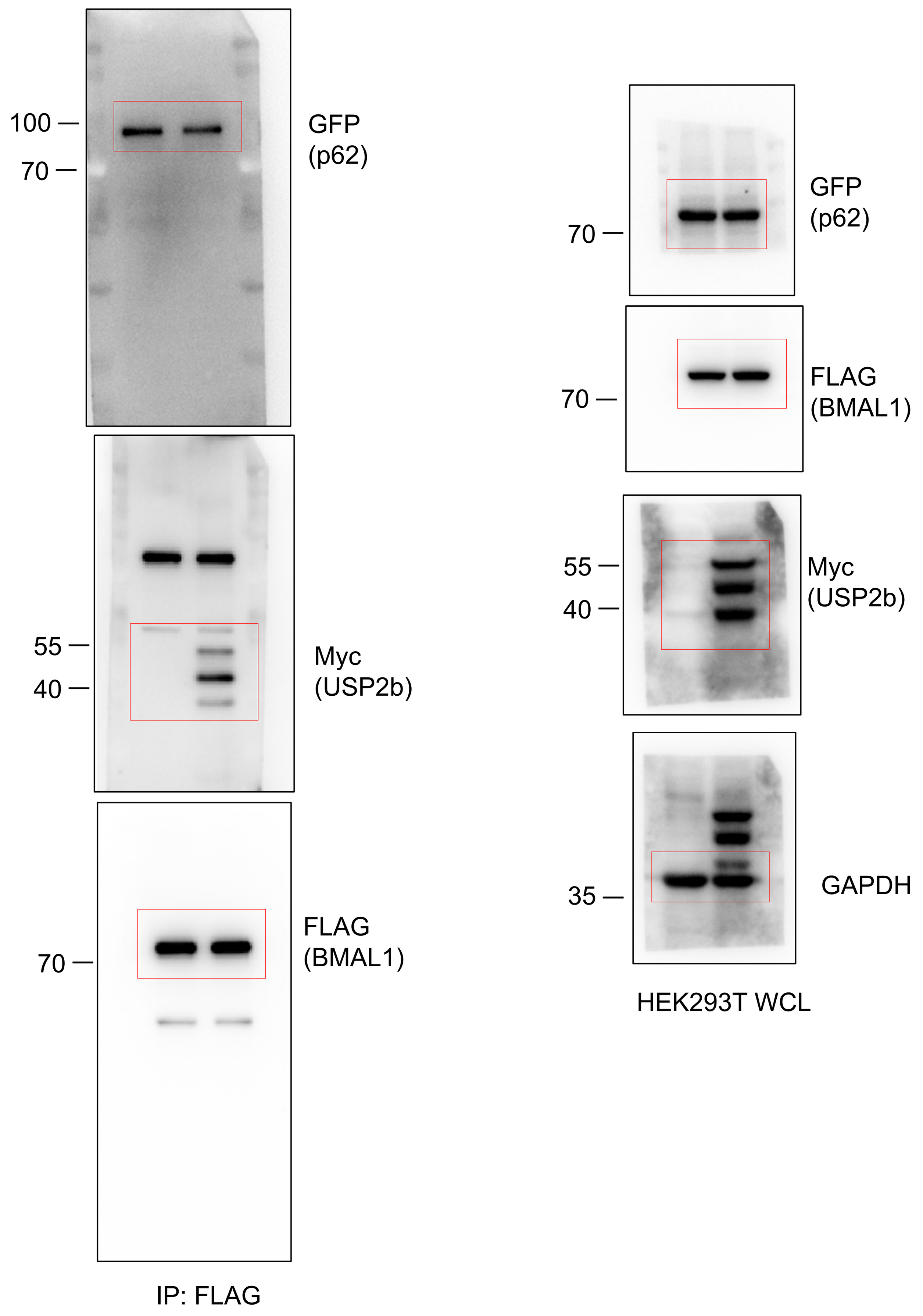

Figure 5F

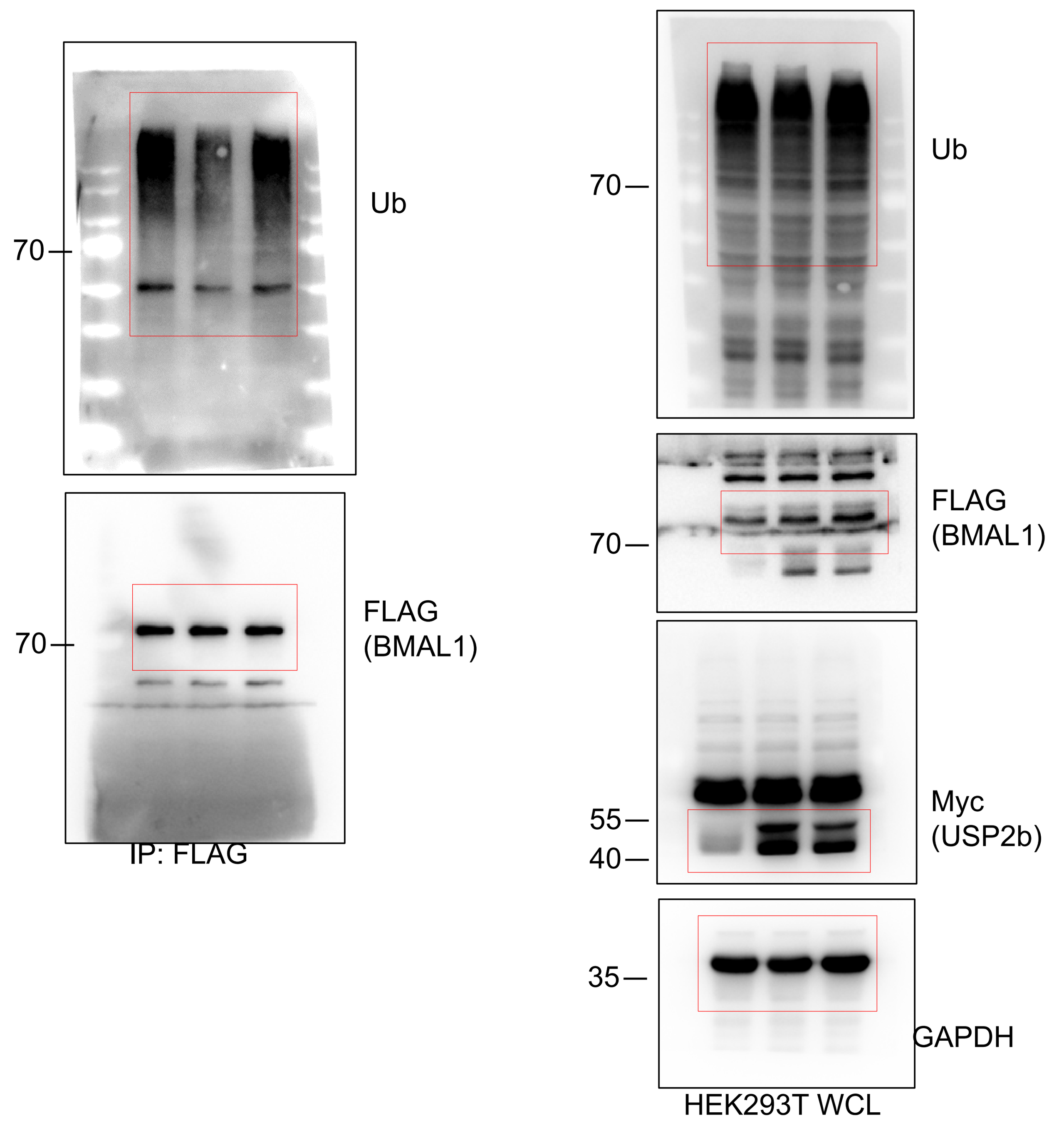

Figure 5G

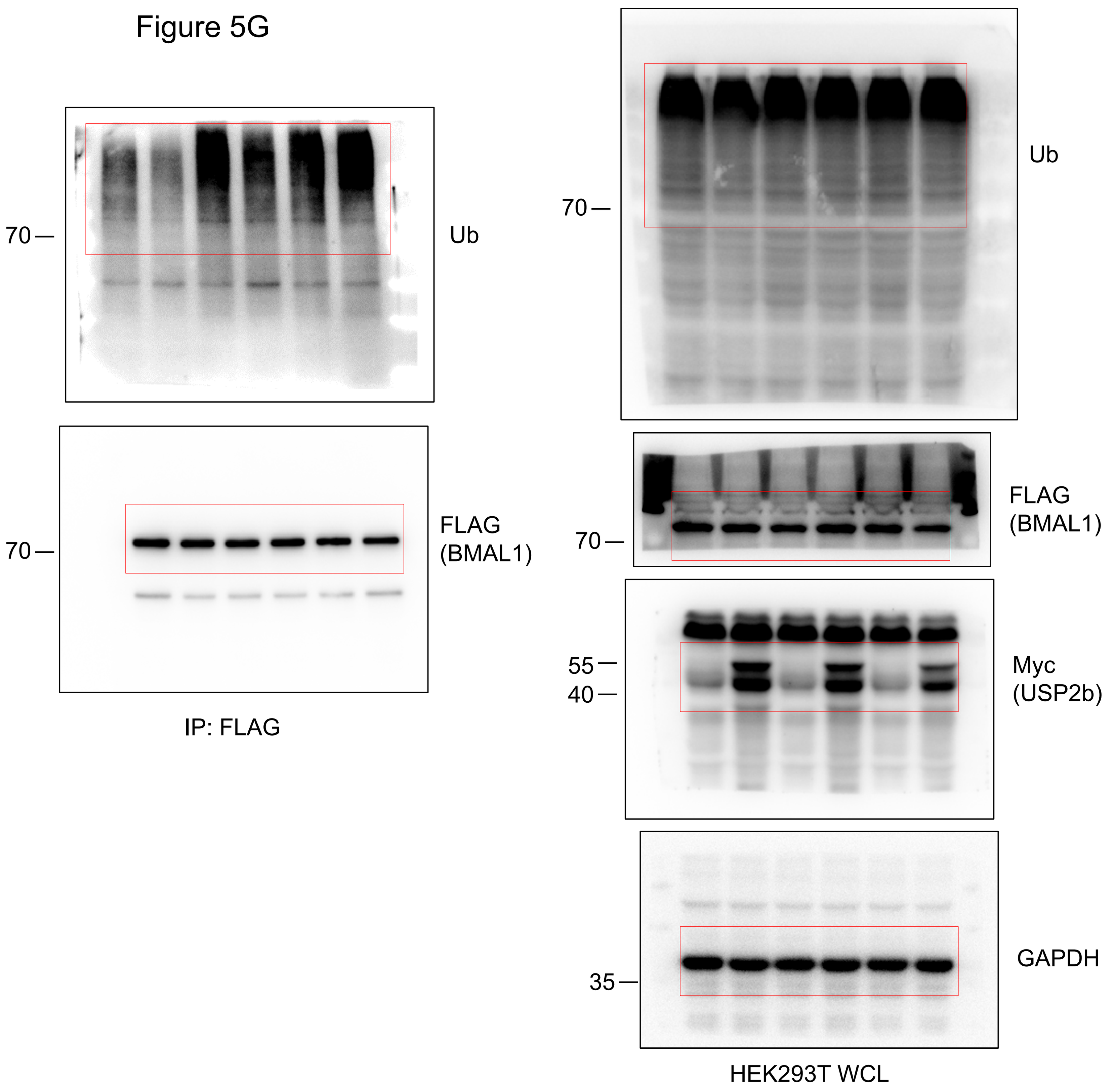

Figure 5I

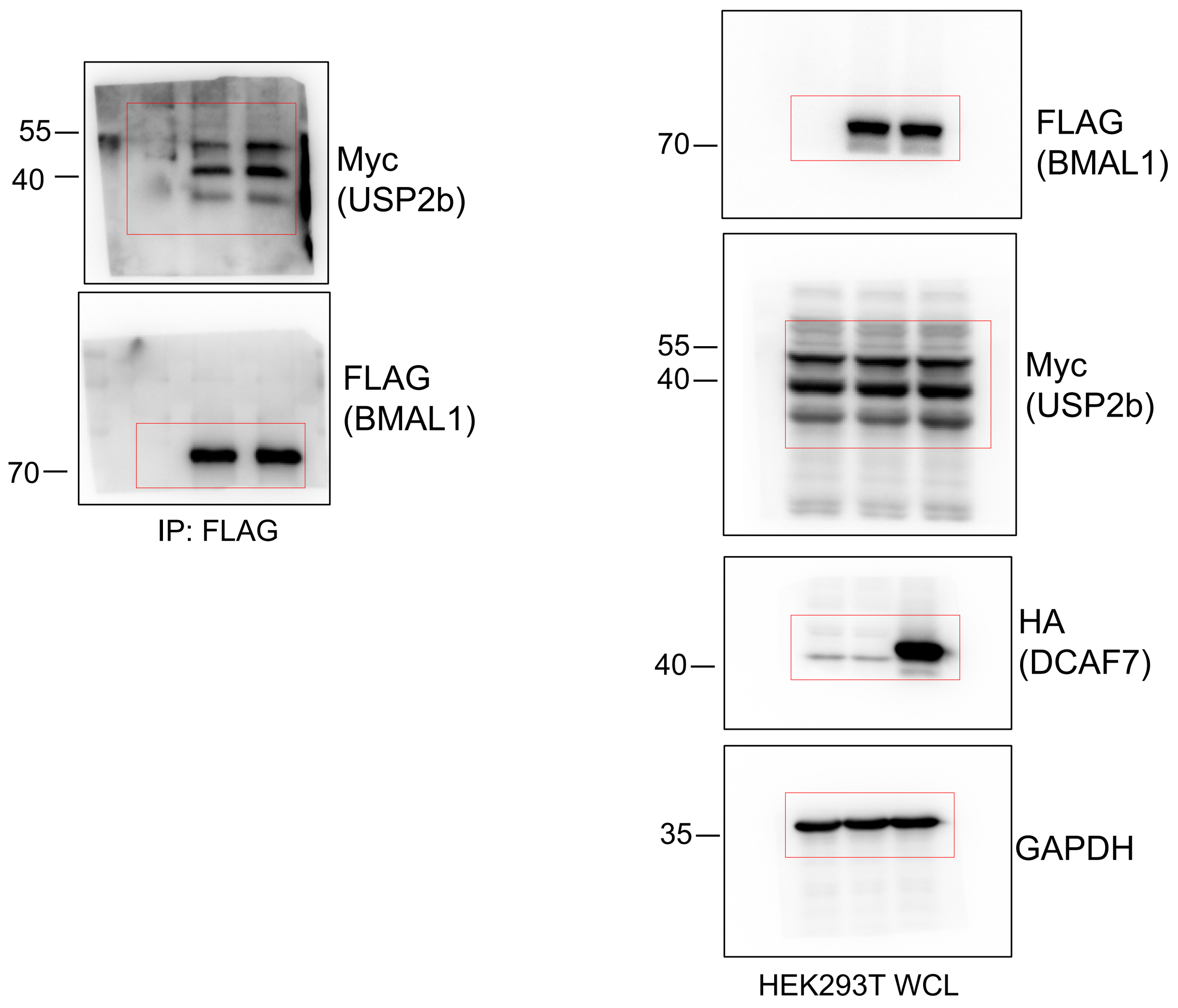

Figure 5J

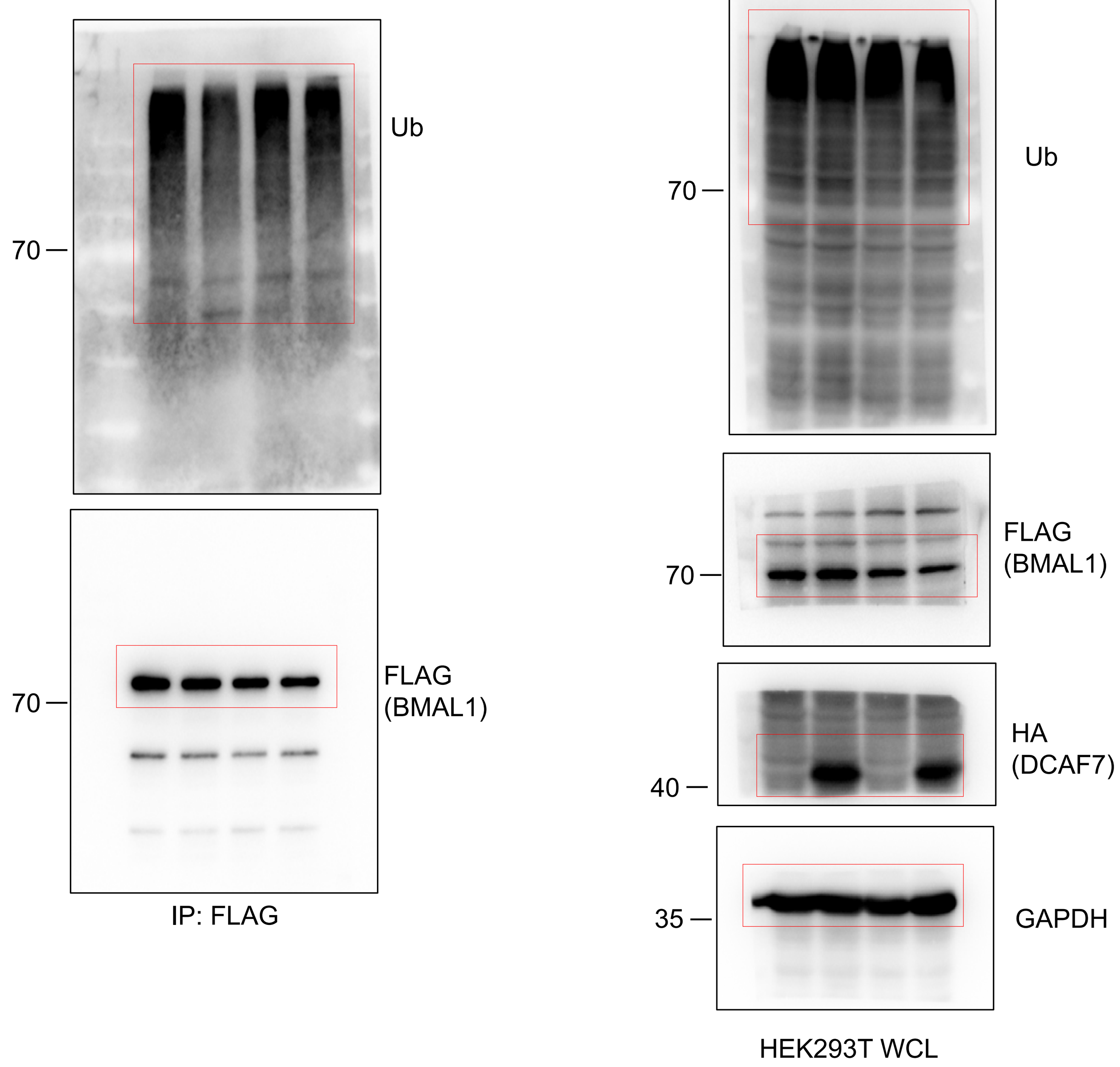

Figure 5K

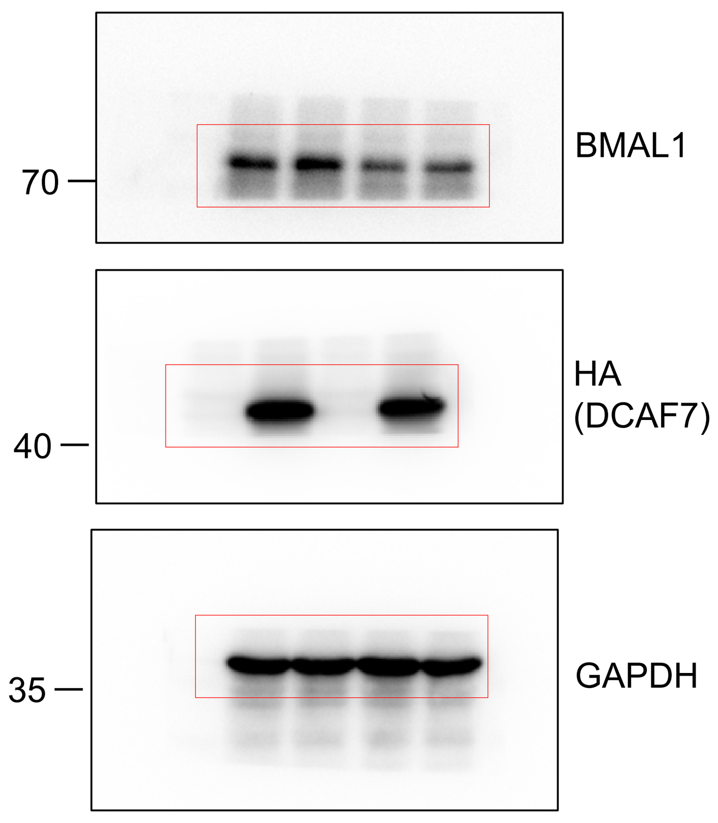

Figure 5L

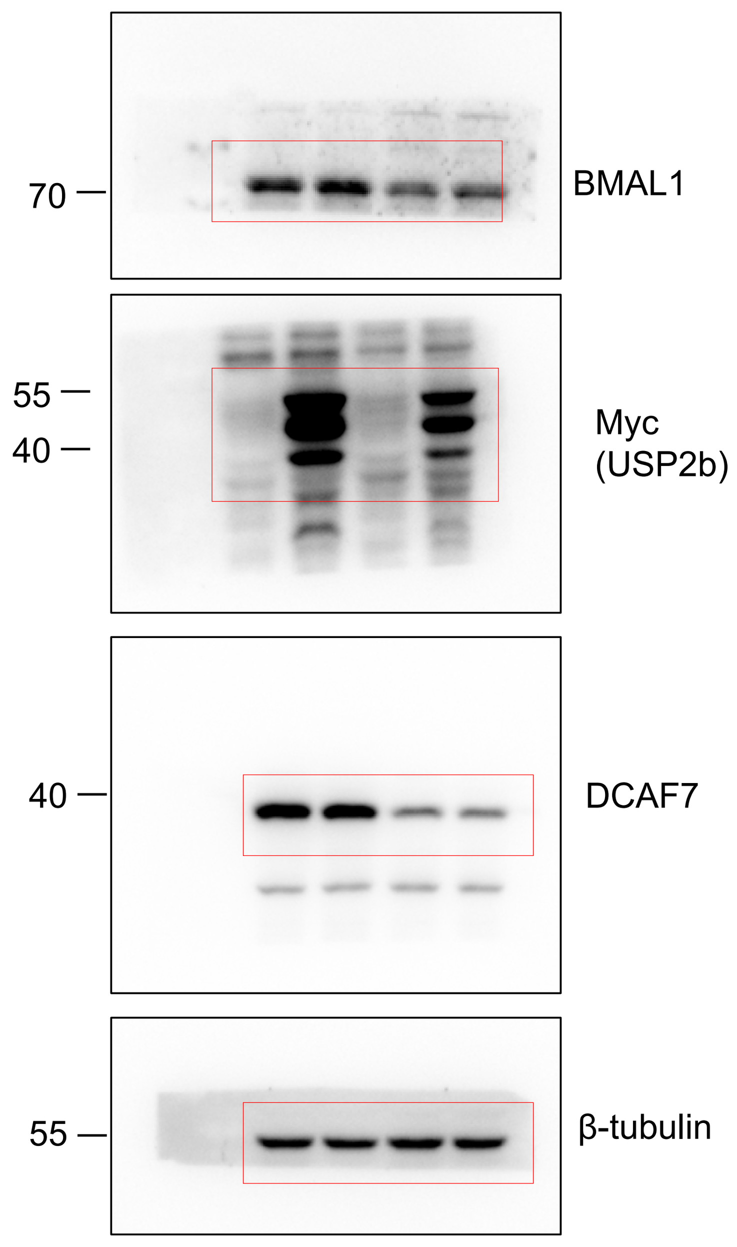

Figure 5M

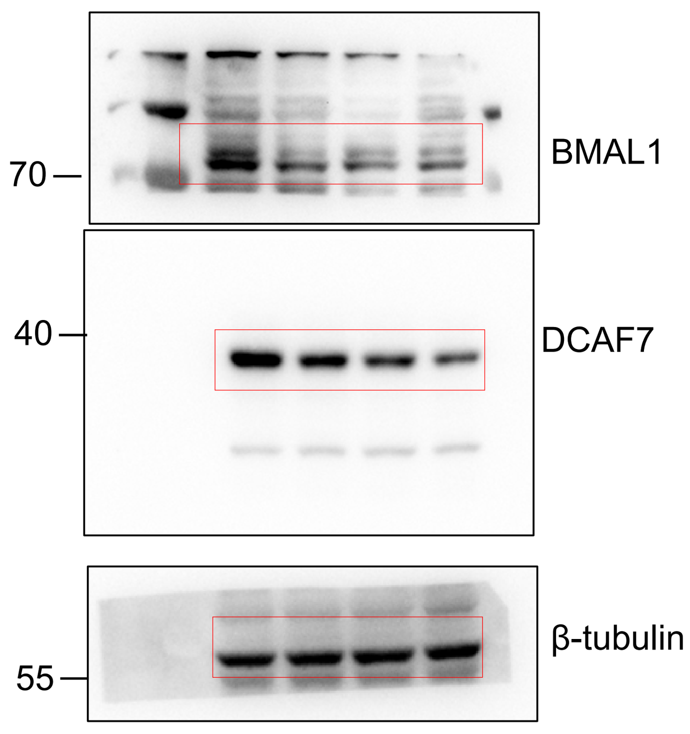

Figure 6A

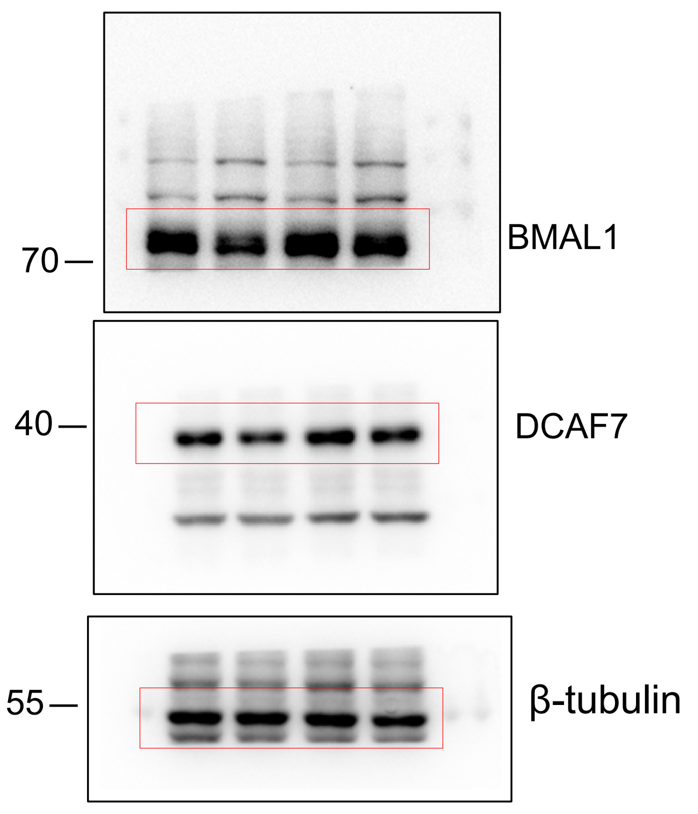

Figure 6B

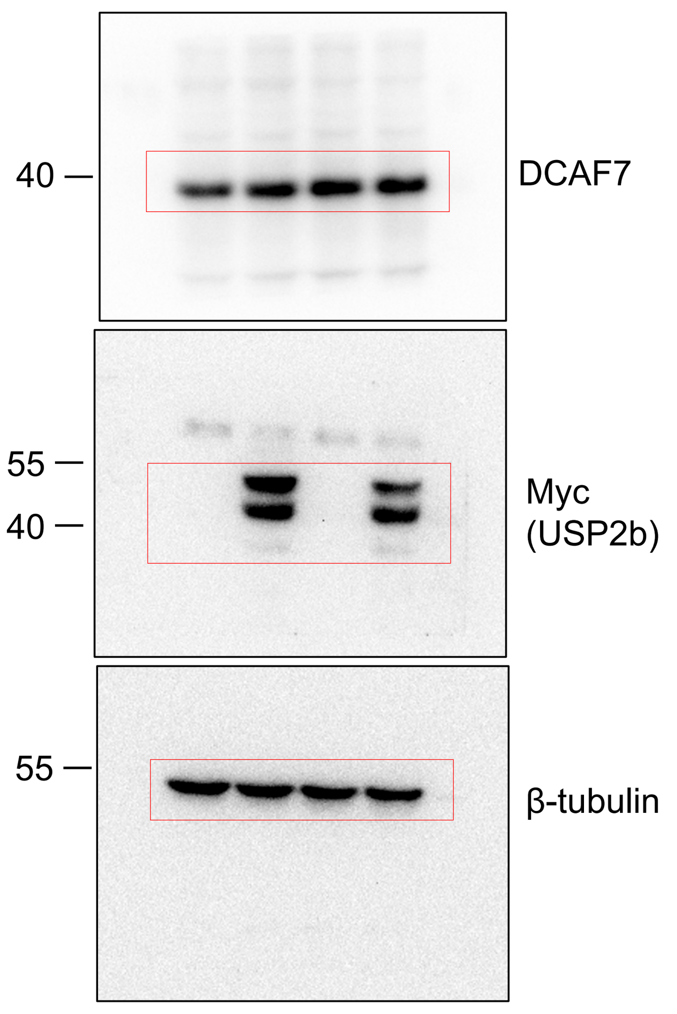

Figure 6C

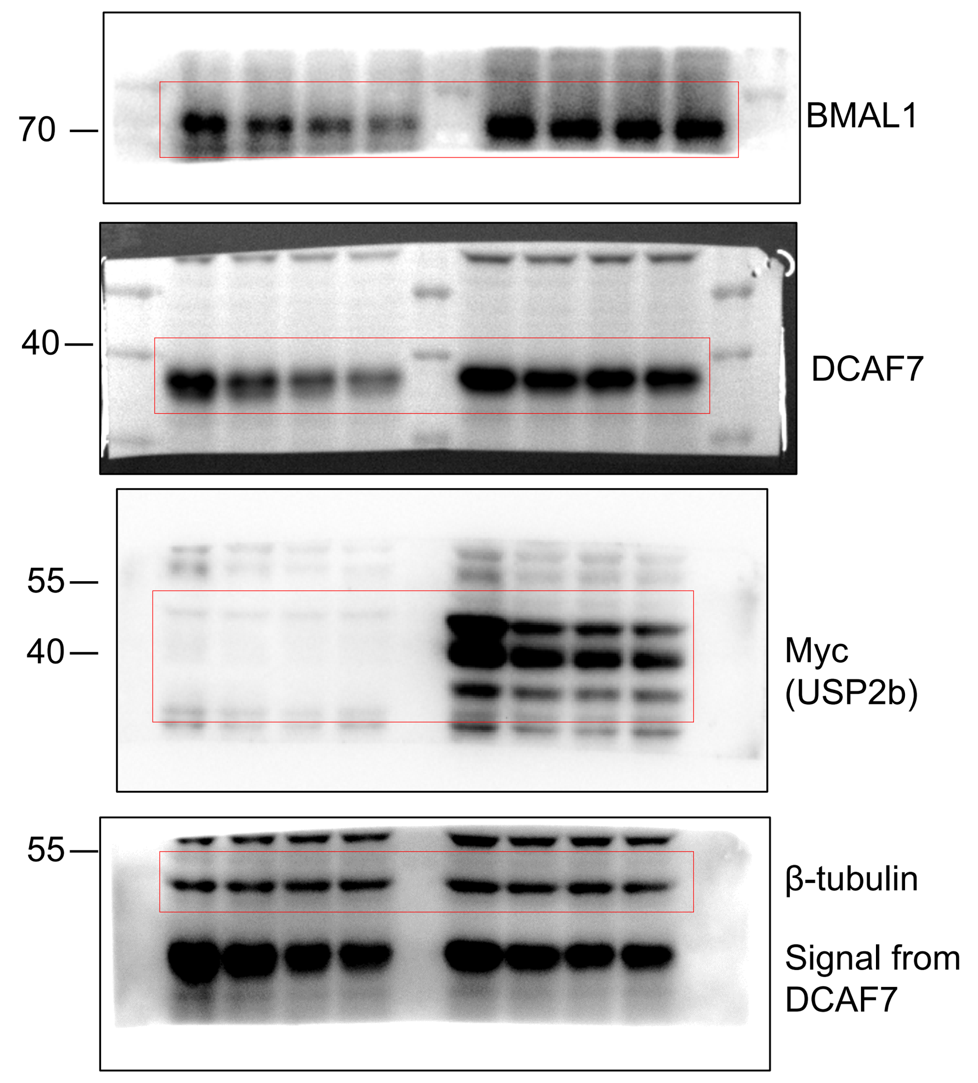

Figure 6D

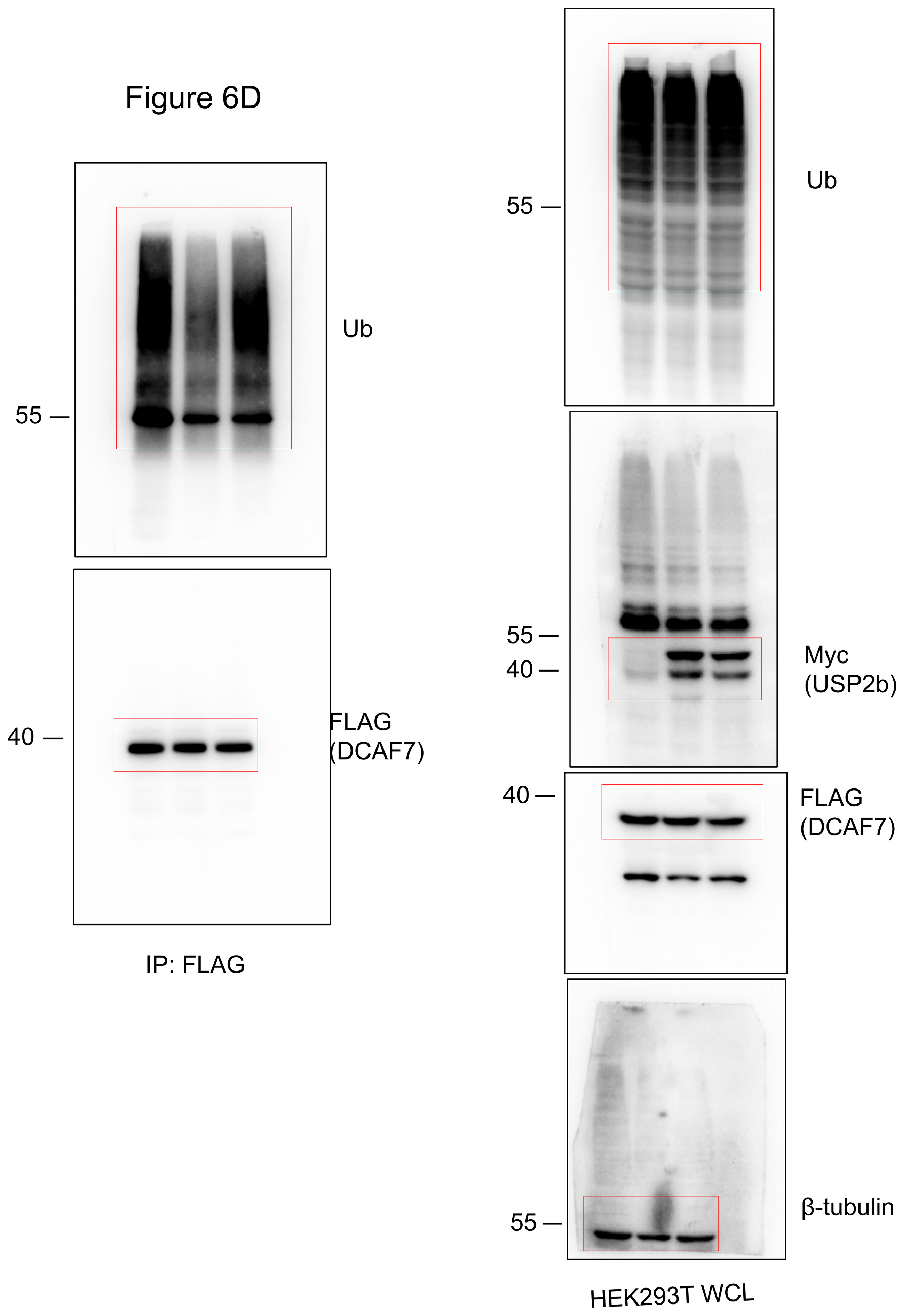

Figure 6E

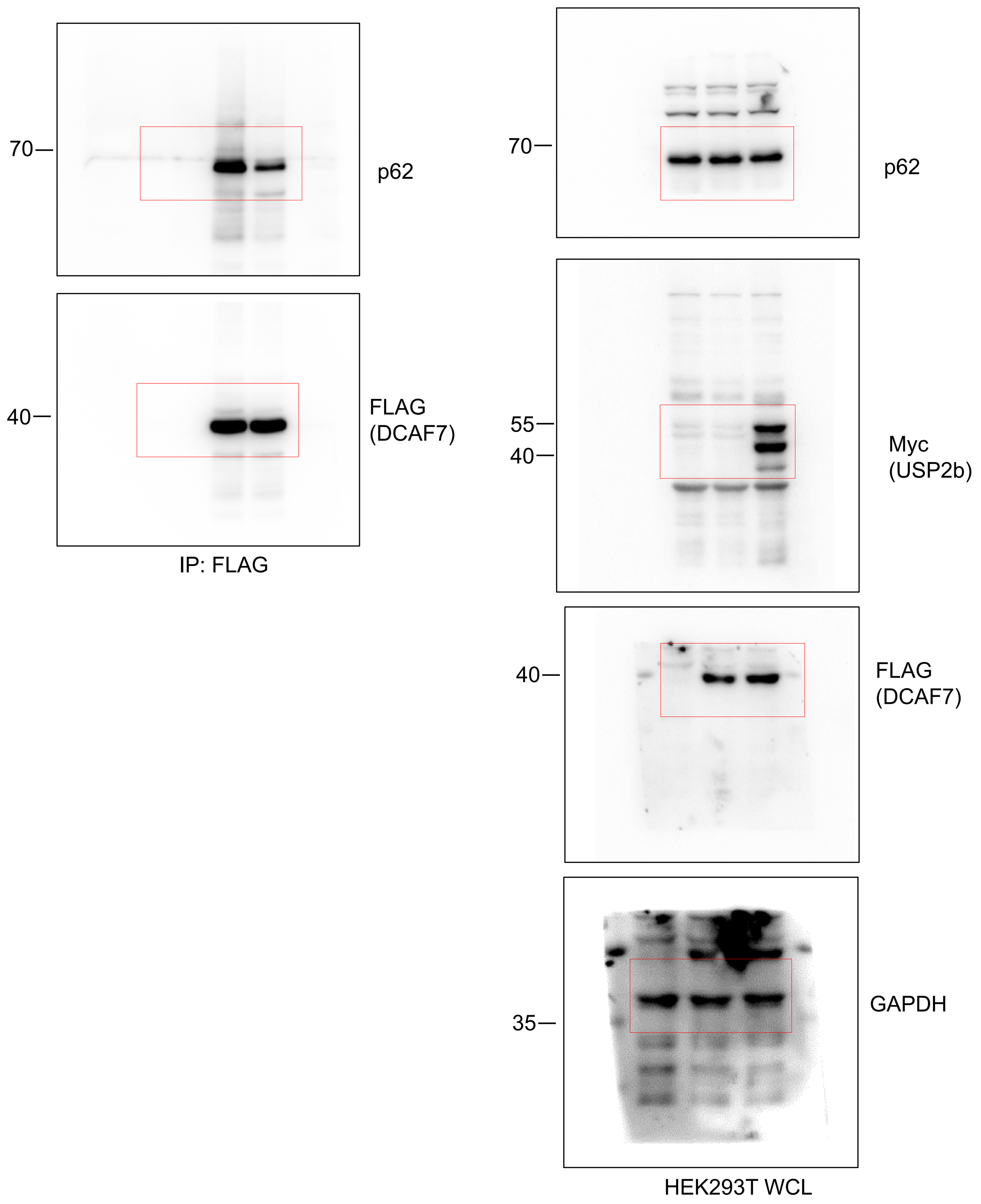

Figure 6F

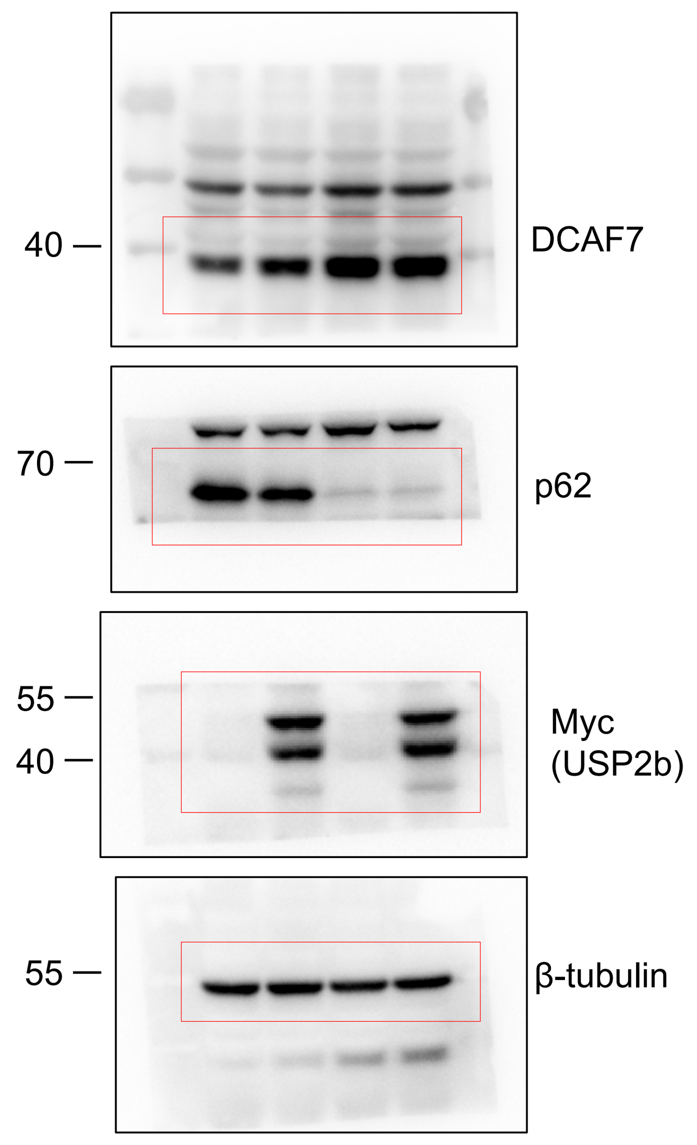

Figure 6G

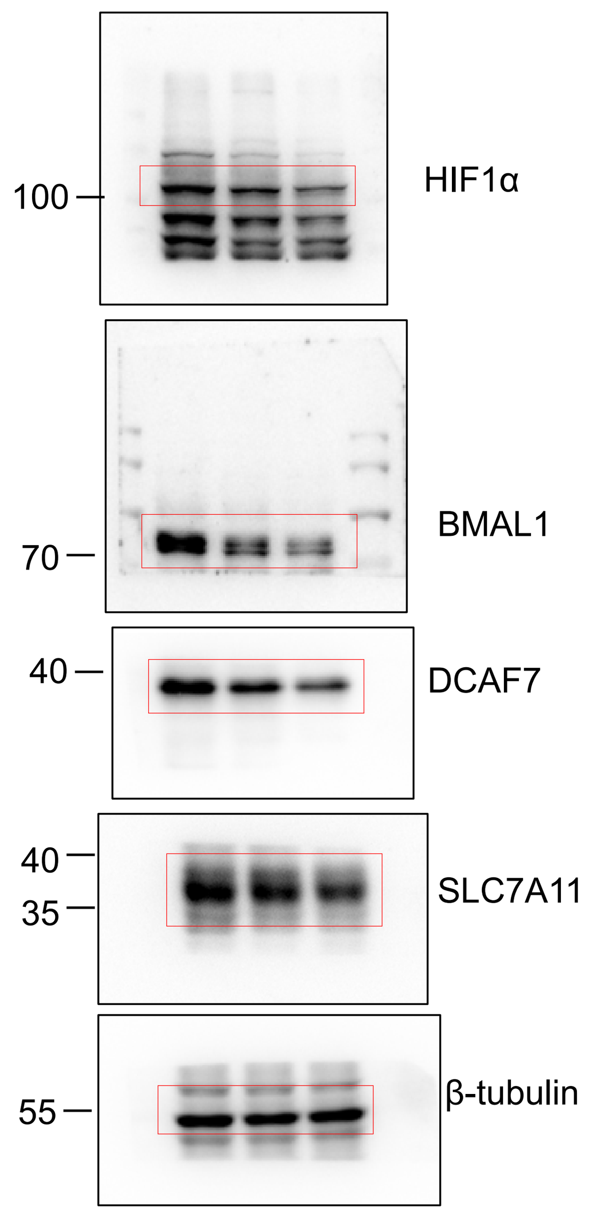

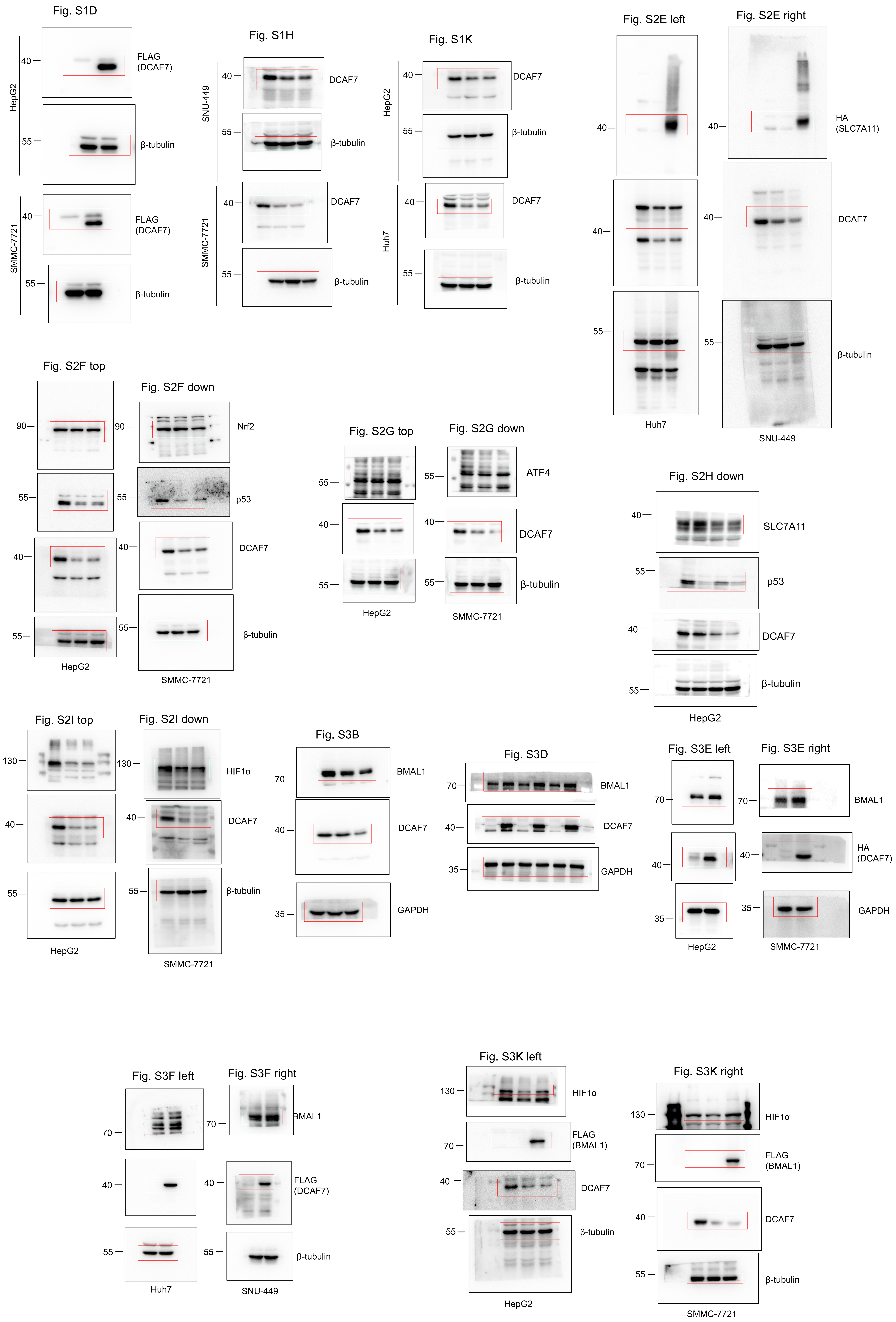
Fig. S1D
HepG2
40
FLAG (DCAF7)
55
β-tubulin
SMMC-7721
40
FLAG (DCAF7)
55
β-tubulin
Fig. S1H
SNU-449
40
DCAF7
55
β-tubulin
SMMC-7721
40
DCAF7
55
β-tubulin
Fig. S1K
HepG2
40
DCAF7
55
β-tubulin
Huh7
40
DCAF7
55
β-tubulin
Fig. S2E left
Fig. S2E right
40
HA (SLC7A11)
40
DCAF7
55
β-tubulin
Huh7
SNU-449
Fig. S2F top
Fig. S2F down
90
Nrf2
55
p53
40
DCAF7
55
β-tubulin
HepG2
SMMC-7721
Fig. S2G top
Fig. S2G down
55
ATF4
40
DCAF7
55
β-tubulin
HepG2
SMMC-7721
Fig. S2H down
40
SLC7A11
55
p53
40
DCAF7
55
β-tubulin
HepG2
Fig. S2I top
Fig. S2I down
130
HIF1α
40
DCAF7
55
β-tubulin
HepG2
SMMC-7721
Fig. S3B
70
BMAL1
40
DCAF7
35
GAPDH
Fig. S3D
70
BMAL1
40
DCAF7
35
GAPDH
Fig. S3E left
Fig. S3E right
70
BMAL1
40
HA (DCAF7)
35
GAPDH
HepG2
SMMC-7721
Fig. S3F left
Fig. S3F right
70
BMAL1
40
FLAG (DCAF7)
55
β-tubulin
Huh7
SNU-449
Fig. S3K left
Fig. S3K right
130
HIF1α
70
FLAG (BMAL1)
40
DCAF7
55
β-tubulin
HepG2
SMMC-7721

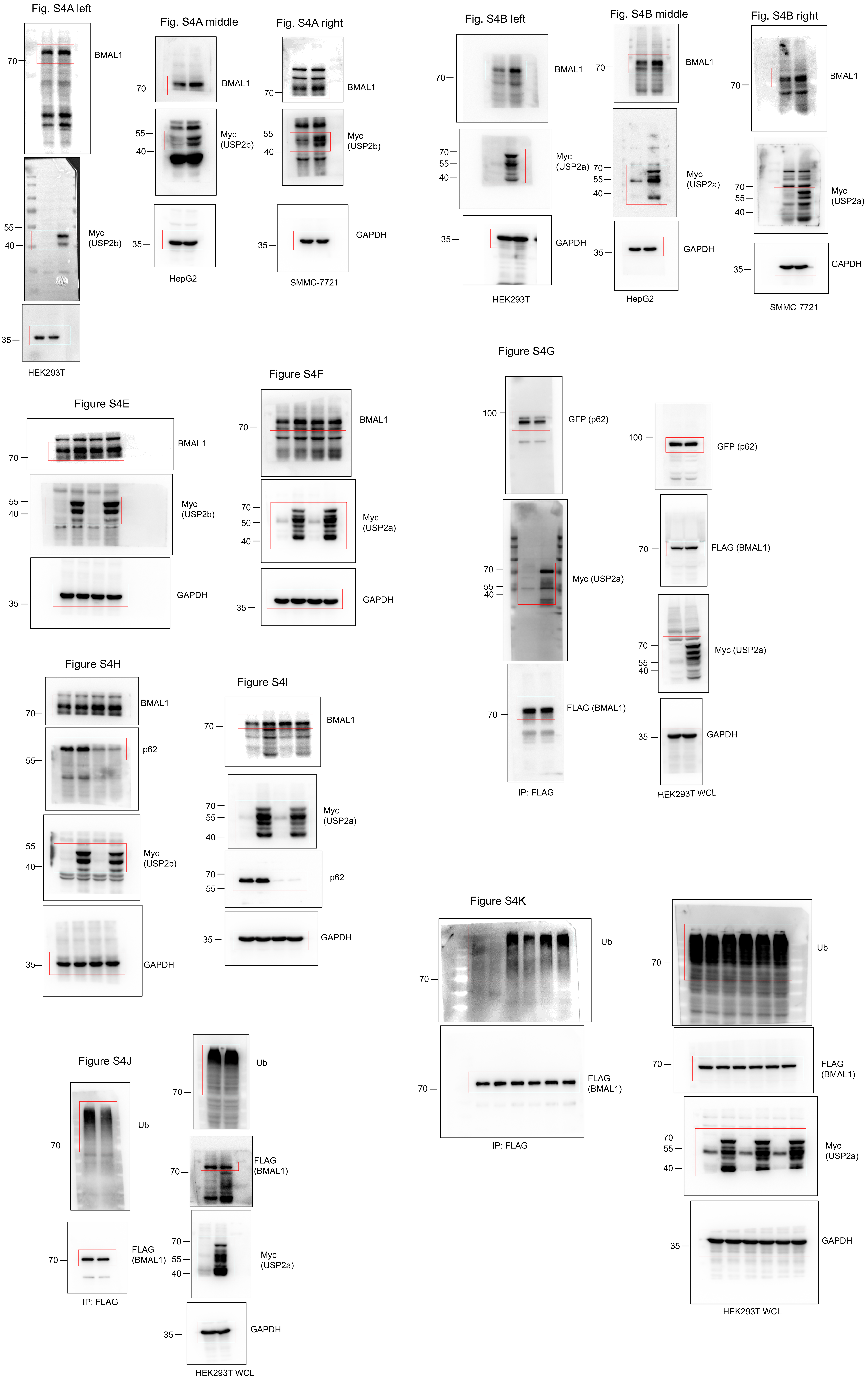
Fig. S4A left
70
BMAL1
55
40
Myc (USP2b)
35
HEK293T
Fig. S4A middle
70
BMAL1
55
40
Myc (USP2b)
35
HepG2
Fig. S4A right
70
BMAL1
55
40
Myc (USP2b)
35
GAPDH
SMMC-7721
Fig. S4B left
70
BMAL1
70
55
40
Myc (USP2a)
35
GAPDH
HEK293T
Fig. S4B middle
70
BMAL1
70
55
40
Myc (USP2a)
35
GAPDH
HepG2
Fig. S4B right
70
BMAL1
70
55
40
Myc (USP2a)
35
GAPDH
SMMC-7721
Figure S4E
BMAL1
70
55
40
Myc (USP2b)
35
GAPDH
Figure S4F
70
BMAL1
70
50
40
Myc (USP2a)
35
GAPDH
Figure S4G
100
GFP (p62)
70
55
40
Myc (USP2a)
70
FLAG (BMAL1)
IP: FLAG
100
GFP (p62)
70
FLAG (BMAL1)
70
55
40
Myc (USP2a)
35
GAPDH
HEK293T WCL
Figure S4H
70
BMAL1
55
p62
55
40
Myc (USP2b)
35
GAPDH
Figure S4I
70
BMAL1
70
55
40
Myc (USP2a)
70
55
p62
35
GAPDH
Figure S4J
70
Ub
70
FLAG (BMAL1)
IP: FLAG
70
Ub
70
FLAG (BMAL1)
70
55
40
Myc (USP2a)
35
GAPDH
HEK293T WCL
Figure S4K
Ub
70
70
FLAG (BMAL1)
IP: FLAG
70
Ub
70
FLAG (BMAL1)
70
55
40
Myc (USP2a)
35
GAPDH
HEK293T WCL

Figure S5C

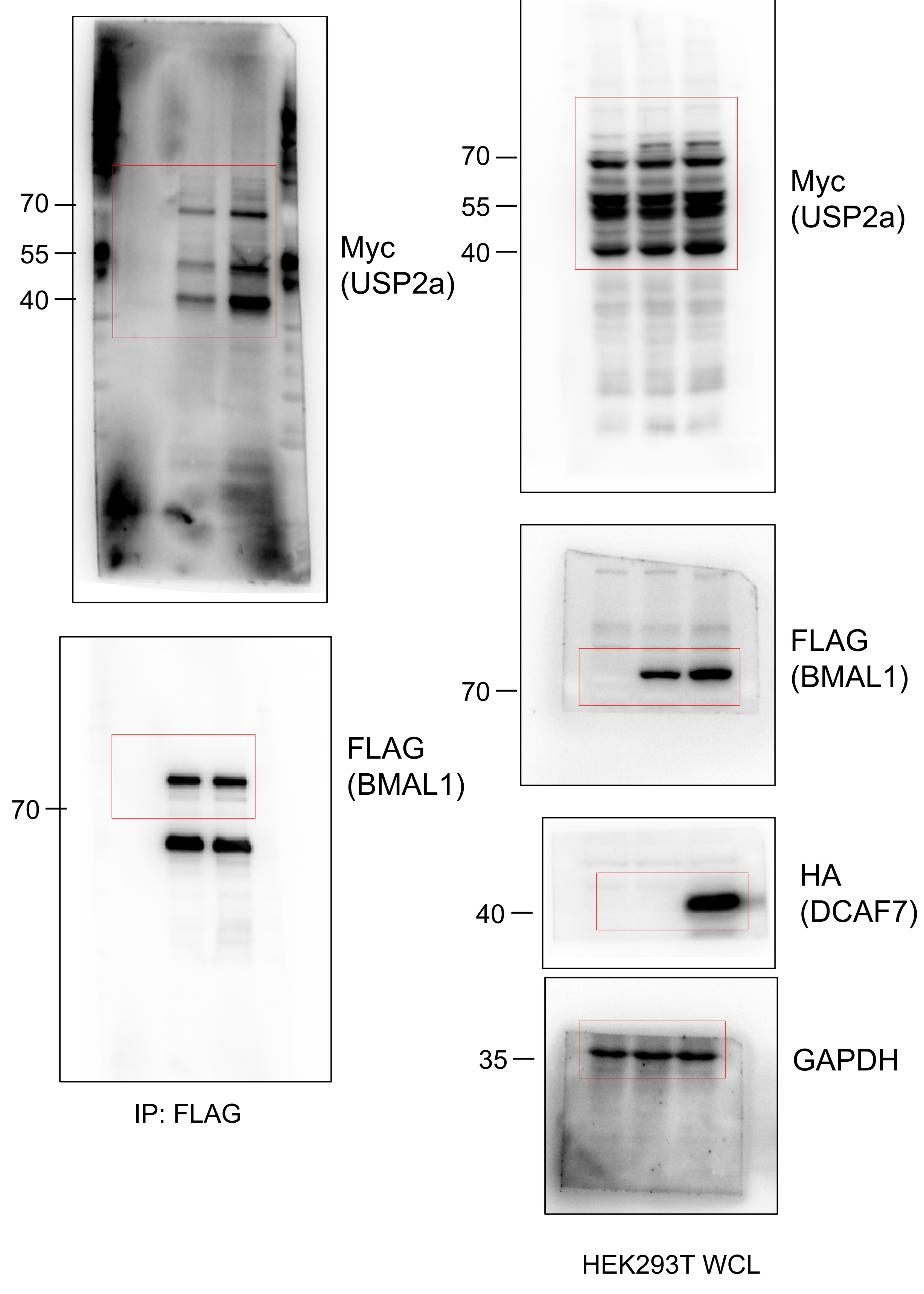

Figure S5D

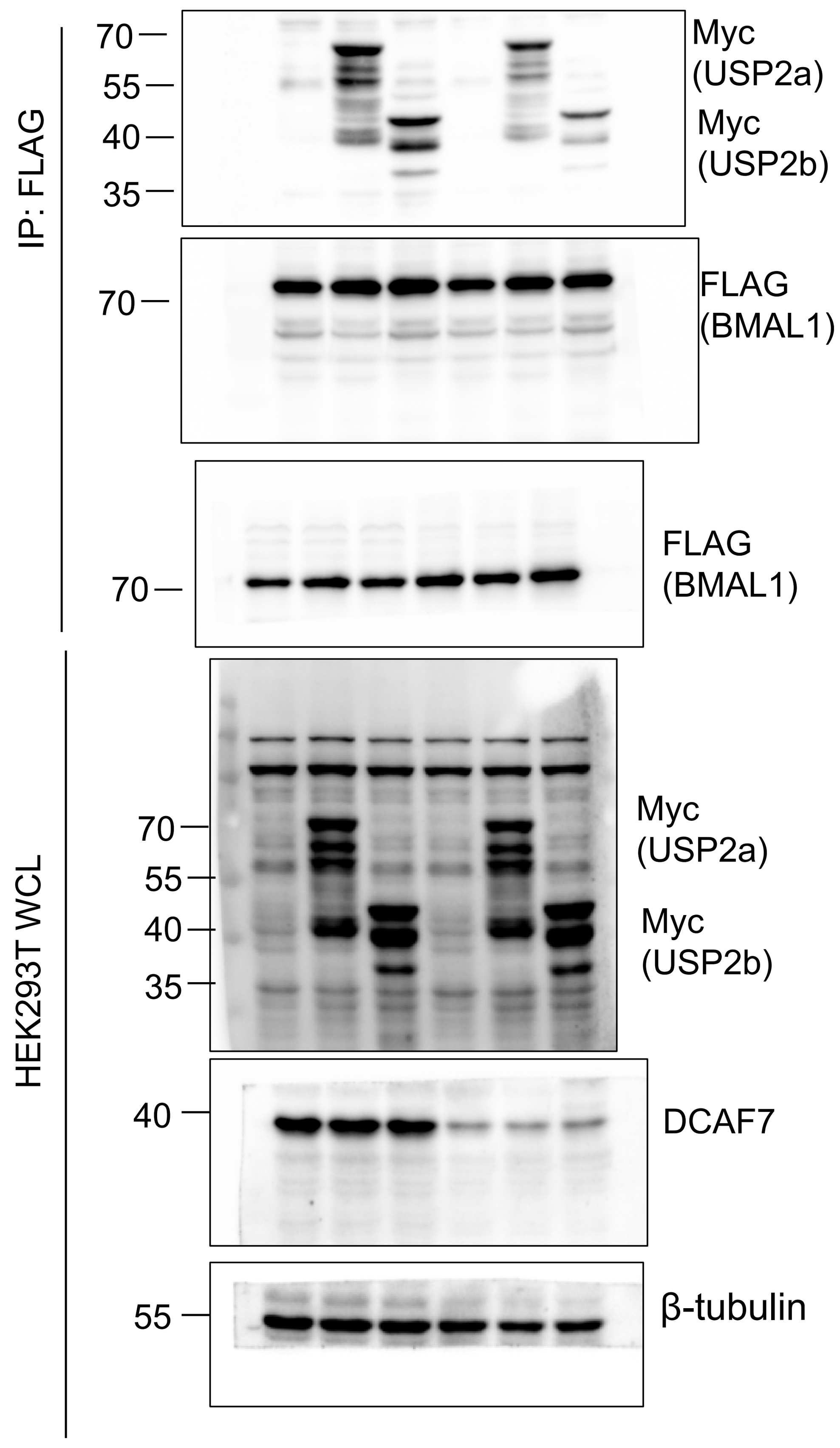

Fig. S5E

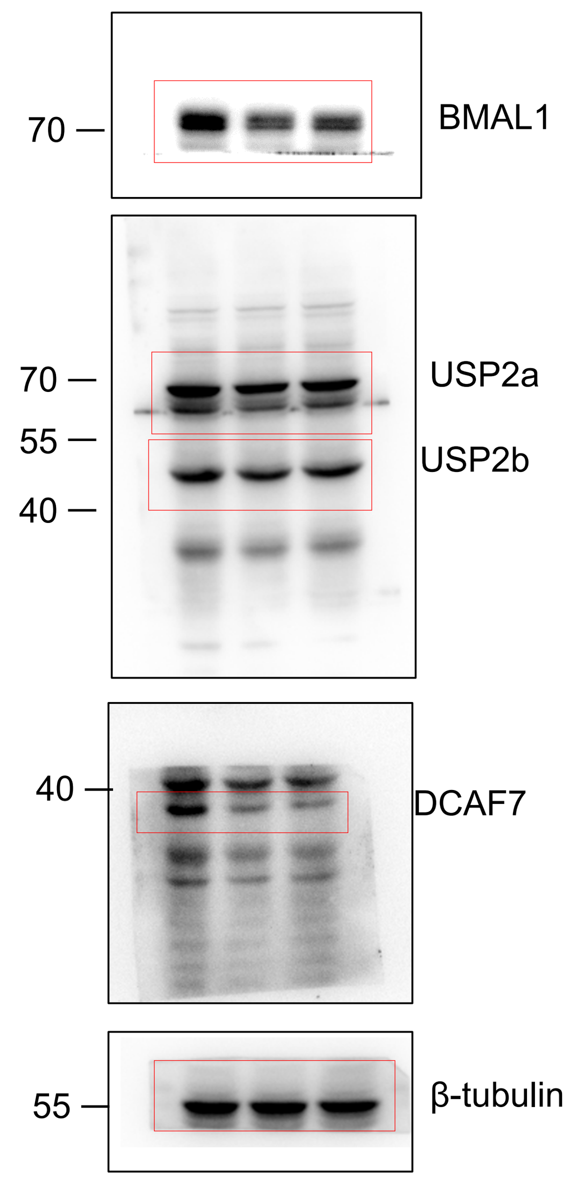

Figure S5F left

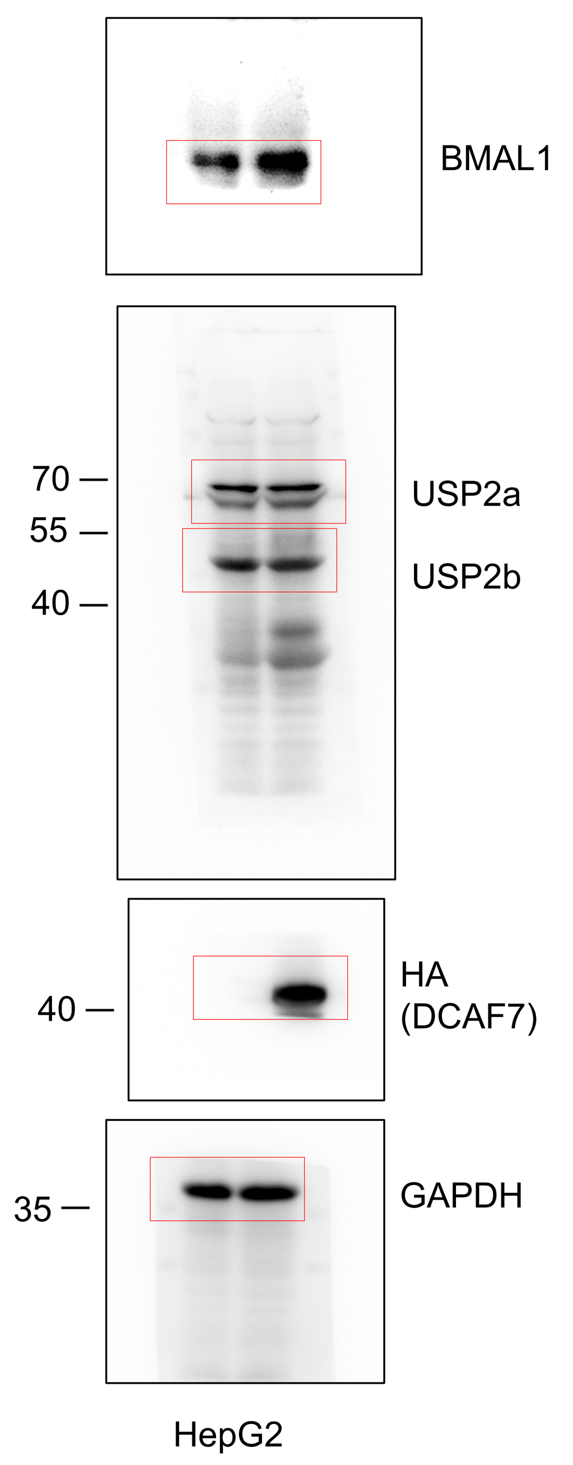

Figure S5F right

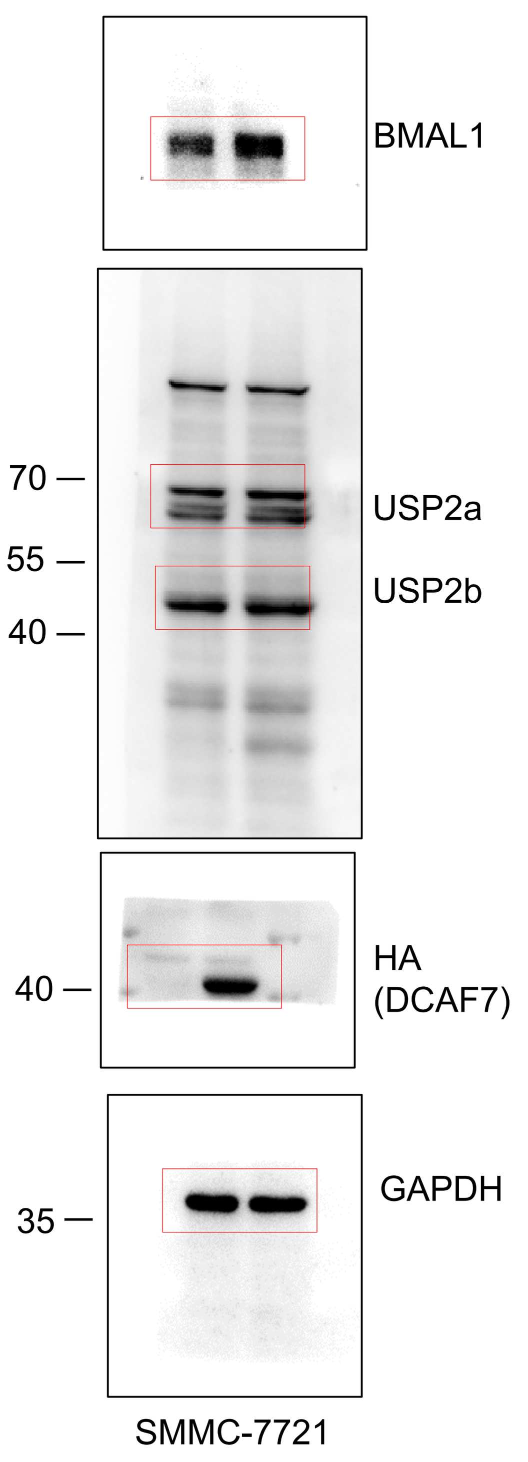

Figure S5G

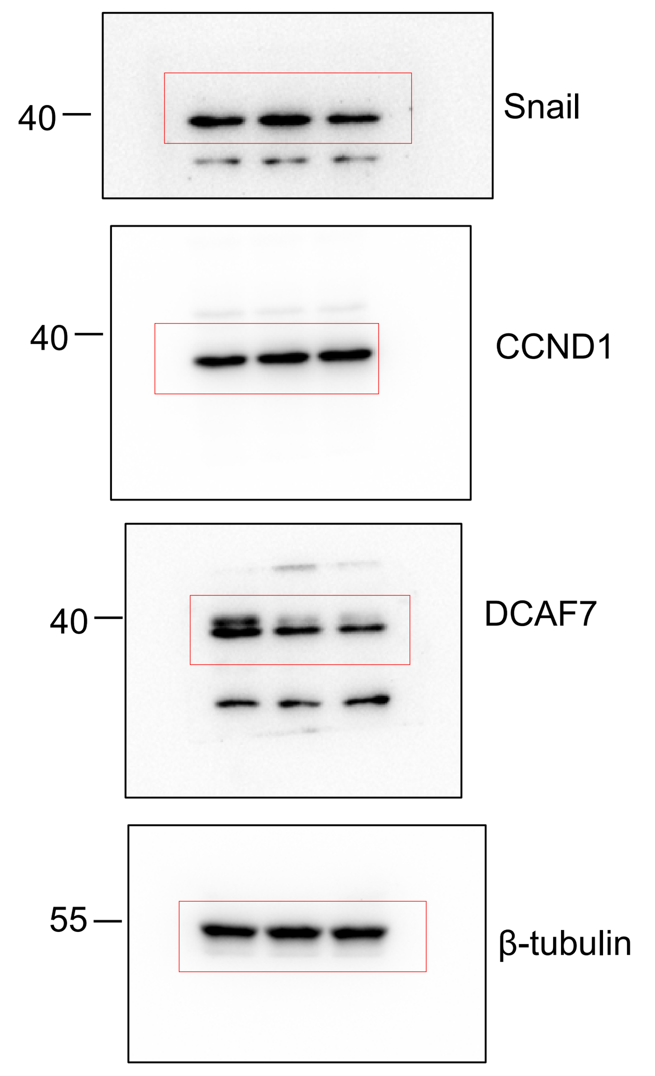

Figure S5H

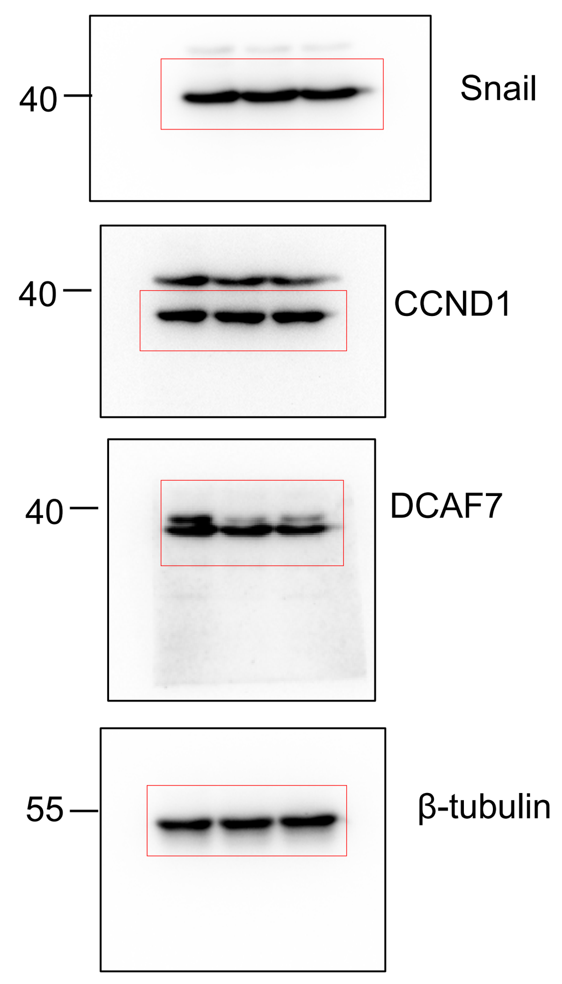

Figure S6A

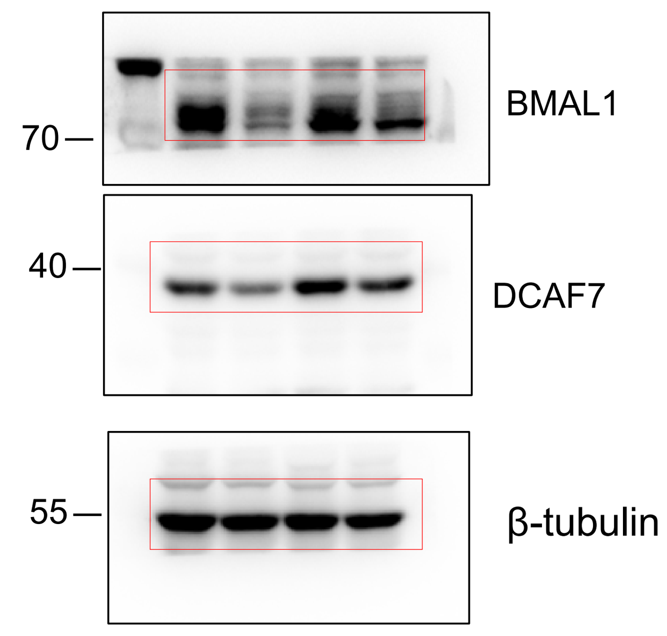

Figure S6D

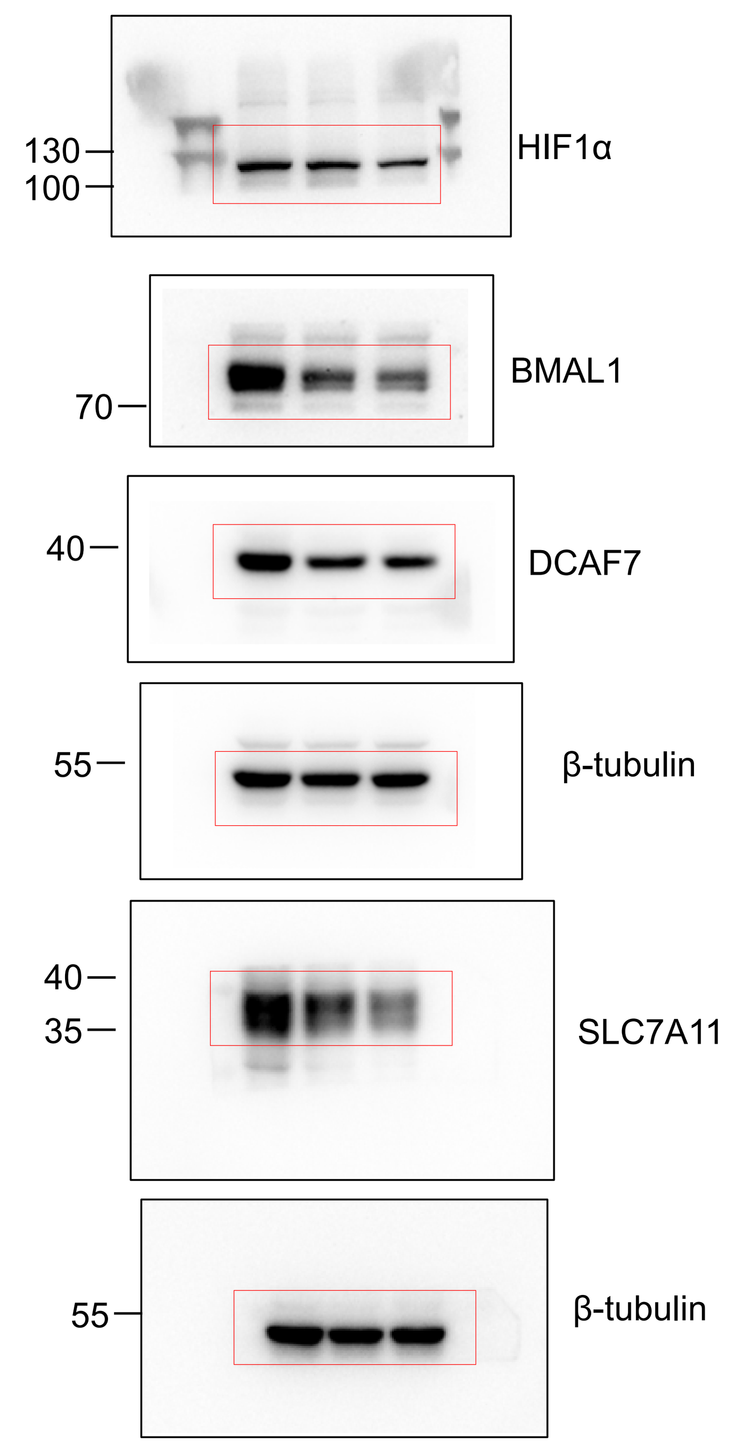

Figure S6C

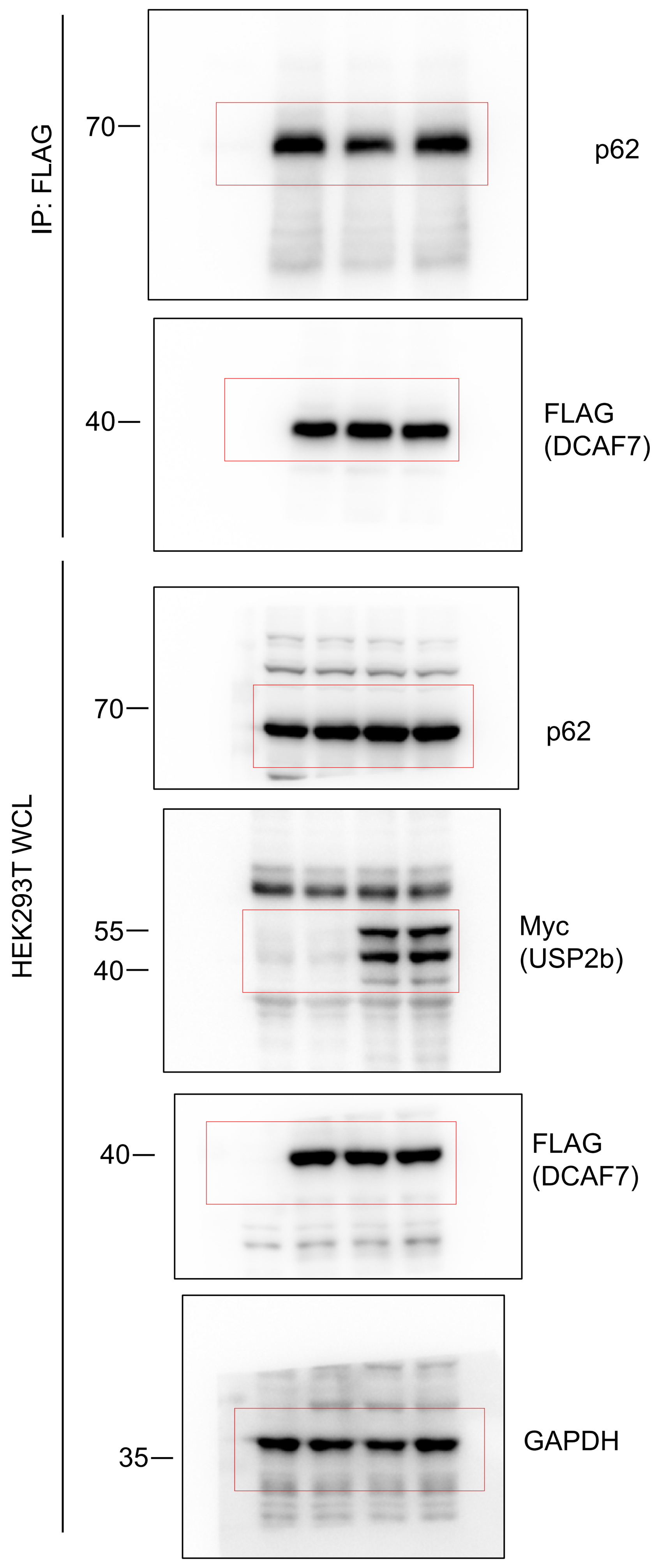

Figure S6B

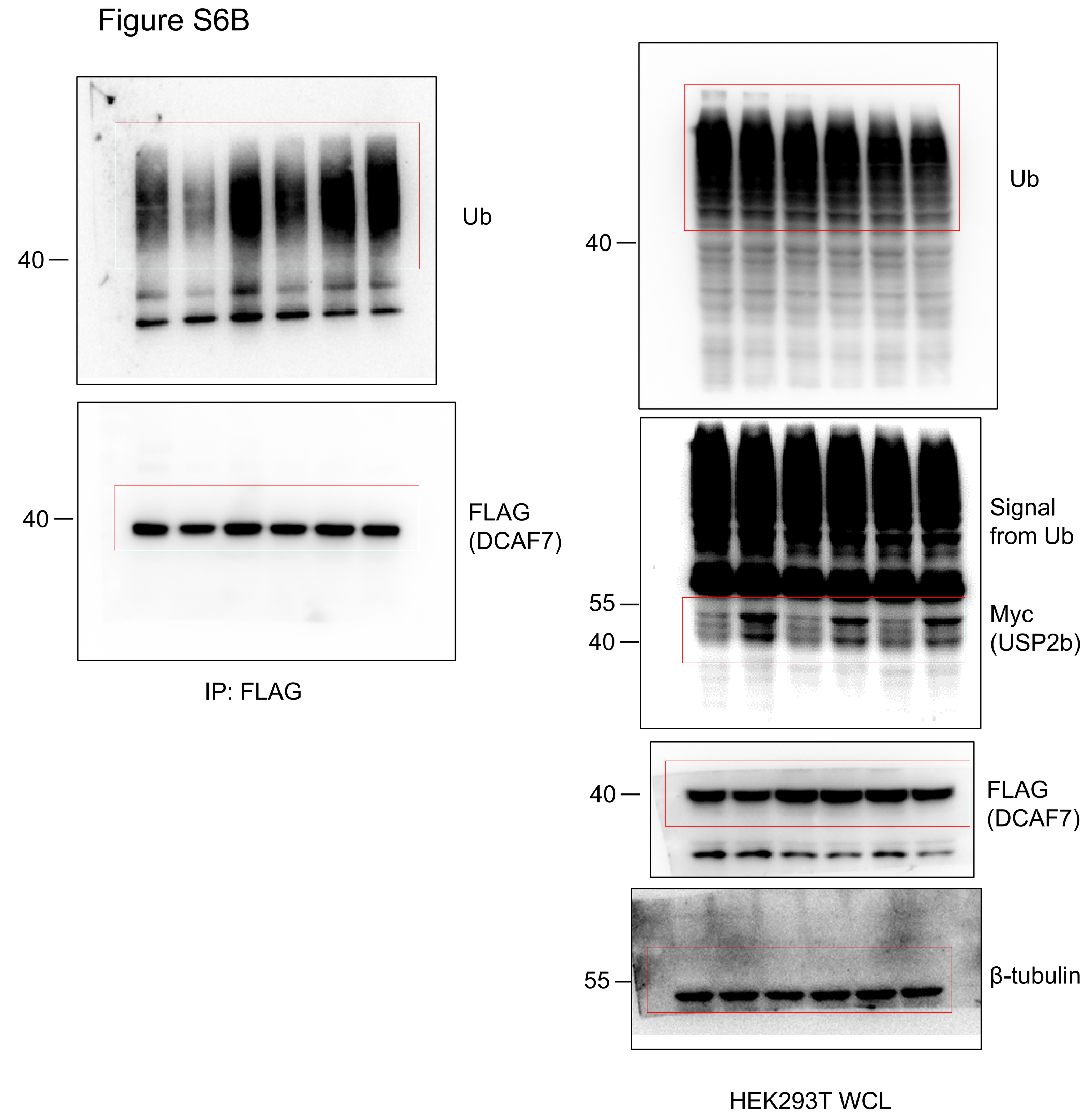

Figure S7C

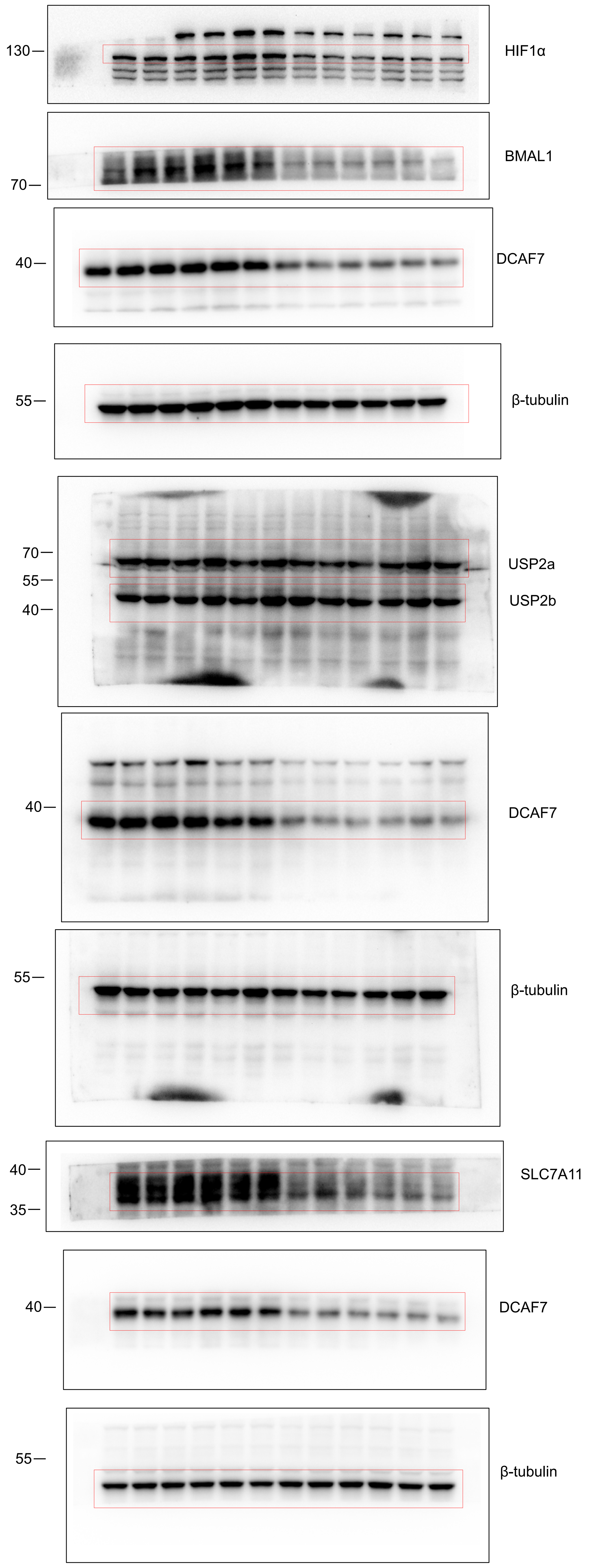

Figure S7G

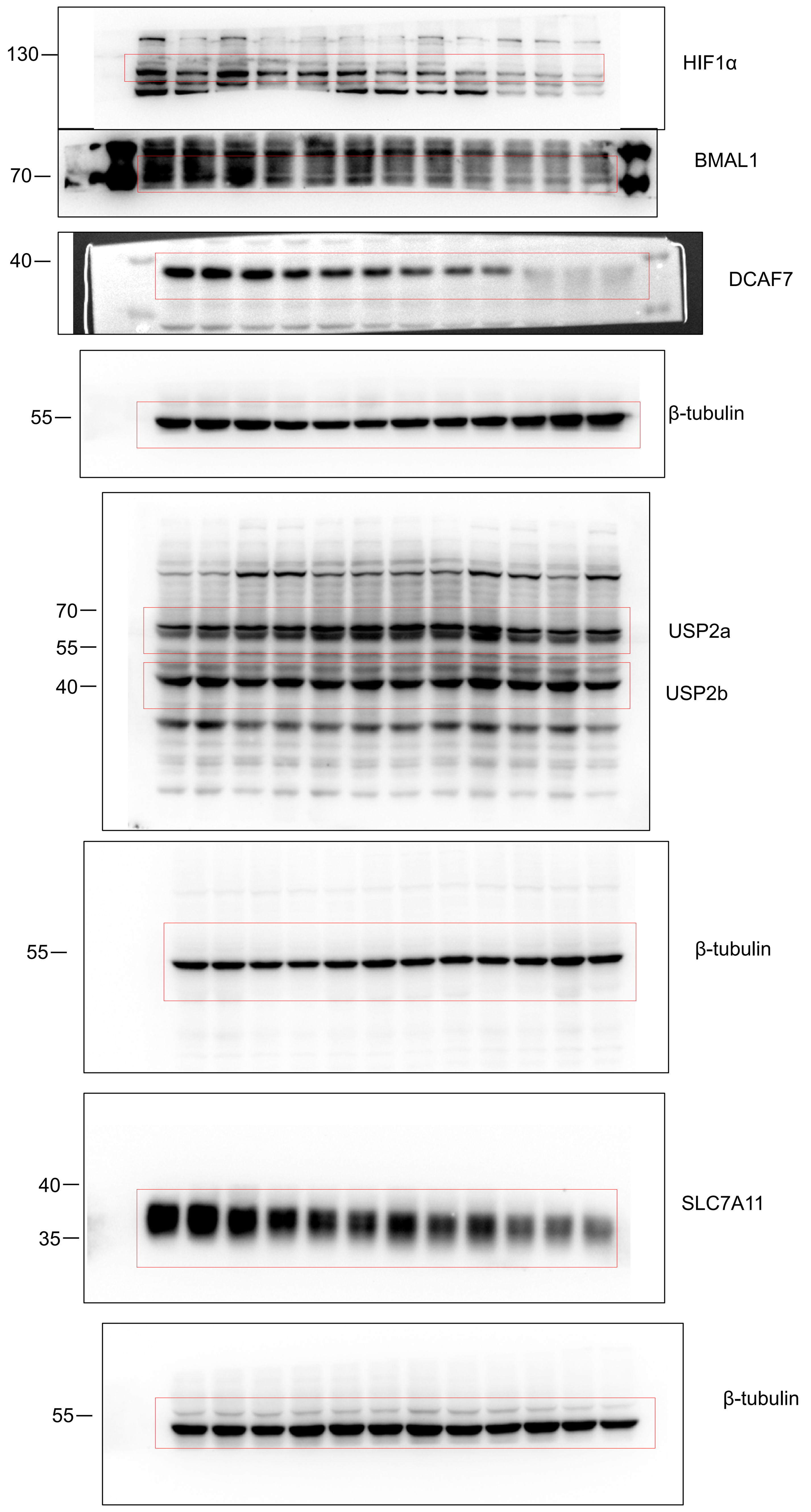

Figure S8A

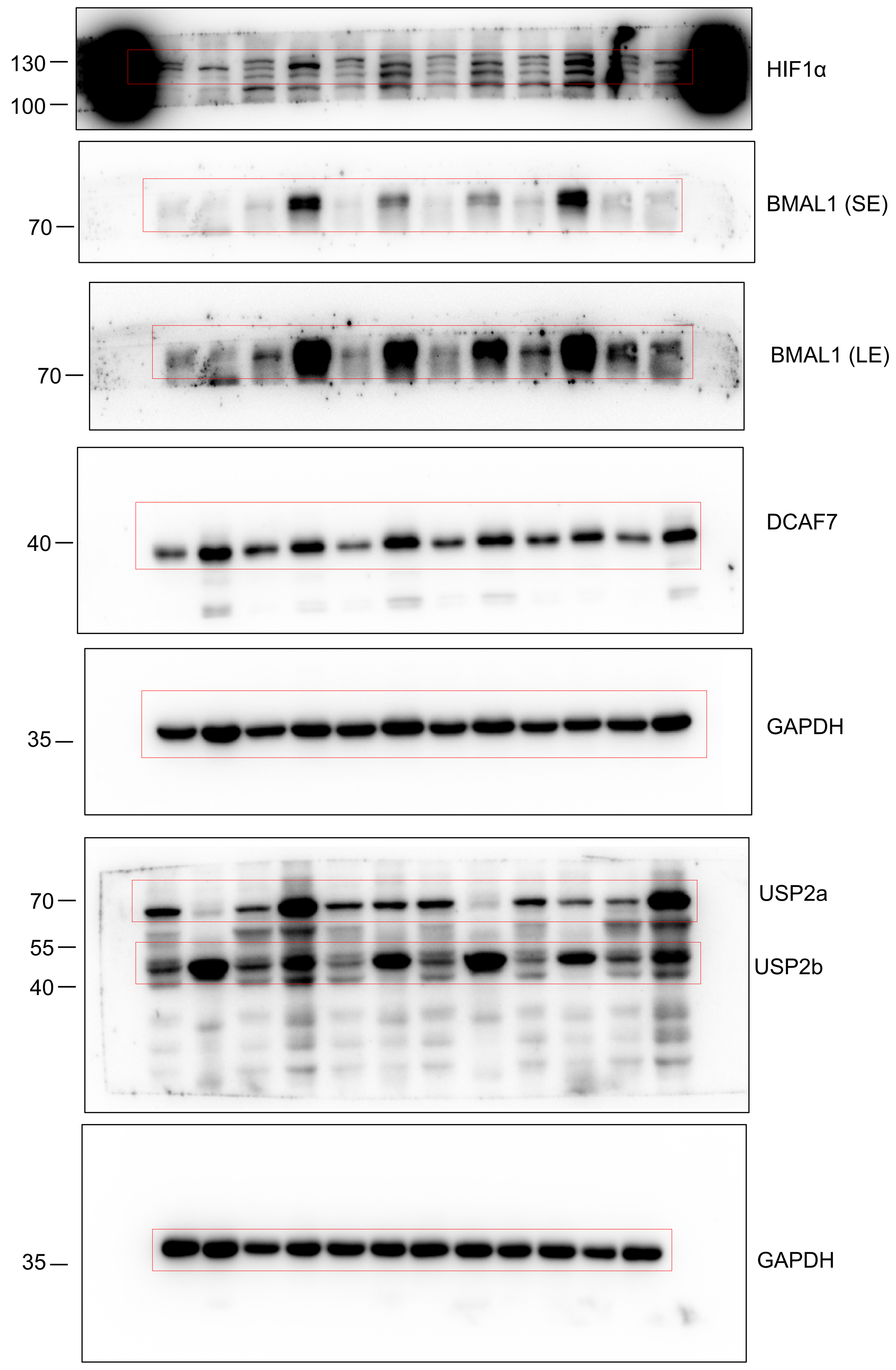

Figure S8B

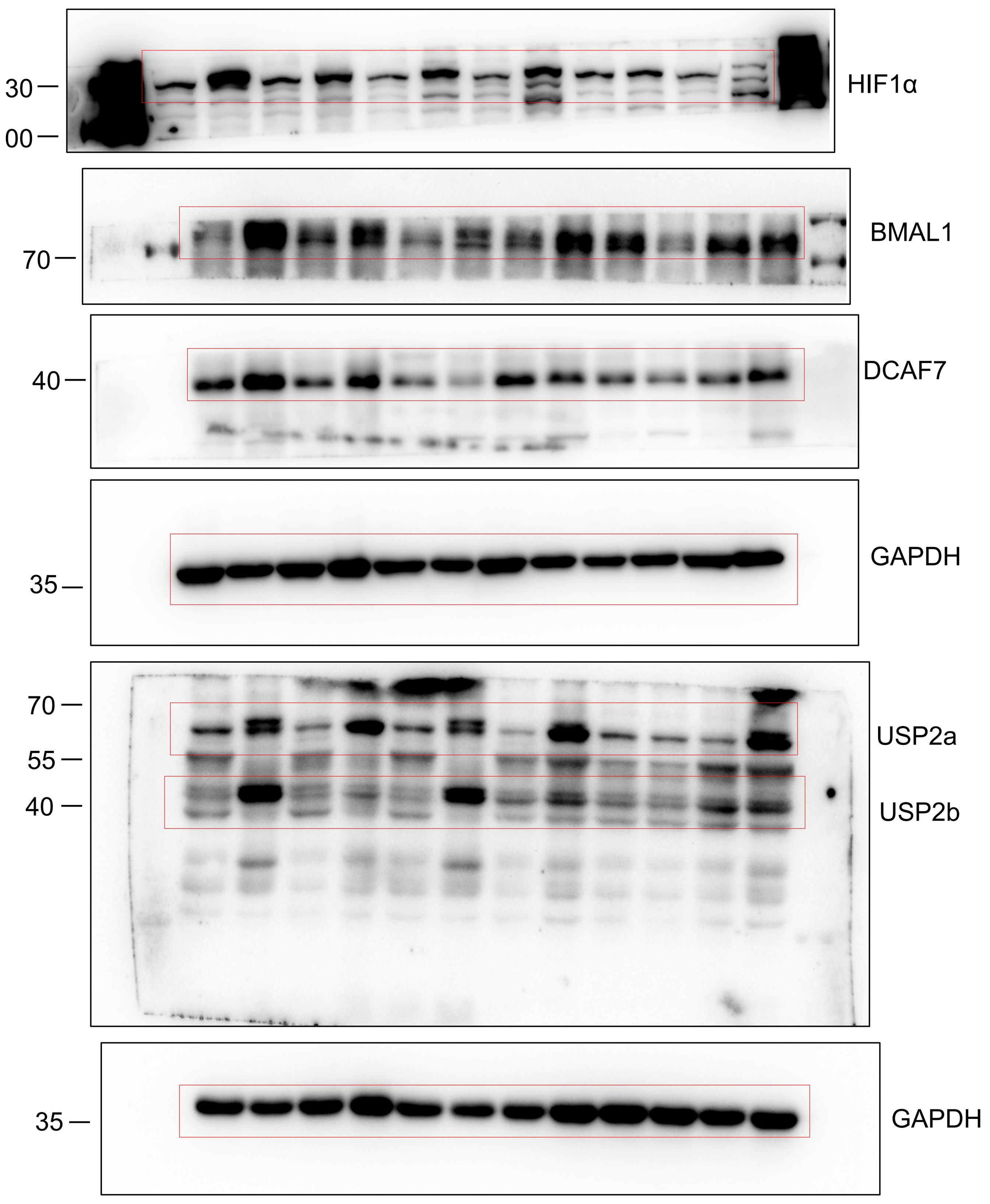

Figure S8C

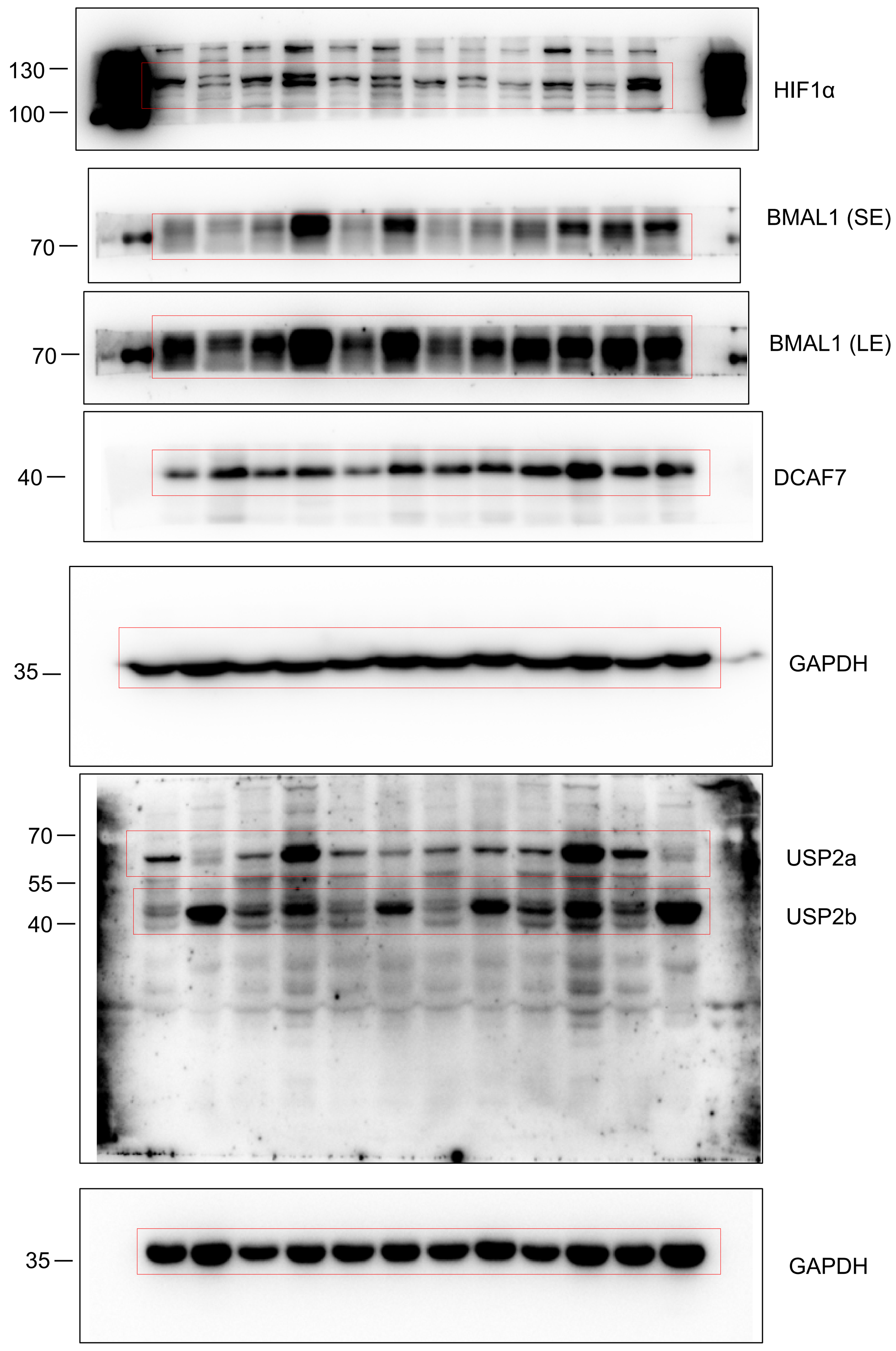

Figure S8D

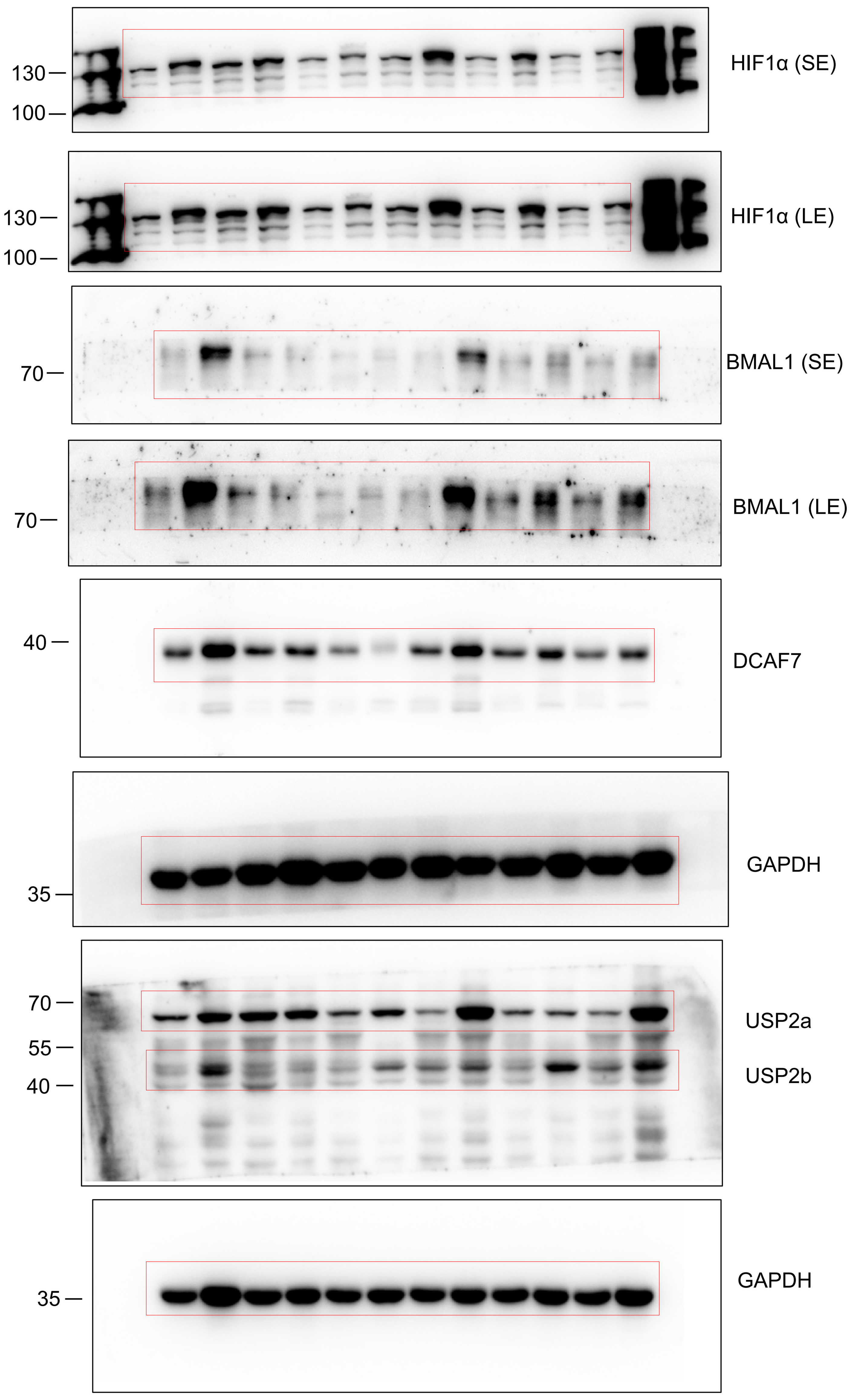

Figure S8E

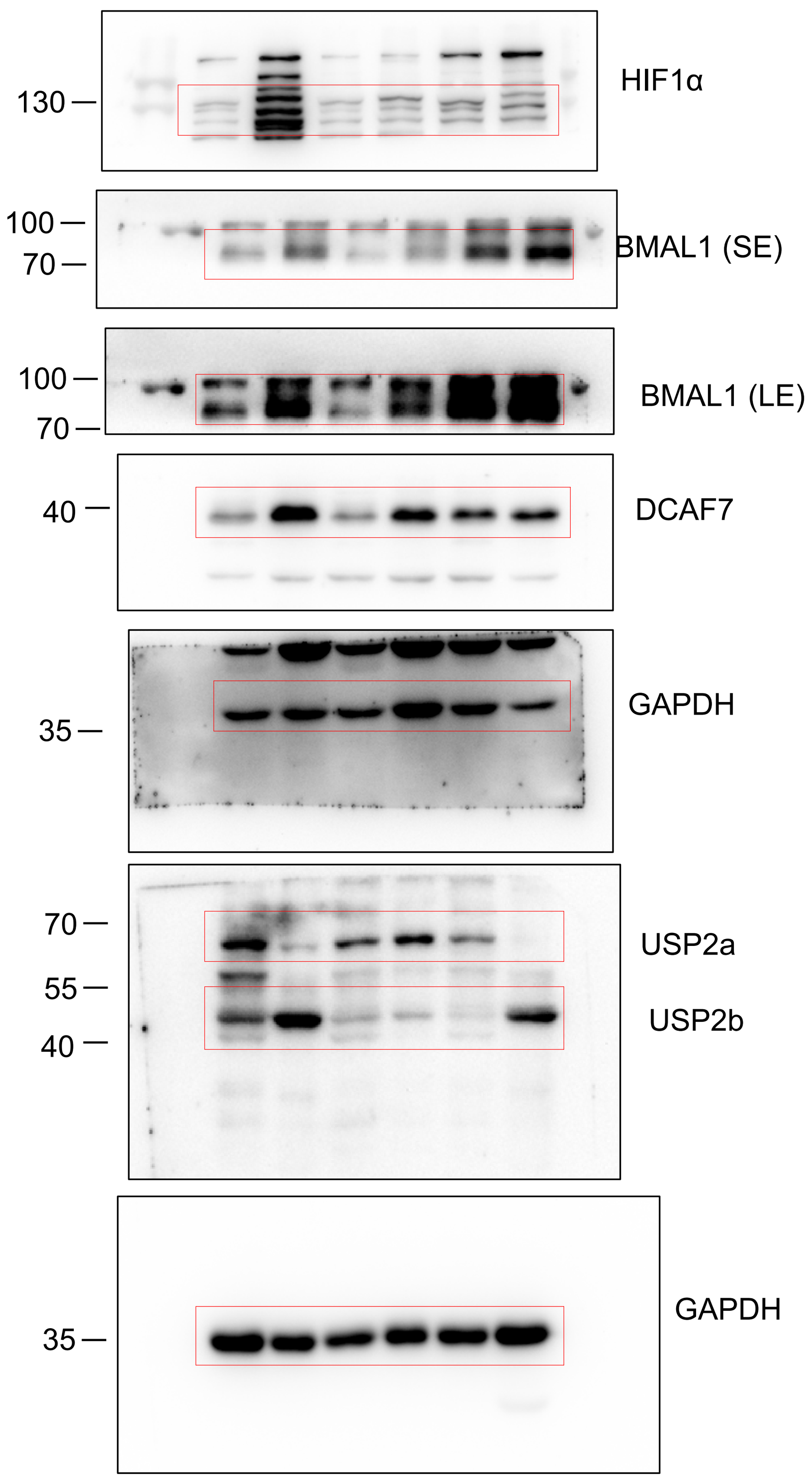

Supplement: Supplementary file 2 — Uncropped blots [file 41419_2025_7977_MOESM2_ESM.pdf]
